# The Effectiveness and Cost-Effectiveness of a Universal Digital Parenting Intervention Designed and Implemented During the COVID-19 Pandemic: Evidence From a Rapid-Implementation Randomized Controlled Trial Within a Cohort

**DOI:** 10.2196/44079

**Published:** 2023-07-27

**Authors:** Melanie Palmer, Nicholas Beckley-Hoelscher, James Shearer, Katarzyna Kostyrka-Allchorne, Olly Robertson, Marta Koch, Oliver Pearson, Petr Slovak, Crispin Day, Sarah Byford, Kimberley Goldsmith, Polly Waite, Cathy Creswell, Edmund J S Sonuga-Barke

**Affiliations:** 1 Institute of Psychiatry, Psychology & Neuroscience, King's College London London United Kingdom; 2 University of Oxford Oxford United Kingdom

**Keywords:** parenting, intervention, digital application, randomized controlled trial, COVID-19 pandemic, mobile phone

## Abstract

**Background:**

Children’s conduct and emotional problems increased during the COVID-19 pandemic.

**Objective:**

We tested whether a smartphone parenting support app, *Parent Positive*, developed specifically for this purpose, reversed these effects in a cost-effective way. *Parent Positive* includes 3 zones. *Parenting Boosters* (zone 1) provided content adapted from standard face-to-face parent training programs to tackle 8 specific challenges identified by parents and parenting experts as particularly relevant for parents during the pandemic. The *Parenting Exchange* (zone 2) was a parent-to-parent and parent-to-expert communication forum. *Parenting Resources* (zone 3) provided access to existing high-quality web-based resources on a range of additional topics of value to parents (eg, neurodevelopmental problems, diet, and sleep).

**Methods:**

Supporting Parents And Kids Through Lockdown Experiences (SPARKLE), a randomized controlled trial, was embedded in the UK-wide COVID-19: Supporting Parents, Adolescents and Children during Epidemics (Co-SPACE) longitudinal study on families’ mental health during the pandemic. Parents of children aged 4 to 10 years were randomized 1:1 to *Parent Positive* or follow-up as usual (FAU) between May 19, 2021, and July 26, 2021. *Parent Positive* provided advice on common parenting challenges and evidence-based web-based resources and facilitated parent-to-parent and expert-to-parent support. Child conduct and emotional problems and family well-being were measured before randomization (T1) and at 1 (T2) and 2 (T3) months after randomization. Service use, costs, and adverse events were measured, along with app use and satisfaction. The primary outcome was T2 parent-reported child conduct problems, which were analyzed using linear mixed regression models.

**Results:**

A total of 320 participants were randomized to *Parent Positive*, and 326 were randomized to FAU. The primary outcome analysis included 79.3% (512/646) of the participants (dropout: 84/320, 26% on *Parent Positive* and 50/326, 15% on FAU). There were no statistically significant intervention effects on conduct problems at either T2 (standardized effect=−0.01) or T3 (secondary outcome; standardized effect=−0.09) and no moderation by baseline conduct problems. Significant intervention-related reductions in emotional problems were observed at T2 and T3 (secondary outcomes; standardized effect=−0.13 in both cases). *Parent Positive*, relative to FAU, was associated with more parental worries at T3 (standardized effect=0.14). Few intervention-attributable adverse events were reported. *Parent Positive* was cost-effective once 4 outliers with extremely high health care costs were excluded.

**Conclusions:**

*Parent Positive* reduced child emotional problems and was cost-effective compared with FAU once outliers were removed. Although small when considered against targeted therapeutic interventions, the size of these effects was in line with trials of nontargeted universal mental health interventions. This highlights the public health potential of *Parent Positive* if implemented at the community level. Nevertheless, caution is required before making such an interpretation, and the findings need to be replicated in large-scale, whole-community studies.

**Trial Registration:**

ClinicalTrials.gov NCT04786080; https://clinicaltrials.gov/ct2/show/NCT04786080

## Introduction

### Background

UK COVID-19 mitigation strategies (eg, extended joint confinement, social isolation, and homeschooling) presented families with unprecedented challenges [1]. Children’s behavioral and emotional problems increased during the pandemic [2], with individuals with preexisting vulnerabilities at particular risk [3]. COVID-19: Supporting Parents, Adolescents and Children during Epidemics (Co-SPACE), a UK-wide general population study, confirmed this pattern during both the first and second UK lockdowns [4,5]. Findings from Co-SPACE and other studies show that increases in parental stress and child-related help seeking were also reported [6,7].

Providing parents with advice and support can reduce children’s conduct and emotional problems [8] and parent-related stress [9]. However, access to face-to-face parenting support interventions was severely limited during the lockdowns. To circumvent this obstacle and reverse pandemic-related increases in children’s conduct and emotional problems, we developed *Parent Positive—*a smartphone app providing evidence-based information and support to the general population. *Parent Positive* had 3 zones. Each was codeveloped with parents and child mental health and parenting experts. Zone 1 was named *Parenting Boosters.* It consisted of 8 elements, or *boosters*, covering content commonly included in standard face-to-face parent training programs. This content was presented in an easy-to-understand format and style (eg, animations, videos, and text resources) tailored to an unguided digital app for use in the general population. In total, 4 of the boosters focused on parental well-being and family functioning (“Staying positive and motivated,” “Making sure everyone knows what is expected of them,” “Keeping calm when your kids act up,” and “Limiting conflict”). One focused on child well-being specifically (“Building your child’s self-confidence and trust”). The remaining 3 focused specifically on improving children’s behavior using classic behavior modification techniques (“Getting your child to follow instructions,” “Promoting better behaviour,” and “Careful use of sanctions”).

The focus for each booster and the parenting challenge it was designed to address were originally selected from a larger library of parent training program content [10]. This selection was informed by parent and child mental health and parenting experts’ views on topics that were especially relevant for parents trying to support their children during the first period of the COVID-19 pandemic. They were initially published on the web in a document entitled *Pointers on Parenting Under Pressure* in the middle of the first UK lockdown in April 2020. Soon after, the 8 *Pointers on Parenting Under Pressure* themes provided the focus for “Families Under Pressure,” a series of short, light-hearted, and celebrity-narrated animations published on social media to promote wider dissemination. These 8 animations formed the basis of the *Parent Positive* boosters.

We further sought information about parenting support needs through a short anonymous questionnaire completed by parents and carers (eg, grandparents). Responses to the questionnaire identified four key features that should be included in the app: (1) downloadable text resources, (2) videos with parenting tips, (3) expert advice and information on how to apply this advice, and (4) experiences shared by other parents. We also conducted 2 web-based focus groups with parents of young children to better understand their views on how support needs could be addressed by the app. These groups indicated that the app should help parents understand why their children behave in a certain way. It should also provide reassurance that all parents struggle at some point when raising a child and many face similar parenting challenges. Parents have asked for tools to help them deal with difficult behaviors and provide a demonstration of how to use these tools in practice. As a parent said, “apps are all about...well...application.” Focus group discussions indicated that content should be snappy and engaging and provided in lay language and by people parents can relate to. Finally, the app should be accessible (eg, videos should have captions) and use visual tools (eg, cartoons and props) to explain any difficult concepts.

As a result of this co-design work with parents, the *Parent Positive* boosters were supplemented by a range of digital material aligned with their focus derived from 2 existing web-delivered parenting interventions: (1) the “Empowering Parents Empowering Communities” (EPEC) parenting program [11,12], concurrently featured on multiple social media channels (eg, Facebook and Instagram: see Center for Parent and Child Support for further information), and (2) the Structured E-Parenting Support app (STEPS) [13]. We also developed 2 further app zones: Parenting Exchange and Parent Resources.

The *Parenting Exchange* (zone 2) was a parent-to-parent and parent-to-expert communication forum. Here, parents interacted with other users by creating posts, commenting on the existing posts, and giving and receiving social feedback through the use of emojis (ie, “I hear you” and “This is helpful”). The forum was moderated by experienced parent group leaders (see the *Methods* section). Parents were also encouraged to ask questions of experts on a variety of child health–related topics (eg, sleep, neurodevelopmental problems, play, language, and schooling). The videos with answers to the questions on a given topic were then posted on the app. Finally, *Parenting Resources* (zone 3) consisted of existing high-quality web-based resources. These concerned topics that were relevant to child mental health, such as anxiety, low mood, bereavement, loss, and trauma, as well as to broader child and family health and well-being, for example, parenting in the digital era, housing and money worries, exercise, and nutrition.

### Objectives

In Supporting Parents And Kids Through Lockdown Experiences (SPARKLE), using a randomized controlled trial (RCT) comparison of *Parent Positive* versus *follow-up as usual* (FAU) implemented within the Co-SPACE cohort, we set out to test the preregistered hypotheses [14] that (1) at 1 month after randomization, *Parent Positive* would reduce parent-reported child conduct (primary outcome) and emotional problems as well as parents’ psychological distress and child-related worries and family conflict more generally, with effects that would persist for 2 months after randomization; (2) reductions in problems would be greater for children with more preexisting conduct problems and higher levels of app use; and (3) *Parent Positive* would be cost-effective compared with FAU.

## Methods

### Patient and Public Involvement

Parents of young children were involved in all aspects of the study, including designing the research, designing the app, participating in the Trial Steering Committee (TSC), and interpreting the results. The SPARKLE Patient and Public Involvement parent panel included 13 members, with 1 parent member who sat on the TSC. The parent panel tested the app before use within SPARKLE and provided electronic feedback on the app and the data collected from the app. We also included parents outside the panel to specifically inform the *Parent Positive* app development (see the *Introduction* section).

### Study Design

SPARKLE was a rapid-deployment 2-arm superiority parallel group RCT of *Parent Positive* versus FAU embedded in the Co-SPACE study (ie, a trial within a cohort). The published protocol is available here [14].

### Participants

Participants were parents or carers of children aged 4 to 10 years. The inclusion criterion for Co-SPACE was parents aged ≥18 years residing in the United Kingdom with a child aged 4 to 16 years. For SPARKLE, only families with children aged 4 to 10 years and access to a compatible smartphone were included. Recruitment took place on the web via email newsletters; parent networks, support organizations, and charities; schools; children’s services departments; and media announcements. Parents of children aged 4 to 10 years who were already in Co-SPACE when SPARKLE started were invited to take part, whereas others could join Co-SPACE and SPARKLE after the trial started. Parents provided separate written informed consent for Co-SPACE and SPARKLE.

### Randomization and Blinding

Participants were allocated to the study arm in a 1:1 ratio by simple randomization automatically at baseline through the *Randomizer* function within Qualtrics (Qualtrics International Inc). The app was downloadable from app stores, with access controlled via a study ID. No sampling blocking or stratification was undertaken. It was not possible to “blind” participants. Senior members (including SB and KG) of the research team remained blind until after the first draft of the analysis report was complete. Staff involved in data collection (MP, OR, and MK), the trial statistician (NBH), and the health economist (JS) were unblinded throughout.

### Ethics Approval

Ethics approval was granted by King’s College London (reference HR-20/21-21,451) and the University of Oxford (references R73153/RE001 for SPARKLE and R69060/RE001 for Co-SPACE).

### Procedures

#### Interventions

##### Parent Positive

The *Parent Positive* app delivers universal parenting information and peer support based on >20 years of evidence from parent-training RCTs [10]. As described in the *Introduction* section, it was designed specifically to help parents during the COVID-19 pandemic. It has 3 zones. The *Parenting Boosters* provided advice on 8 common parenting challenges organized around key themes. The boosters were entitled (1) “Staying positive and motivated,” (2) “Making sure everyone knows what is expected of them,” (3) “Building your child’s self-confidence and trust,” (4) “Getting your child to follow instructions,” (5) “Promoting better behaviour,” (6) “Limiting conflict,” (7) “Keeping calm when your kids act up,” and (8) “Careful use of sanctions.” These are complemented by a range of digital material aligned with their themes derived from the EPEC parenting program [11,12] and the STEPS app [13]. The *Parenting Exchange* provided a platform for parent-to-parent communication facilitated by 6 experienced, trained [12] EPEC parent group leaders who moderated content and responded where appropriate to parent posts. The moderators were paid for their role and received monthly 1-hour group supervision. Supervision consolidated parent moderators’ existing skills and knowledge and adapted these for use within their moderator role in the *Parenting Exchange*. The *Parenting Exchange* also provided opportunities for parents to submit questions to academic and clinical child development and parenting experts, which were then addressed in 9 *Ask the Expert* videos. These included 4 sessions on parenting and behavior management and 1 session each on anxiety, education and learning, neurodevelopmental problems, sleep, and obsessive-compulsive disorder. The third zone, *Parenting Resources*, provided links to carefully selected high-quality web-based resources for child and family well-being.

##### FAU Arm

Parents allocated to FAU received access to *Parent Positive* after the end of the trial.

#### Data Collection

Measures were administered at baseline (T1) and at1 (T2) and 2 (T3) months after randomization through Qualtrics as part of Co-SPACE data collection. *Parent Positive* use data were collected passively through Amazon Web Services. Raw data from the SPARKLE trial will be deposited in the UK Data Service repository [15]. Each participant provided written consent on the web and received two £5 (£1=US $1.26) web-based shopping voucher to thank them for their time completing each follow-up questionnaire. Each participant was assigned a unique study ID that was used to link the deidentified questionnaire and app use data. A data privacy notice was available on the study website.

### Outcomes

#### Clinical Outcomes

The primary outcome was parent-reported child conduct problems using the conduct subscale of the *Strengths and Difficulties Questionnaire* (SDQ) [16] at 1 month after randomization (T2). This is a validated 5-item subscale measuring children’s oppositionality, defiance, and disruptive behavior rated on 3-point Likert scales (*not true*, *somewhat true*, and *certainly true*). An overall subscale score, with higher scores reflecting more problematic behavior, was derived.

Parent-reported SDQ conduct problems score at 2 months after randomization (T3) was a secondary outcome. Other validated secondary outcomes were T2 and T3 measures of (1) parent-reported child emotional problems measured on the SDQ 5-item emotional symptoms subscale and (2) parental psychological distress measured on the *Depression, Anxiety, and Stress Scale (DASS)–21* [17] and multiplied to form a DASS-42 score [14]. In addition, 2 scales used in Co-SPACE measured (1) child-related parent worries about their behavior, well-being, screen time use, education, and the future (previously, Cronbach α was >.54 and test-retest reliability was *r*=0.73) and (2) family conflict related to arguments between parents, parents and children, and siblings as secondary outcomes at both time points [18]. In both cases, a single scale score was created—higher scores indicate more stress and worries and greater family conflict.

#### Health Economic Measures

Retrospective child-related use of health and social care services, including those provided in schools, was measured at T2 and T3 using a modified version of the *Child and Adolescent Service Use Schedule* (CA-SUS) [19]. Unit costs for the financial year 2020-2021 were applied to calculate the total cost of resources used by each participant over the periods from T1 to T2 and T1 or T2 (dependent on T2 completion of data collection) to T3 (Table S1 in Multimedia Appendix 1 [20-24]). *Parent Positive* costs included app development and maintenance costs plus time costs associated with the *Parenting Exchange* operations (full details are provided in Multimedia Appendix 1). Economic outcomes were quality-adjusted life years (QALYs) generated from the 25-item parent-reported SDQ using a crosswalk algorithm to map scores onto the Child Health Utility–9 Dimensions (CHU9D), a pediatric health–related quality-of-life measure [25]. The CHU9D [26] consists of 9 dimensions (sad, worried, pain, annoyed, tired, homework or schoolwork, daily routine, activities, and sleep) rated using 5 levels [25]. QALYs were calculated using the area under the curve approach, which assumed that utility score changes follow a linear path [27]. One QALY is equivalent to 1 year in perfect health. When a child’s quality of life is less than perfect, a QALY can be calculated by multiplying the survival time in the impaired state by the corresponding CHU9D utility value.

#### Intervention Use and Satisfaction

The main *Parent Positive* use measure was total time spent accessing information within each booster by T2 and T3. Other use metrics included the number of times *Parent Positive* was accessed and the number of posts and responses to posts on the *Parenting Exchange*. Satisfaction with *Parent Positive* was measured using a questionnaire developed for SPARKLE based on the average of 3 items measuring the usefulness of each zone rated on a Likert scale from 0 to 6 (*not at all* to *very useful*). Higher scores indicated greater usefulness.

#### Other Measures

Family characteristics and demographic information were collected at baseline. Data on lockdown-related circumstances (eg, being in lockdown) were collected. Adverse events (AEs) were measured using a questionnaire developed for SPARKLE that asked parents to report negative events related to their child’s and their own physical and mental health problems and their relationships or daily activities. The information was coded to categorize the type, severity, seriousness, and relatedness.

### Sample Size Calculation

The target sample size of 616 provided 90% power to detect a Cohen *d* of 0.2 on SDQ conduct problems at T2 (one-tailed; Cronbach α=.05 in favor of *Parent Positive*) assuming 30% attrition and a 0.5 correlation between 1 pre- and 2 postrandomization measures [28]. This effect was judged to be of practical importance as it was equivalent to the approximate 0.2 SD negative effect on conduct problems seen during the pandemic in Co-SPACE.

### Analysis

The *SPARKLE Statistical Analysis Plan* (SAP) and *Health Economics Analysis Plan* can be accessed on the web [15].

### Outcome Analysis

All analyses were carried out using Stata (version 17.0; StataCorp). Baseline and postrandomization app use variables were described by intervention arm and overall, with categorical variables described using frequencies and proportions and continuous variables described using mean and SD or median and IQR as appropriate. App usage between T1 to T2 and T1 to T3 was reported, instead of T1 to T2 and T2 to T3 as specified in the SAP. Differences between arms were assessed using mixed-effects linear models (LMMs) analysis of covariance models, with T2 and T3 measures as dependent variables and a random intercept at the participant level. All models included age, gender (both prespecified), T1 outcomes, intervention arm, time, intervention arm by time interaction, and any additional baseline variables found to be predictive of missingness as fixed effects. Marginal mean *Parent Positive* versus FAU differences at T2 and T3 were extracted from the models, with associated 1-sided 95% CIs and *P* values and a 1-sided type-1 error rate of 5% in favor of *Parent Positive*. See Multimedia Appendix 1 for 2-sided *P* values (as specified in the SAP).

Missing outcome data were summarized; those with at least 1 postrandomization value were included in the LMM models under the intention-to-treat principle. Baseline variables that predicted (at *P*<.05) a binary missingness of any of the primary or secondary outcome variables within a multivariable logistic regression model (with covariates listed for the aforementioned primary analysis) were also included as covariates to make the missing-at-random assumption more plausible and, thus, increase the validity of our intention-to-treat estimate under this assumption [29]. Mean imputation was used for missing data in baseline covariates [30]. Complier average causal effect estimates for those using the app were also conducted (Multimedia Appendix 1). The moderating effect of T1 conduct problems on conduct problem intervention effects at T2 and T3 was assessed by adding a time-by-treatment-by-baseline conduct interaction term to the LMM analysis of covariance model for this outcome. Assessing moderation by postrandomization app use by principal stratification models was not possible as we did not have sufficiently strong predictors of app use [31].

Medical (physical and psychological) and nonmedical familial AEs (eg, reduction in school attendance) and serious AEs were summarized.

### Economic Analysis

Between-arm differences in costs and QALYs were analyzed using generalized linear models with bootstrapped 95% CIs adjusted for prespecified T1 covariates and CHU9D scores. Adjustment for differences in T1 costs was not possible as service use data were not collected in Co-SPACE; the CA-SUS was administered at T2 and T3 only. The primary economic analysis was a cost-utility analysis at T2. Costs were calculated from the National Health Service and personal social services perspective. Effects were measured in terms of QALYs. Incremental cost-effectiveness ratios were calculated, and nonparametric bootstrapping was used to propagate sampling uncertainty surrounding the mean incremental cost-effectiveness ratios by generating 1000 estimates of incremental costs and QALYs [32]. Cost-effectiveness acceptability curves demonstrated the probability that *Parent Positive* was cost-effective compared with FAU using the National Institute for Health and Care Excellence cost-effectiveness threshold of £20,000 to £30,000 per QALY [33]. Following standard practice, outliers with costs in the 99th percentile [34] were excluded from the analysis on the grounds that they may distort cost-effectiveness findings given that it was not possible to adjust for baseline differences in costs. Sensitivity analyses explored the effect of missing cost and QALY data (using multiple imputation with chained equations) [35] and the effect of replacing QALYs with the primary clinical outcome. A prespecified secondary economic analysis explored cost-utility at T3.

### Oversight of the Trial

The independent TSC, consisting of clinicians, statisticians, health economists, policy makers, and Patient and Public Involvement representatives, met every 4 months. No interim analyses were undertaken. The role of the Data Monitoring and Ethics Committee was taken over by the TSC.

## Results

### Recruitment and Retention

**Figure 1:**
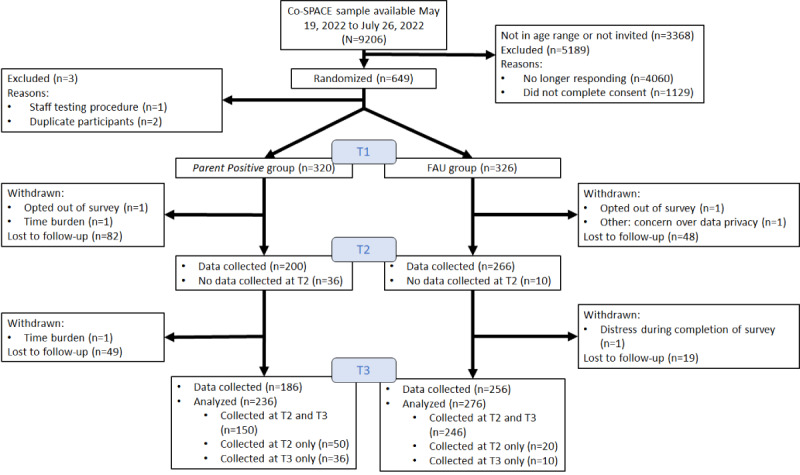
CONSORT (Consolidated Standards of Reporting Trials) diagram. No longer providing=no Co-SPACE follow-up data. Exclusions: a member of staff signed up to test the randomization system. Early in the study, 2 individuals accessed the platform from 2 different devices on 2 different occasions each with the same email address, completed the baseline surveys, and were randomized. Procedures were then put in place to rectify this, and only the first set of baseline data and randomized allocation were used. Co-SPACE: COVID-19: Supporting Parents, Adolescents and Children during Epidemics; FAU: follow-up as usual.

Between May 19, 2021, and July 26, 2021, a total of 646 parents were recruited, of whom 320 (49.5%) were assigned to *Parent Positive* and 326 (50.5%) were assigned to FAU. This was 5% more than planned because of the inherent imprecision of the closing of web-based recruitment. Retention at T2 (466/646, 72.1%) and T3 (442/646, 68.4%) was as expected (see Figure 1 for the CONSORT [Consolidated Standards of Reporting Trials] flow). A number of participants (*Parent Positive*: 30/320, 9.4%; FAU: 13/326, 4%) were unintentionally contacted in addition between T1 and T2 data collection because of a temporary Qualtrics syntax error; these data were discarded.

### Sample Characteristics

Baseline characteristics are shown in Tables 1 and 2. The mean child age was 7.45 (SD 1.67) years, with similar proportions of male and female participants; 86.4% (558/646) of parents identified their child as of White ethnicity, 78.8% (509/646) lived in a 2-adult household, and 81.9% (529/646) lived in 1- or 2-child households. A total of 63.9% (413/646) of parents were employed. In total, 4.8% (31/646) had no access to outdoor spaces. A total of 8% (52/646) of children were not attending school in person. The largest between-arm imbalances were observed in work status and living in a household with <4 people.

**Table 1:** Demographic comparability^a^ of randomized arms at baseline (n=646).

		By randomized arm			Overall
		FAU^b^ arm (n=326)	*Parent Positive* arm (n=320)		
Child age (years), mean (SD)		7.52 (1.65)	7.38 (1.68)	7.45 (1.67)	
**Gender, n (%)**					
	Male	172 (52.8)	158 (49.4)	330 (51.1)	
	Female	152 (46.6)	161 (50.3)	313 (48.5)	
	Prefer not to say	2 (0.6)	1 (0.3)	3 (0.5)	
**Ethnicity, n (%)**					
	Asian or Asian British: Indian, Pakistani, Bangladeshi, or other	11 (3.3)	12 (3.8)	23 (3.6)	
	Black or Black British: Caribbean, African, or other	4 (1.2)	2 (0.6)	6 (0.9)	
	Chinese or Chinese British	0 (0)	1 (0.3)	1 (0.2)	
	Middle Eastern or Middle Eastern British: Arab, Turkish, or other	1 (0.3)	1 (0.3)	2 (0.3)	
	White British, Irish, or other	288 (88.3)	270 (84.4)	558 (86.4)	
	Mixed-race White and Black or White and Black British	9 (2.8)	12 (3.8)	21 (3.3)	
	Mixed-race other	9 (2.8)	16 (5)	25 (3.9)	
	Other ethnic group	2 (0.6)	3 (0.9)	5 (0.8)	
	Prefer not to say	2 (0.6)	3 (0.9)	5 (0.8)	
**Parents’ ethnicity, n (%)**					
	Asian or Asian British: Indian, Pakistani, Bangladeshi, or other	11 (3.3)	12 (3.8)	23 (3.6)	
	Black or Black British: Caribbean, African, or other	4 (1.2)	2 (0.6)	6 (0.9)	
	Chinese or Chinese British	1 (0.3)	1 (0.3)	2 (0.3)	
	Middle Eastern or Middle Eastern British: Arab, Turkish, or other	0 (0)	2 (0.6)	2 (0.3)	
	White British, Irish, or other	299 (91.7)	285 (89.1)	584 (90.4)	
	Mixed-race White and Black or White and Black British	4 (1.2)	5 (1.6)	9 (1.4)	
	Mixed-race other	3 (0.9)	6 (1.9)	9 (1.4)	
	Other ethnic group	2 (0.6)	4 (1.3)	6 (0.9)	
	Prefer not to say	2 (0.6)	3 (0.9)	5 (0.8)	
**SES^c^ measured by household income (£; £1=US $1.26), n (%)**					
	<16,000 a year or 310 a week	33 (10.1)	26 (8.1)	59 (9.1)	
	16,000-29,999 a year or 310-569 a week	40 (12.3)	34 (10.6)	74 (11.5)	
	30,000-59,999 a year or 580-1149 a week	97 (29.8)	84 (26.3)	181 (28)	
	60,000-89,999 a year or 1500-1729 a week	77 (23.6)	86 (26.9)	163 (25.2)	
	90,000-119,999 a year or 1730-2299 a week	33 (10.1)	39 (12.2)	72 (11.6)	
	>120,000 a year or 2300 a week	29 (8.9)	32 (10)	61 (9.4)	
	Prefer not to say	16 (4.9)	16 (5)	32 (5)	
	Missing	1 (0.3)	3 (0.9)	4 (0.6)	

^a^If there is no *missing* row, then the data were complete.

^b^FAU: follow-up as usual.

^c^SES: socioeconomic status.

**Table 2:** Household composition and COVID-19 variables^a^ at T1 by randomized arm at baseline (n=646).

		By randomized arm			Overall, n (%)
		FAU^b^ arm (n=326), n (%)	*Parent Positive* arm (n=320), n (%)		
**Number of other adults in household**					
	0 (single-parent household)	43 (13.2)	38 (11.9)	81 (12.5)	
	1	257 (78.8)	252 (78.8)	509 (78.8)	
	2	18 (5.5)	22 (6.9)	40 (6.2)	
	3	4 (1.2)	3 (0.9)	7 (1.1)	
	4	3 (0.9)	3 (0.9)	6 (0.9)	
	Non–single-parent household (1-4 other adults)	282 (86.5)	280 (87.5)	562 (87)	
	Missing	1 (0.3)	2 (0.6)	3 (0.5)	
**Number of other children in household**					
	0	90 (27.6)	79 (24.7)	169 (26.2)	
	1	179 (54.9)	181 (56.6)	360 (55.7)	
	2	49 (15)	49 (15.3)	98 (15.2)	
	3	7 (2.1)	6 (1.9)	13 (2)	
	4	0 (0)	4 (1.3)	4 (0.6)	
	5	1 (0.3)	1 (0.3)	2 (0.3)	
**Total number of people in the household**					
	2	17 (5.2)	13 (4.1)	30 (4.6)	
	3	90 (27.6)	77 (24.1)	167 (25.9)	
	4	152 (46.6)	164 (51.3)	316 (48.9)	
	5	49 (15)	44 (13.8)	93 (14.4)	
	6	10 (3.1)	8 (2.5)	18 (2.8)	
	7	4 (1.2)	8 (2.5)	12 (1.9)	
	8	3 (0.9)	4 (1.3)	7 (1.1)	
	Missing	1 (0.3)	2 (0.6)	3 (0.5)	
**Number of rooms**					
	2	15 (4.6)	17 (5.3)	32 (5)	
	3	19 (5.8)	23 (7.2)	42 (6.5)	
	4	43 (13.2)	35 (10.9)	78 (12.1)	
	5	77 (23.6)	55 (17.2)	132 (20.4)	
	6	50 (15.3)	85 (26.6)	135 (20.9)	
	7	60 (18.4)	53 (16.6)	113 (17.5)	
	8	40 (12.3)	36 (11.3)	76 (11.8)	
	9	16 (4.9)	9 (2.8)	25 (3.9)	
	10	5 (1.5)	3 (0.9)	8 (1.2)	
	11	1 (0.3)	2 (0.6)	3 (0.5)	
	12	0 (0)	2 (0.6)	2 (0.3)	
**Overcrowding index**					
	No	289 (88.7)	278 (86.9)	567 (87.8)	
	Yes	36 (11)	40 (12.5)	76 (11.8)	
	Missing	1 (0.3)	2 (0.6)	3 (0.5)	
**Access to outside space**					
	No	19 (5.8)	12 (3.8)	31 (4.8)	
	Yes	307 (94.2)	308 (96.3)	615 (95.2)	
**Has parent had COVID-19?**					
	Yes, diagnosed and recovered	26 (8)	22 (6.9)	48 (7.4)	
	Yes, diagnosed and still ill	3 (0.9)	0 (0)	3 (0.5)	
	Suspected and recovered	47 (14.4)	41 (12.8)	88 (13.6)	
	Suspected and still ill	1 (0.3)	4 (1.3)	5 (0.8)	
	No	249 (76.4)	253 (79.1)	502 (77.7)	
**Has child had COVID-19?**					
	Yes, diagnosed and recovered	11 (3.4)	5 (1.6)	16 (2.5)	
	Yes, diagnosed and still ill	1 (0.3)	0 (0)	1 (0.2)	
	Suspected and recovered	35 (10.7)	33 (10.3)	68 (10.5)	
	Suspected and still ill	2 (0.6)	1 (0.3)	3 (0.5)	
	No	277 (85)	281 (87.8)	558 (86.4)	
**Isolation status**					
	I am living my life as normal	78 (23.9)	80 (25)	158 (24.5)	
	I am not self-isolating, but I have cut down on my usual activities as a precaution or am social distancing	241 (73.9)	230 (71.9)	471 (72.9)	
	I am self-isolating	7 (2.1)	10 (3.1)	17 (2.6)	
**Local lockdown in last month**					
	Yes	119 (36.5)	110 (34.4)	229 (35.4)	
	No	207 (63.5)	210 (65.6)	417 (64.6)	
**Working from home status**					
	At home	89 (27.3)	114 (35.6)	203 (31.4)	
	Out of the home	63 (19.3)	44 (13.8)	107 (16.6)	
	Both	54 (16.6)	49 (15.3)	103 (15.9)	
	Not working	111 (34)	96 (30)	207 (32)	
	Missing	9 (2.8)	17 (5.3)	26 (4)	
**Physically attended school in the last week**					
	Yes	297 (91.1)	295 (92.2)	592 (92.1)	
	No	28 (8.6)	24 (7.5)	52 (8)	
	Missing	1 (0.3)	1 (0.3)	2 (0.3)	

^a^If there is no *missing* row, then the data were complete.

^b^FAU: follow-up as usual.

### Parent Positive Use

By T2 and T3, a total of 81.9% (262/320) and 82.8% (265/320) of participants in the *Parent Positive* arm had accessed the app (see Tables 3 and 4), respectively, with a higher proportion accessing the first few boosters as compared with the last few (eg, 153/262, 58.4% accessing booster 1 vs 45/262, 17.2% accessing booster 5 at T2; Tables S2 and S3 in Multimedia Appendix 1). Time spent in the *Parenting Boosters* zone was highly skewed, with a small number of participants having high use. Participants spent the most time on booster 1 (“Staying positive and motivated”) and the least time on booster 5 (“Promoting better behaviour”; Tables S2 and S3 in Multimedia Appendix 1). A total of 4.9% (13/265) of participants published posts by T3. The number that commented increased from 13.2% (35/262) by T2 to 17.7% (47/265) by T3. The median usefulness scores in those who provided data were 3.0 (IQR 2.3-4.0) at both T2 (IQR 2.3-4.0; 126/320, 39.4%) and T3 (IQR 2.0-3.7; 139/320, 43.4%).

**Table 3:** App use summary statistics between T1 and T2 in all participants randomized to *Parent Positive* and only in those who used the app.

		*Parent Positive* arm (n=320)	*Parent Positive* user subgroup (n=262)
**Any app use^a^, n (%)**			
	No use	41 (12.8)	N/A^b^
	Some use	262 (81.9)	N/A
	Missing^c^	17 (5.3)	N/A
**Number of times the app was accessed**			
	Value, mean (SD)	3.00 (4.79)	3.47 (4.99)
	Value, median (IQR)	2 (1-3)	2 (1-3)
	Value, range	0-47	1-47
	Missing, n (%)	17 (5.3)	0 (0)
Viewing any boosters, n (%)		203 (63.4)	203 (77.5)
**Number of boosters started**			
	Value, mean (SD)	2.15 (2.36)	2.49 (2.37)
	Value, median (IQR)	1 (0-3)	2 (1-4)
	Value, range	0-8	0-8
	Missing, n (%)	19 (5.9)	2 (0.8)
**Time spent across all boosters (minutes)**			
	Value, mean (SD)	48 (190)	56 (203)
	Value, trimmed mean^d^ (SD)	6 (10)	8 (12)
	Value, median (IQR)	2 (0-13)	4 (0-16)
	Value, range	0-1541	0-1541
	Missing, n (%)	19 (5.9)	2 (0.8)
Publishing at least 1 post in the *Parenting Exchange* zone, n (%)		N/A	12 (4.6)
**Number of published posts**			
	Value, mean (SD)	N/A	0.06 (0.28)
	Value, median (IQR)	N/A	0 (0-0)
	Value, range	N/A	0-2
	Missing, n (%)	N/A	0 (0)
Publishing at least 1 comment in the *Parenting Exchange* zone, n (%)		N/A	35 (13.4)
**Number of published comments**			
	Value, mean (SD)	N/A	0.57 (2.26)
	Value, median (IQR)	N/A	0 (0-0)
	Value, range	N/A	0-21
	Missing, n (%)	N/A	0 (0)

^a^App use is defined as whether participants had any recorded sessions using the app. Where app use metrics were summarized for the whole *Parent Positive* arm, values of 0 use were used for *Parent Positive* participants who had never used the app.

^b^N/A: not applicable.

^c^Use data were missing for 17 participants because of an issue with retrieving information from the app use database.

^d^Trimmed mean was the mean of the set of values with extreme outliers (values of >3 IQRs above the upper quartile) removed.

**Table 4:** App use summary statistics between T1 and T3 in all participants randomized to Parent Positive and only in those who used the app.

		*Parent Positive* arm (n=320)	*Parent Positive* user subgroup (n=265)
**Any app use, n (%)**			
	No use	36 (11.3)	N/A^a^
	Some use	265 (82.8)	N/A
	Missing	19 (5.9)	N/A
**Number of times the app was accessed**			
	Value, mean (SD)	4.08 (6.55)	4.63 (6.79)
	Value, median (IQR)	2 (1-4)	3 (1-5)
	Value, range	0-52	1-52
	Missing, n (%)	19 (5.9)	0 (0.0)
Viewing any boosters, n (%)		213 (66.6)	213 (80.4)
**Number of boosters started**			
	Value, mean (SD)	2.52 (2.54)	2.86 (2.52)
	Value, median (IQR)	2 (0-4)	2 (1-4)
	Value, range	0-8	0-8
	Missing, n (%)	21 (6.6)	2 (0.8)
**Time spent across all boosters (minutes)**			
	Value, mean (SD)	65 (244)	74 (259)
	Value, trimmed mean (SD)	8 (13)	10 (13)
	Value, median (IQR)	4 (0-18)	6 (1-20)
	Value, range	0-2254	0-2254
	Missing, n (%)	21 (6.6)	2 (0.8)
Publishing at least 1 post in the *Parenting Exchange* zone, n (%)		N/A	13 (4.9)
**Number of published posts**			
	Value, mean (SD)	N/A	0.06 (0.31)
	Value, median (IQR)	N/A	0 (0-0)
	Value, range	N/A	0-3
	Missing, n (%)	N/A	0 (0)
Publishing at least 1 comment in the *Parenting Exchange* zone, n (%)		N/A	47 (17.7)
**Number of published comments**			
	Value, mean (SD)	N/A	0.70 (2.66)
	Value, median (IQR)	N/A	0 (0-0)
	Value, range	N/A	0-30
	Missing, n (%)	N/A	0 (0)

^a^N/A: not applicable.

### Clinical Outcomes

The primary analysis model included 79.3% (512/646) of participants with at least 1 T2 SDQ conduct score (236/320, 73.8% in *Parent Positive* and 276/326, 84.7% in FAU). Baseline household income and the number of adults in the household variables predicted missing outcome data and were included in all the models.

**Figure 2:**
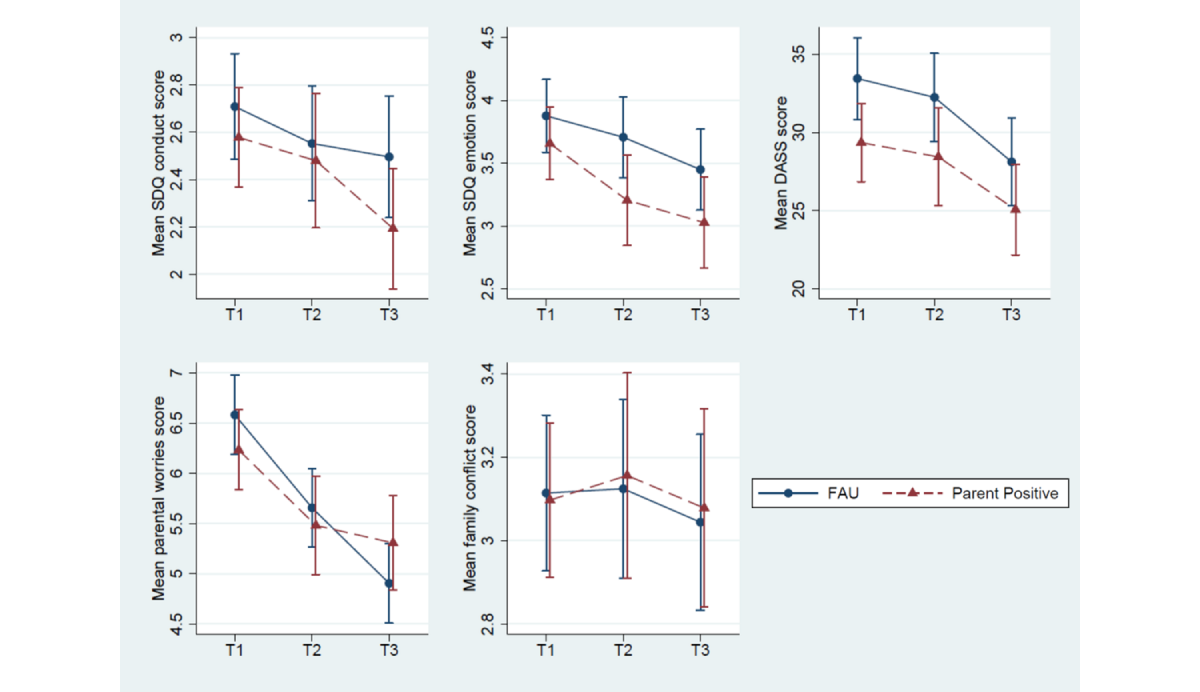
Unadjusted outcome means (−95% CIs to +95% CIs) at baseline (T1) and the 2 follow-up time points (T2=1 month and T3=2 months). DASS: Depression, Anxiety, and Stress Scale; FAU: follow-up as usual; SDQ: Strengths and Difficulties Questionnaire.

**Figure 3:**
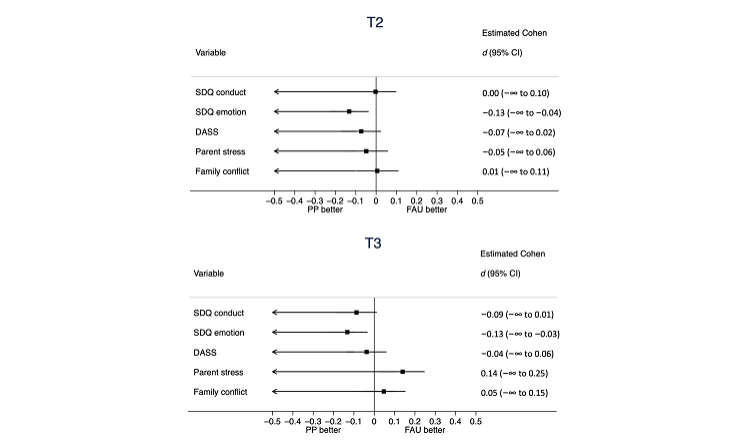
Adjusted standardized mean outcome differences between randomized arms at the 2 follow-up time points (T2 and T3) with 1-sided 95% CIs. DASS: Depression, Anxiety, and Stress Scale; FAU: follow-up as usual; SDQ: Strengths and Difficulties Questionnaire.

Figure 2 shows unadjusted mean profile plots of the outcomes. Table 5 shows summary statistics and between-arm differences (other SDQ subscales are summarized in Table S3 in Multimedia Appendix 1), with standardized group differences displayed in Table 5 and Figure 3. Baseline values for all 5 outcome measures were higher in the *Parent Positive* arm. Conduct problems (SDQ) were no lower in *Parent Positive* than in FAU at either T2 (primary outcome; Cohen *d*=−0.01, 1-sided 95% CI −∞ to 0.20) or T3 (Cohen *d*=−0.09, 1-sided 95% CI −∞ to 0.03). In contrast, mean emotional symptoms (SDQ) were statistically significantly lower in *Parent Positive* than in FAU at both T2 (Cohen *d*=−0.13, 1-sided 95% CI −∞to -0.10) and T3 (Cohen *d*=−0.13, 1-sided 95% CI −∞ to -0.09). There was no evidence of less parent psychological distress (DASS), parental child-related worries, or family conflict in *Parent Positive* versus FAU at either time point. Although our preregistered analysis focused on 1-sided tests, the 2-sided *P* values (Table S4 and Figures S1 and S2 in Multimedia Appendix 1) showed that T3 parental worries were significantly higher in the *Parent Positive* arm.

**Table 5:** Outcome variables summarized at T1, T2, and T3 by randomized arm and estimated mean treatment differences at T2 and T3 (n=646).

	*Parent Positive* arm (n=320)		FAU^a^ arm (n=326)		Overall		Mean difference^b^	Standardized effect	1-sided 95% CI	1-sided *P* value
	Complete cases, n (%)	Mean (SD)	Complete cases, n (%)	Mean (SD)	Complete cases, n (%)	Mean (SD)				
SDQ^c^—conduct at baseline	320 (100)	2.58 (1.93)	326 (100)	2.71 (2.05)	646 (100)	2.64 (1.99)	N/A^d^	N/A	N/A	N/A
SDQ—conduct at 1 month	200 (62.5)	2.48 (2.04)	266 (81.6)	2.55 (2.01)	466 (72.1)	N/A	−0.01	−0.003	∞ to 0.20	.48
SDQ—conduct at 1 month CACE^e^	N/A	N/A	N/A	N/A	N/A	N/A	−0.02	N/A	−∞ to 0.28	N/A
SDQ—conduct at 2 months	186 (58.1)	2.19 (1.77)	256 (78.5)	2.50 (2.10)	442 (68.4)	N/A	−0.17	−0.09	−∞ to 0.03	.08
SDQ—conduct at 2 months CACE	N/A	N/A	N/A	N/A	N/A	N/A	−0.28	N/A	−∞ to 0.02	N/A
SDQ—emotion at baseline	320 (100)	3.66 (2.63)	326 (100)	3.88 (2.66)	646 (100)	3.77 (2.65)	N/A	N/A	N/A	N/A
SDQ—emotion at 1 month	200 (62.5)	3.21 (2.59)	266 (81.6)	3.71 (2.66)	466 (72.1)	N/A	−0.35	−0.13	−∞ to −0.10	.01
SDQ—emotion at 1 month CACE	N/A	N/A	N/A	N/A	N/A	N/A	−0.46	N/A	−∞ to −0.08	N/A
SDQ—emotion at 2 months	186 (58.1)	3.03 (2.52)	256 (78.5)	3.45 (2.62)	442 (68.4)	N/A	−0.35	−0.13	−∞ to −0.09	.02
SDQ—emotion at 2 months CACE	N/A	N/A	N/A	N/A	N/A	N/A	−0.40	N/A	−∞ to 0.004	N/A
DASS^f^ at baseline	320 (100)	29.4 (22.8)	326 (100)	33.4 (24.1)	646 (100)	31.4 (23.5)	N/A	N/A	N/A	N/A
DASS at 1 month	199 (62.2)	28.4 (22.4)	264 (81)	32.2 (23.4)	463 (71.7)	N/A	−1.71	−0.07	−∞ to 0.56	.11
DASS at 2 months	185 (57.8)	25.1 (20.1)	256 (78.5)	28.1 (22.8)	441 (68.3)	N/A	−0.88	−0.04	−∞ to 1.42	.26
Parental worries at baseline	319 (99.7)	6.23 (3.64)	326 (100)	6.58 (3.64)	645 (99.8)	6.41 (3.64)	N/A	N/A	N/A	N/A
Parental worries at 1 month	199 (62.2)	5.48 (3.52)	266 (81.6)	5.66 (3.26)	465 (72)	N/A	−0.17	−0.05	−∞ to 0.21	.23
Parental worries at 2 months	185 (57.8)	5.31 (3.25)	256 (78.5)	4.91 (3.23)	441 (68.3)	N/A	0.51	0.14	−∞ to 0.90	.98
Family conflict at baseline	309 (96.6)	3.10 (1.72)	314 (96.3)	3.11 (1.75)	623 (96.4)	3.11 (1.73)	N/A	N/A	N/A	N/A
Family conflict at 1 month	192 (60)	3.16 (1.74)	257 (78.8)	3.12 (1.75)	449 (69.5)	N/A	0.01	0.01	−∞ to 0.19	.54
Family conflict at 2 months	179 (55.9)	3.08 (1.62)	248 (76.1)	3.04 (1.69)	427 (66.1)	N/A	0.08	0.05	−∞ to 0.26	.77

^a^FAU: follow-up as usual.

^b^Estimates of mean differences (*Parent Positive* minus FAU; therefore, negative differences imply that *Parent Positive* is better than FAU, ie, all measures are higher score=worse) were derived from linear mixed-effects models of untransformed measures at both follow-up time points and using treatment arm, categorical time point, treatment-time interaction, baseline measure of the outcome, age, gender, household income (<£30,000 per year vs ≥£30,000 per year), and number of adults in the household (1 vs 2 vs ≥3). Robust standardized effects were modeled in all final analyses.

^c^SDQ: Strengths and Difficulties Questionnaire.

^d^N/A: not applicable.

^e^CACE: complier average causal effect estimate.

^f^DASS: Depression, Anxiety, and Stress Scale.

### Service Use, Costs, and Economic Outcomes

Service use and cost data are presented in Tables S5-S7 in Multimedia Appendix 1. Mean service use was low in both arms for all services. The cost of *Parent Positive* use within the trial was estimated to be £21 per family (Table S1 in Multimedia Appendix 1). Observed total health and social care costs per participant were higher in the *Parent Positive* arm because of higher mean costs of hospital services for mental health, physical illnesses, and injuries (mean £241, SD £927 in *Parent Positive* versus mean £196, SD £491 in FAU at T2; mean £136, SD £775 in *Parent Positive* versus mean £83, SD £269 in FAU at T3; Table S7 in Multimedia Appendix 1). This difference was primarily due to 4 outliers with extremely high costs (99th percentile): 3 (75%) in the *Parent Positive* arm (with total T2 costs of £3141.25, £3474.25, and £11,381.11) and 1 (25%) in the FAU arm (with total T2 costs of £2836). These outliers were excluded from the economic analysis (see Multimedia Appendix 1 for the analysis, including outliers).

Excluding influential outliers, complete case mean costs were lower in the *Parent Positive* arm than in the FAU arm (mean £144, SD £326 vs mean £176, SD £445 at T2 and mean £44, SD £104 vs mean £73, SD £445 at T3, respectively; Table S7 in Multimedia Appendix 1). Complete case CHU9D health state scores increased only very slightly over time in both arms, and between-arm differences in QALYs were negligible (mean 0.0003, SD 0.0019 in *Parent Positive* vs mean 0.0002, SD 0.0016 in FAU at T2; mean 0.0006, SD 0.0028 in *Parent Positive* vs mean 0.0003, SD 0.0019 in FAU at T3; Table S8 in Multimedia Appendix 1).

### Economic Analysis

The results of the primary, sensitivity, and secondary economic analyses are summarized in Table S9 in Multimedia Appendix 1. Excluding outliers, the *Parent Positive* arm achieved slightly higher QALYs than the FAU arm (adjusted mean difference=0.000132; standard error= SE 0.000193, 95% CI −0.000219 to 0.000536; *P*=.41) at a lower cost per participant (adjusted mean difference=−£18.89 [US $23.32]; standard error= SE £34.47 [US $42.55], 95% CI −£88.35 to £46.79 [US $109.07 to $57.76]; *P*=.55; see Figure S3 in Multimedia Appendix 1). The probability of *Parent Positive* being cost-effective compared with FAU was 72% and 74% at the National Institute for Health and Care Excellence willingness-to-pay thresholds of £20,000 and £30,000 per QALY, respectively (Figure S4 in Multimedia Appendix 1). Results were similar in the sensitivity analyses using the SDQ conduct subscale and including multiple imputation of missing data, as well as for the secondary analysis carried out at T3 (Table S9 in Multimedia Appendix 1). However, the results were sensitive to outliers, with FAU having a higher probability of being cost-effective than *Parent Positive* for all economic analyses (primary, sensitivity, and secondary) when outliers were included as a result of cost differences in favor of FAU (Table S9 in Multimedia Appendix 1).

### Reported AEs

There were 927 AEs across 53.1% (343/646) of the participants, with 16 (1.7%) serious AEs, none of which were deemed related to trial participation (see Table 6).

There was no evidence of moderation of the *Parent Positive* versus FAU intervention effect on T2 and T3 conduct problems by level of baseline conduct problems (*P*=.17).

**Table 6:** Summary of adverse events (n=646).

		Parent Positive arm (n=320)			FAU^a^ arm (n=326)			Overall	
		Events, N	Participants, n (%)	Events, N		Participants, n (%)	Events, N		Participants, n (%)
All adverse events		387	155 (48.4)	540		188 (57.7)	927		343 (53.1)
**Seriousness level^b^**									
	No seriousness categories applied	380	152 (47.5)	531		185 (56.7)	911		337 (52.2)
	Required hospitalization or prolongation of existing hospitalization	7	7 (2.2)	8		8 (2.5)	15		15 (2.3)
	Persistent or substantial disability or incapacity	0	0 (0)	1		1 (0.3)	1		1 (0.2)
**Severity grade^b^**									
	Mild	306	143 (44.7)	430		174 (53.4)	736		317 (49.1)
	Moderate	68	52 (16.3)	90		63 (19.3)	158		115 (17.8)
	Severe	13	12 (3.8)	20		17 (5.2)	33		29 (4.5)

^a^FAU: follow-up as usual.

^b^Note that some participants had adverse events in more than one seriousness or severity category; therefore, the column totals may not add up to the total in the top row.

## Discussion

### Principal Findings

*Parent Positive* was developed as a universal public health intervention to reverse the increase in children’s behavioral and emotional problems that occurred in the United Kingdom during the pandemic. In SPARKLE, we tested its effects on children’s conduct and emotional problems as well as on parents’ psychological distress, child-related worries, and family conflict. We found mixed results.

On the basis of the very high and rapid rate of recruitment into the trial (646 participants in just over 2 months), high levels of retention, and relatively high numbers accessing the app compared with those in trials of similar mental health apps [36], there seemed to be a strong demand for the intervention. This was especially striking when one considers that this was a general population sample unselected on levels of conduct and emotional problems—although the wider Co-SPACE cohort reported fewer conduct problems than those who took part in SPARKLE [5]. Intervention-related AEs were very rare in the study, and the high number of AEs reported in both arms was likely to be due to how these were ascertained in SPARKLE as all parent-reported familial medical and nonmedical AEs were recorded.

*Parent Positive* did not produce significant reductions in parent-reported children conduct problems after either 1 or 2 months of access. Conduct problems were selected as our primary outcome as one of the main purposes of the app was to give parents advice on common parenting challenges and managing difficult behaviors. Although not statistically significant, the difference in conduct problems between the 2 arms in favor of *Parent Positive* increased substantially by T3. This was in the context of restrictions easing, schools being open again, and reductions in conduct problems at the general population level being reported [5]. It would be interesting in future trials to extend the intervention period to ≥3 months on the grounds that it may take time for skills to embed and begin to show benefits [37]. Given *Parent Positive*’s emphasis on creating positive environments and experiences for parents and children, we also predicted reductions in children’s emotional problems. More specifically, we expected *Parent Positive* to reduce child worry and raise mood by encouraging the use of praise and other positive strategies, and there is evidence that parent training improves child internalizing problems [38]. Consistent with these predictions, *Parent Positive* significantly reduced parent-reported child emotional problems at T2 and T3. The complier average causal effect analysis demonstrated that these effects were stronger when the analysis was restricted to those meeting our criteria for sufficient app use (Multimedia Appendix 1).

Although we were unable to model the specific effects of individual boosters on outcomes statistically, an inspection of their use provides some interesting insights. For instance, engagement in the boosters most likely to be associated with reductions in conduct problems (eg, “Promoting better behaviour” and “Using sanctions carefully”) was limited and may account for some lack of effect on conduct problems. In contrast, the boosters focusing on parental and more general family-related processes—“Keeping positive and motivated,” “Keeping calm when your child acts up,” and “Making sure everyone knows what’s expected of them”—were the most often accessed. It is possible that these boosters improved the general emotional atmosphere in the family and so were responsible for the significant drop in emotional problems in the *Parent Positive* arm.

It is important to note that we anticipated that *Parent Positive* would reduce parenting-related stress both directly because of the app’s focus on parental well-being and self-care and indirectly because of any beneficial effects *Parent Positive* had on children’s well-being and the improvements in parenting confidence that we expected this to bring. In contrast, there was a relative preponderance of parental child-related worries in the *Parent Positive* arm compared with the FAU arm by T3. It is unclear what the source of this effect might be and why it was absent at T2. For example, it may be an unintended consequence of some aspects of the *Parent Positive* processes or content. Possibilities include challenges related to changing parenting practices and the worry associated with greater awareness of limitations in one’s parenting skills in light of increased parenting knowledge and interactions with other parents and experts. It is also possible that the pattern of app use was different in the first and second months of the trial, explaining why this effect emerged between T2 and T3. However, inspection of Tables S2 and S5 in Multimedia Appendix 1 does not suggest a significant change in app use between these periods.

However, we cannot dismiss the possibility that this is a chance finding or that a different pattern with longer follow-up may emerge—the fact that it was absent at T2 may support this view. It could also be due to the selective retention of parents in the *Parent Positive* arm with more child-related worries leading the parent to provide more outcome data at T2, although this is not wholly consistent with the intervention-related reductions in parents’ ratings of children’s emotional problems.

What are the practical implications of our findings? The reduction in emotional problems in those allocated to *Parent Positive* of 0.13 SD (at both T2 and T3) would be considered a small effect in a trial of treatment effectiveness in a clinical population, and in such a context, might be dismissed as being of little practical importance. However, we can apply a different standard to community-based mental health interventions such as *Parent Positive*, where small effects observed at the individual level can make a substantial contribution to the mental health of a whole community [39]; small effects aggregated across many individuals can combine to make a substantial effect overall. In this way, based on these findings, *Parent Positive,* if implemented across the general UK population, has the potential to make a substantial contribution to reducing emotional problems. However, caution is warranted when drawing such a conclusion based on what is a relatively small sample for a public health trial. Nevertheless, the magnitude of improvement is consistent with findings of 0.07 to 0.16 SD from a meta-analysis of more intensive but universally targeted interventions that aim to reduce young people’s emotional and behavioral problems [40]. Our finding is particularly striking as many previous interventions studied have been reliant on face-to-face delivery by a trained professional [40]. Given that such interventions are likely to be more logistically challenging and costly to implement than *Parent Positive*, this highlights the potential health economic benefits of digital parenting approaches and their substantial public health value given their accessibility and universal availability. If offered to families when their children are young and emotional problems have not yet developed or are less entrenched, there is a possibility that *Parent Positive* could have positive effects on developmental trajectories at a large scale.

Economic analysis was heavily influenced by the extremely high health care–related costs of 4 individuals, the 3 highest of whom were randomized by chance to *Parent Positive*. This was problematic as service use data were not routinely collected in Co-SPACE and so we were unable to control for baseline service use. We addressed this by removing participants with outlier costs in the 99th percentile. With this, the likelihood of *Parent Positive* being cost-effective compared with FAU increased from between 19% and 20% to between 73% and 74%. On balance, we would argue that *Parent Positive* is likely to be cost-effective when implemented across the general population, but this remains to be tested in larger studies where the effect of outliers may be less important or baseline service use data are obtained. In addition, the CHU9D algorithm used to generate QALYs from SDQ scores was based on a clinical child sample. It is unknown whether the algorithm is sufficient for picking up on changes in QALYs in nonclinical child general population samples.

More generally with regard to methodology, the SPARKLE RCT highlights the feasibility and potential value of rapid deployment and evaluation of digital interventions as trials within general population cohorts with a regular collection of outcome data. This was especially important in light of the pandemic-related restrictions. We built the app, recruited 646 participants, and completed a 2-month data collection period all in 11 months.

### Limitations

The RCT was conducted during spring 2021 and summer 2021, when pandemic-related restrictions were beginning to ease (ie, children were not homeschooled en masse, and restrictions on social contacts had relaxed). In this sense, we are unable to generalize our findings to more restrictive lockdown periods or to nonrestricted contexts, and parents of children in Co-SPACE reported a drop in conduct problems when restrictions eased [5]. However, during the RCT, there was continued concern and uncertainty about the course of the pandemic and its continued impact. Second, we had differential dropout between arms, with higher dropout in the *Parent Positive* arm. This is possibly due to the families in the FAU arm waiting for posttrial access to *Parent Positive* and, therefore, being more engaged in the data collection process. Third, the Co-SPACE cohort and, therefore, the SPARKLE subsample are underrepresentative of ethnic minority groups and low-income families. The data in Table 1 indicate that the sample had a somewhat higher proportion of White British participants (558/646, 86.4% of children and 584/646, 90.4% of parents) than the UK population as a whole (82%) and were less likely to be living on very low incomes (59/646, 9.1% on <£16,000 per year [SPARKLE] vs 22% [United Kingdom]) [41]. Finally, the overall number of *Parent Positive* users was too low to establish the real value of the *Parenting Exchange*. The 90-9-1 principle of engagement within web-based communities explains that 90% of users observe but do not engage in web-based exchanges, 9% provide some contribution, and 1% of users generate most of the content [42]. Therefore, in the SPARKLE trial, where the app had <300 users, the level of engagement with the *Parenting Exchange* was unsurprisingly low. Nevertheless, we should not discount the potential value of *Parenting Exchange* for parents who might be seeking social connections and shared understanding within a safe and supportive online community.

### Conclusions

This was the first RCT of a parenting support app designed specifically to support parents in the general population during the COVID-19 pandemic and one of the few examples of a trial within a cohort in our field. It used a rigorous design in a large sample and collected data on a range of outcomes over a 2-month period. *Parent Positive* has potential as a way of reducing young children’s emotional problems in the general population. However, the app was developed quickly to meet the pandemic-related needs of families. We are currently funded to work with parents to codevelop the app further to improve usability; increase engagement; and, ultimately, improve the effects on parent and child outcomes. Research with stakeholders (eg, teachers, general practitioners, and child and adolescent mental health practitioners) will help optimize its value in both clinical and nonclinical settings.

## Appendix

Multimedia Appendix 1 Outcome analysis using complier average causal effect, economic analysis, and CONSORT (Consolidated Standards of Reporting Trials) checklist.

Multimedia Appendix 2 CONSORT-EHEALTH (V 1.6.1) checklist.

## Abbreviations

AE: adverse event
CA-SUS: Child and Adolescent Service Use Schedule
CHU9D: Child Health Utility–9 Dimensions
CONSORT: Consolidated Standards of Reporting Trials
Co-SPACE: COVID-19: Supporting Parents, Adolescents and Children during Epidemics
DASS: Depression, Anxiety, and Stress Scale
EPEC: Empowering Parents Empowering Communities
FAU: follow-up as usual
LMM: linear mixed model
QALY: quality-adjusted life year
RCT: randomized controlled trial
SAP: Statistical Analysis Plan
SDQ: Strengths and Difficulties Questionnaire
SPARKLE: Supporting Parents And Kids Through Lockdown Experiences
STEPS: Structured E-Parenting Support app
T1: before randomization
T2: 1 month after randomization
T3: 2 months after randomization
TSC: Trial Steering Committee
