# Supplementary material for: The Effectiveness and Cost-Effectiveness of a Universal Digital Parenting Intervention Designed and Implemented During the COVID-19 Pandemic: Evidence From a Rapid-Implementation Randomized Controlled Trial Within a Cohort
Source: J Med Internet Res. 2023 Jul 27;25:e44079. doi: 10.2196/44079 (PMC10415938; doi:10.2196/44079)
Supplement: Multimedia Appendix 1 [file jmir_v25i1e44079_app1.docx]

**Title:** The effectiveness of a universal digital parenting intervention designed and implemented during the COVID-19 pandemic: Evidence from a rapid implementation randomised controlled trial within a cohort.

**Online Supplement**

**Outcome analysis using complier average causal effect**

A more principled per protocol-type complier average causal effect (CACE) analysis providing *Parent Positive* vs FAU estimates[20,21] in the subset of participants using the app between T1 and T2 (defined as those spending 45 seconds or more in any one of the boosters) were obtained using panel generalised two stages least squares instrumental variable effects models (Stata command: xtivreg) with the random effects estimator and robust standard errors [22], the same covariates as for the main ITT model, and intervention group and intervention group by time interaction terms instrumenting the app usage and app usage by time terms (the latter used to calculate the effect at T3). These estimates were obtained for both conduct problems and emotional symptoms outcomes.

Under the CACE estimation app usage definition, 178 (56%) of those randomised to *Parent Positive* used the app. We note there were 19 individuals missing app usage data – therefore the CACE estimates are not directly comparable to the main ITT effect estimates. However, the ITT effects were similar in the subset with complete app usage data (data not shown). The *Parent Positive* versus FAU CACE estimates in the subset using the app were similar to the ITT estimates (the point estimates in each case are within the CI for the other, Table 3). As for the ITT estimates, there was little evidence of a difference between *Parent Positive* and FAU in conduct problems at T2 (unstandardised estimate, -0.02 one sided 95%*CI* -∞, 0.28) or T3 (-0.28 one sided 95%*CI* -∞, 0.02), but evidence of lower emotional problems in the *Parent Positiv*e group at both T2 (-0.46, one sided 95%*CI* -∞ to -0.08) and T3 (-0.40, one sided 95%*CI* -∞ to -0.004).

**Table S1: Unit Costs of Health and Social Care Services^1^**

| Item | Source | Unit cost (2020/2021) |
| --- | --- | --- |
| Intervention^2^ | | |
| App development | Trial budget | £10.09 per parent |
| App support & maintenance | Trial budget | £1.55 per parent |
| Expert time costs | Trial Expert Time Use Records | £9.61 per parent |
| Hospital services | | |
| Inpatient mental health | NHS reference costs for children’s health services (Jones and Burns 2021) | £938 per contact |
| Inpatient paediatrics >=5 days | NHS reference costs for children’s health services (Jones and Burns 2021) | £4930 per episode |
| Inpatient general <5 days | NHS reference costs (Jones and Burns 2021) | £827 per episode |
| Outpatient mental health | Child and Adolescent Mental Health Services Worker (Curtis and Burns 2020) | £240 per contact hour |
| Outpatient paediatrics | NHS reference costs for children’s health services (Jones and Burns 2021) | £224 per attendance |
| Accident & emergency | NHS reference costs (NHS Reference Costs 2019-2020) Accessed 17/02/22 | £194 per attendance |
| Ambulance | NHS reference costs (NHS Reference Costs 2019-2020) Accessed 17/02/22 | £227 per attendance |
| Community health and social care services | | |
| General practitioner | General practitioner (Jones and Burns 2021) | £34 per visit |
| Practice nurse | Nurse (GP practice) (Jones and Burns 2021) | £52 per contact hour^3^ |
| Community nurse | Assumed equivalent to practice nurse | £52 per contact hour^3^ |
| Paediatrician | Assumed equivalent to general practitioner | £34 per visit |
| Mental health worker | Clinical Psychologist Band 7 (Jones and Burns 2021) | £216 per contact hour^4^ |
| Talking therapist | Average of Clinical Psychologist Band 7 (Jones and Burns 2021) and Counselling for children with mental or emotional difficulties (Jones and Burns 2021) | £132 per visit^5^ |
| Speech & language therapist | NHS reference costs for children’s health services (Jones and Burns 2021) | £114 per visit |
| Social worker | Social worker (children’s services) (Jones and Burns 2021) | £260 per contact hour^6^ |
| Self-help lines & apps | Based on cost reported by the Samaritans (Samaritans 2013) | £4.62 per call |
| Educational psychologist | Clinical Psychologist Band 7 (Jones and Burns 2021) | £216 per contact hour^4^ |
| Parent training classes | Parenting interventions for the prevention of persistent conduct disorders (Jones and Burns 2021) | £1,614 per course^7^ |
| Parent support group | Parenting and family support programmes (Early Intervention Foundation 2017) | £99 per programme^8^ |
| Medications | | |
| Fluoxetine 20mg | Drug Tariff (Part VIII) (The NHS Business Services Authority 2021) (accessed 01/12/2021) | Pack of 30 capsules £0.96 |
| Methylphenidate XL 20mg | Drug Tariff (Part VIII) (The NHS Business Services Authority 2021) (accessed 01/12/2021) | Pack of 30 tablets £42.45 |
| Methylphenidate 20mg | Drug Tariff (Part VIII) (The NHS Business Services Authority 2019) (accessed 01/12/2019) | Pack of 30 tablets £10.92 |
| Clonidine 0.25mg | Drug Tariff (Part VIII) (The NHS Business Services Authority 2019) (accessed 01/12/2019) | Pack of 112 tablets £5.01 |

SALT=Speech and Language Therapist; SENCO=Special Education Needs Co-ordinator; ^1^All unit costs are for the financial year 2020/21, uprated where necessary using the GDP deflator (HM Treasury 2021,accessed 1/12/2021). ^2^The cost of the Parenting Exchange and Ask the Expert Sessions was based on salaries plus employer on-costs (superannuation and national insurance), overhead costs (administrative, managerial, capital etc.), and the cost of time for supervision including the cost of providers of supervision. Total costs of expert and paid parent time (£6,210) and app maintenance (one month @ £1,000) were divided by the total number of participants in both groups (n=646) to reflect real world uptake. App development costs of £70,000 were amortised across the total estimated number of users over a projected 24-month effective lifespan (365.25 days x 2 = 730.5 days). The number of users was based on the recruitment rate into the study (9.5 parents per day x 730.5 = 6,939.75 users). ^3^Cost per contact hour calculated by multiplying the cost per working hour by the ratio of direct to indirect time for each type of service reported in (Jones and Burns 2021). ^4^Ratio of direct to indirect time of 1:2.33 reported in (Curtis and Burns 2007). ^5^Average of clinical psychologist and counsellor cost per visit of £132 based on a 50 minute session https://myonlinetherapy.com/cost-to-see-a-psychologist/. ^6^Ratio of direct to indirect time of 1:4.0 reported in (Curtis and Burns 2007) and cost per visit of £390 based on a 90 minute home visit (clinical opinion). ^7^Cost per session of £27 based on a closed group of 10 parents with session attendance of 60% (clinical opinion). ^8^Cost per session of £1 based on 10 open group sessions of 10 parents (clinical opinion).

Table S2a: Total app usage (minutes) between T1 and T2, broken down by booster, in all participants randomised to *Parent Positive*, and only in those who used the app.

|  |  |  |  |
| --- | --- | --- | --- |
|  |  | ***Parent Positive* group  (*n*=320)** | ***Parent Positive* users subgroup  (*n*=262)** |
| **Time spent in booster 1:**  Keeping positive and motivated |  |  |  |
|  | *Mean (SD)* | 16 (120) | 18 (129) |
|  | *Trimmed mean (SD)* | 0.7 (1.3) | 0.9 (1.5) |
|  | *Median (IQR)* | 0.1 (0-1.7) | 0.3 (0-2.1) |
|  | *Range* | 0 - 1333 | 0 - 1333 |
|  | *Missing (%)* | 19 (6%) | 2 (1%) |
|  | *Started (%)* | 153 (48%) | 153 (58%) |
| **Time spent in booster 2:**  Making sure everyone knows what's expected of them |  |  |  |
|  | *Mean (SD)* | 7 (58) | 8 (62) |
|  | *Trimmed mean (SD)* | 0.2 (0.5) | 0.3 (0.6) |
|  | *Median (IQR)* | 0 (0-0.8) | 0 (0-0.9) |
|  | *Range* | 0 - 662 | 0 - 662 |
|  | *Missing (%)* | 17 (5%) | 0 (0%) |
|  | *Started (%)* | 95 (29%) | 95 (35%) |
| **Time spent in booster 3:**  Building your child's self-confidence and trust in you |  |  |  |
|  | *Mean (SD)* | 4 (41) | 4 (44) |
|  | *Trimmed mean (SD)* | 0.3 (0.6) | 0.4 (0.7) |
|  | *Median (IQR)* | 0 (0-0.8) | 0 (0-0.9) |
|  | *Range* | 0 - 707 | 0 - 707 |
|  | *Missing (%)* | 17 (5%) | 0 (0%) |
|  | *Started (%)* | 98 (31%) | 98 (37%) |
| **Time spent in booster 4:**  Getting your child to follow instructions |  |  |  |
|  | *Mean (SD)* | 6 (73) | 7 (79) |
|  | *Trimmed mean (SD)* | 0.2 (0.5) | 0.4 (0.8) |
|  | *Median (IQR)* | 0 (0-0.6) | 0 (0-0.9) |
|  | *Range* | 0 - 1247 | 0 - 1247 |
|  | *Missing (%)* | 17 (5%) | 0 (0%) |
|  | *Started (%)* | 87 (27%) | 87 (33%) |
| **Time spent in booster 5:**  Promoting good behaviour |  |  |  |
|  | *Mean (SD)* | 0 (2) | 0 (2) |
|  | *Trimmed mean (SD)* | 0 (0) | 0 (0) |
|  | *Median (IQR)* | 0 (0-0) | 0 (0-0) |
|  | *Range* | 0 - 19 | 0 - 19 |
|  | *Missing (%)* | 17 (5%) | 0 (0%) |
|  | *Started (%)* | 45 (14%) | 45 (17%) |
| **Time spent in booster 6:**  How to limit conflict |  |  |  |
|  | *Mean (SD)* | 5 (52) | 6 (56) |
|  | *Trimmed mean (SD)* | 0 (0) | 0 (0) |
|  | *Median (IQR)* | 0 (0-0) | 0 (0-0) |
|  | *Range* | 0 - 814 | 0 - 814 |
|  | *Missing (%)* | 17 (5%) | 0 (0%) |
|  | *Started (%)* | 47 (15%) | 47 (18%) |
| **Time spent in booster 7:**  Keeping calm when your kids act up |  |  |  |
|  | *Mean (SD)* | 7 (86) | 9 (93) |
|  | *Trimmed mean (SD)* | 0 (0) | 0.2 (0.5) |
|  | *Median (IQR)* | 0 (0-0) | 0 (0-0.6) |
|  | *Range* | 0 - 1435 | 0 - 1435 |
|  | *Missing (%)* | 17 (5%) | 0 (0%) |
|  | *Started (%)* | 72 (23%) | 72 (27%) |
| **Time spent in booster 8:**  Using sanctions carefully |  |  |  |
|  | *Mean (SD)* | 2 (21) | 3 (23) |
|  | *Trimmed mean (SD)* | 0 (0) | 0 (0) |
|  | *Median (IQR)* | 0 (0-0) | 0 (0-0) |
|  | *Range* | 0 - 353 | 0 - 353 |
|  | *Missing (%)* | 17 (5%) | 0 (0%) |
|  | *Started (%)* | 57 (18%) | 57 (22%) |

Table S2b: Total app usage (minutes) between T1 and T3, broken down by booster, in all participants randomised to *Parent Positive*, and only in those who used the app.

|  |  | ***Parent Positive* group  (*n*=320)** | ***Parent Positive* users subgroup  (*n*=265)** |
| --- | --- | --- | --- |
| **Time spent in booster 1**: Keeping positive and motivated |  |  |  |
|  | *Mean (SD)* | 21 (145) | 24 (154) |
|  | *Trimmed mean (SD)* | 1.0 (1.6) | 1.4 (2.3) |
|  | *Median (IQR)* | 0.3 (0-2.1) | 0.7 (0-2.7) |
|  | *Range* | 0 - 1386 | 0 - 1386 |
|  | *Missing (%)* | 21 (7%) | 2 (1%) |
|  | *Started (%)* | 166 (52%) | 166 (63%) |
| **Time spent in booster 2**: Making sure everyone knows what's expected of them |  |  |  |
|  | *Mean (SD)* | 8 (59) | 9 (63) |
|  | *Trimmed mean (SD)* | 0.3 (0.7) | 0.4 (0.7) |
|  | *Median (IQR)* | 0 (0-0.9) | 0 (0-1.0) |
|  | *Range* | 0 - 662 | 0 - 662 |
|  | *Missing (%)* | 19 (6%) | 0 (0%) |
|  | *Started (%)* | 105 (32%) | 105 (40%) |
| **Time spent in booster 3**: Building your child's self-confidence and trust in you |  |  |  |
|  | *Mean (SD)* | 5 (43) | 6 (46) |
|  | *Trimmed mean (SD)* | 0.5 (0.9) | 0.6 (1.1) |
|  | *Median (IQR)* | 0 (0-1.0) | 0 (0-1.2) |
|  | *Range* | 0 - 707 | 0 - 707 |
|  | *Missing (%)* | 19 (6%) | 0 (0%) |
|  | *Started (%)* | 116 (36%) | 116 (44%) |
| **Time spent in booster 4**: Getting your child to follow instructions |  |  |  |
|  | *Mean (SD)* | 12 (106) | 13 (113) |
|  | *Trimmed mean (SD)* | 0.4 (0.7) | 0.4 (0.8) |
|  | *Median (IQR)* | 0 (0-1.0) | 0 (0-1.1) |
|  | *Range* | 0 - 1325 | 0 - 1325 |
|  | *Missing (%)* | 19 (6%) | 0 (0%) |
|  | *Started (%)* | 101 (32%) | 101 (38%) |
| **Time spent in booster 5**: Promoting good behaviour |  |  |  |
|  | *Mean (SD)* | 1 (6) | 1 (6) |
|  | *Trimmed mean (SD)* | 0 (0) | 0 (0) |
|  | *Median (IQR)* | 0 (0-0) | 0 (0-0) |
|  | *Range* | 0 - 92 | 0 - 92 |
|  | *Missing (%)* | 19 (6%) | 0 (0%) |
|  | *Started (%)* | 58 (18%) | 58 (22%) |
| **Time spent in booster 6**: How to limit conflict |  |  |  |
|  | *Mean (SD)* | 5 (52) | 6 (55) |
|  | *Trimmed mean (SD)* | 0 (0) | 0 (0) |
|  | *Median (IQR)* | 0 (0-0) | 0 (0-0) |
|  | *Range* | 0 - 814 | 0 - 814 |
|  | *Missing (%)* | 19 (6%) | 0 (0%) |
|  | *Started (%)* | 58 (18%) | (22%) |
| **Time spent in booster 7**: Keeping calm when your kids act up |  |  |  |
|  | *Mean (SD)* | 11 (101) | 13 (108) |
|  | *Trimmed mean (SD)* | 0.2 (0.6) | 0.3 (0.7) |
|  | *Median (IQR)* | 0 (0-0.8) | 0 (0-0.9) |
|  | *Range* | 0 - 1435 | 0 - 1435 |
|  | *Missing (%)* | 19 (6%) | 0 (0%) |
|  | *Started (%)* | 89 (28%) | 89 (34%) |
| **Time spent in booster 8**: Using sanctions carefully |  |  |  |
|  | *Mean (SD)* | 3 (23) | 4 (24) |
|  | *Trimmed mean (SD)* | 0 (0) | 0 (0.1) |
|  | *Median (IQR)* | 0 (0-0) | 0 (0-0.1) |
|  | *Range* | 0 - 353 | 0 - 353 |
|  | *Missing (%)* | 19 (6%) | 0 (0%) |
|  | *Started (%)* | 68 (21%) | 68 (26%) |

Table S3: Other SDQ subscale variables* summarised at baseline (T1) by randomised group.

|  |  |  |  |  |
| --- | --- | --- | --- | --- |
|  |  | **By randomised group** | | **Overall (*N*=646)** |
|  |  | FAU group (*n*=326) | *Parent Positive* group (*n*=320) |  |
| **SDQ - hyperactivity, inattention** | | 5.37 (2.90) | 5.23 (2.70) | 5.30 (2.80) |
| **SDQ - peer problems** | | 2.27 (2.23) | 2.20 (2.14) | 2.23 (2.18) |
| **SDQ – prosocial** | | 7.07 (2.30) | 7.08 (2.35) | 7.08 (2.33) |

* if no “Missing” row then data was complete; data are mean (*SD*), median (IQR) or *n* (%)

Table S4: Outcome variables summarised at T2 and T3 by randomised arm, and estimated mean treatment differences with two-sided *CI*s and *p*-values

|  |  |  |  |  |  |  |  |  |  |  |  |  |
| --- | --- | --- | --- | --- | --- | --- | --- | --- | --- | --- | --- | --- |
|  | ***Parent Positive* group (*n*=320)** | | **FAU group (*n*=326)** | | **Overall (*N*=646)** | |  |  |  |  |  |  |
|  |  | |  | | Complete cases (%) |  | **Mean  difference*** | **Standardised effect** | **Two-sided  95%*CI*** | **Two-sided  *p*-value** |  |  |
|  | Complete cases (%) | Mean *(SD)* | Complete cases (%) | Mean *(SD)* |  | Mean *(SD)* |  |  |  |  |  |  |
| **SDQ - conduct at baseline** | 320 (100%) | 2.58 (1.93) | 326 (100%) | 2.71 (2.05) | 646 (100%) | 2.64 (1.99) |  |  |  |  |  |  |
| **SDQ - conduct  at 1 month** | 200 (63%) | 2.48 (2.04) | 266 (82%) | 2.55 (2.01) | 466 (72%) |  | -0.01 | -0.01 | -0.25 to 0.24 | .96 |  |  |
| **SDQ - conduct  at 2 months** | 186 (58%) | 2.19 (1.77) | 256 (79%) | 2.50 (2.1) | 442 (68%) |  | -0.17 | -0.09 | -0.41 to 0.06 | .15 |  |  |
| **SDQ - emotion at baseline** | 320 (100%) | 3.66 (2.63) | 326 (100%) | 3.88 (2.66) | 646 (100%) | 3.77 (2.65) |  |  |  |  |  |  |
| **SDQ - emotion  at 1 month** | 200 (63%) | 3.21 (2.59) | 266 (82%) | 3.71 (2.66) | 466 (72%) |  | -0.35 | -0.13 | -0.65 to -0.05 | .023 |  |  |
| **SDQ - emotion  at 2 months** | 186 (58%) | 3.03 (2.52) | 256 (79%) | 3.45 (2.62) | 442 (68%) |  | -0.35 | -0.13 | -0.67 to -0.04 | .029 |  |  |
| **DASS**  **at baseline** | 320 (100%) | 29.4 (22.8) | 326 (100%) | 33.4 (24.1) | 646 (100%) | 31.4 (23.5) |  |  |  |  |  |  |
| **DASS  at 1 month** | 199 (62%) | 28.4 (22.4) | 264 (81%) | 32.2 (23.4) | 463 (72%) |  | -1.71 | -0.07 | -4.42 to 1.00 | .22 |  |  |
| **DASS  at 2 months** | 185 (58%) | 25.1 (20.1) | 256 (79%) | 28.1 (22.8) | 441 (68%) |  | -0.88 | -0.04 | -3.62 to 1.86 | .53 |  |  |
| **Parental worries at baseline** | 319 (99.7%) | 6.23 (3.64) | 326 (100%) | 6.58 (3.64) | 645 (99.8%) | 6.41 (3.64) |  |  |  |  |  |  |
| **Parental worries at 1 month** | 199 (62%) | 5.48 (3.52) | 266 (82%) | 5.66 (3.26) | 465 (72%) |  | -0.17 | -0.05 | -0.64 to 0.29 | .46 |  |  |
| **Parental worries at 2 months** | 185 (58%) | 5.31 (3.25) | 256 (79%) | 4.91 (3.23) | 441 (68%) |  | 0.51 | 0.14 | 0.04 to 0.98 | .034 |  |  |
| **Family conflict at baseline** | 309 (97%) | 3.10 (1.72) | 314 (96%) | 3.11 (1.75) | 623 (96%) | 3.11 (1.73) |  |  |  |  |  |  |
| **Family conflict  at 1 month** | 192 (60%) | 3.16 (1.74) | 257 (79%) | 3.12 (1.75) | 449 (70%) |  | 0.01 | 0.01 | -0.20 to 0.22 | .93 |  |  |
| **Family conflict  at 2 months** | 179 (56%) | 3.08 (1.62) | 248 (76%) | 3.04 (1.69) | 427 (66%) |  | 0.08 | 0.05 | -0.14 to 0.30 | .47 |  |  |

*estimates of mean differences (*Parent Positive* minus FAU, therefore negative differences imply *Parent Positive* better than FAU, i.e. all measures are higher score = worse) were derived from linear mixed effect models of untransformed measures at both follow-up time-points, and using treatment group, categorical time-point, treatment-time interaction, baseline measure of the outcome, age, gender, household income (less than £30,000 per year vs £30,000 per year or more) and number of adults in the household (one vs two vs three or more). Robust standard errors were modelled in all final analyses.

**Figure S1: Adjusted unstandardised mean outcome differences between randomised arms at the two follow-up time-points T2 and T3, with one-sided and two-sided 95% confidence intervals (note that the DASS outcome is on a different scale)**

**
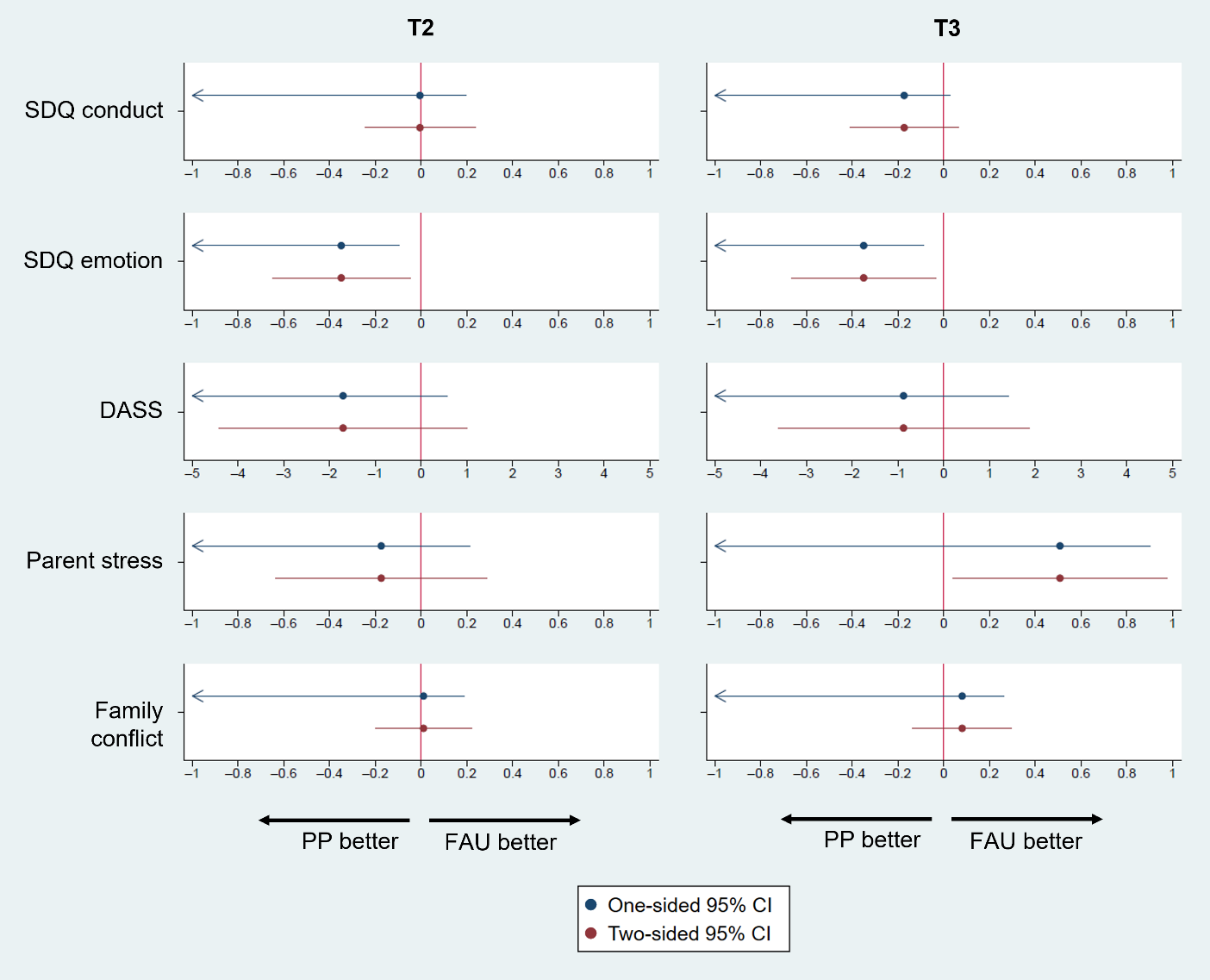
**

**Figure S2: Standardised adjusted mean outcome differences between randomised arms at follow-up time-points T2 and T3, with two-sided 95% confidence intervals**

**
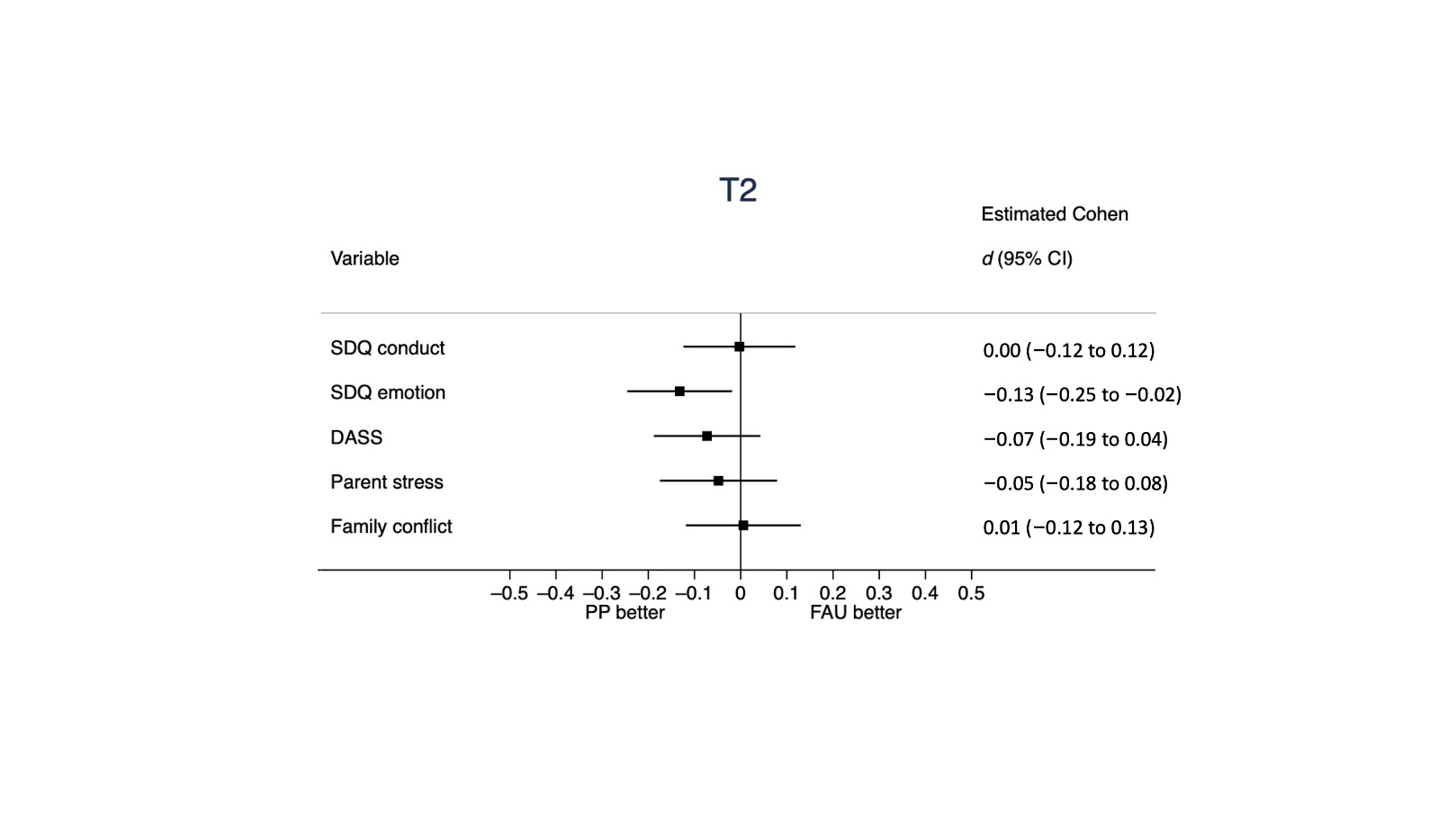

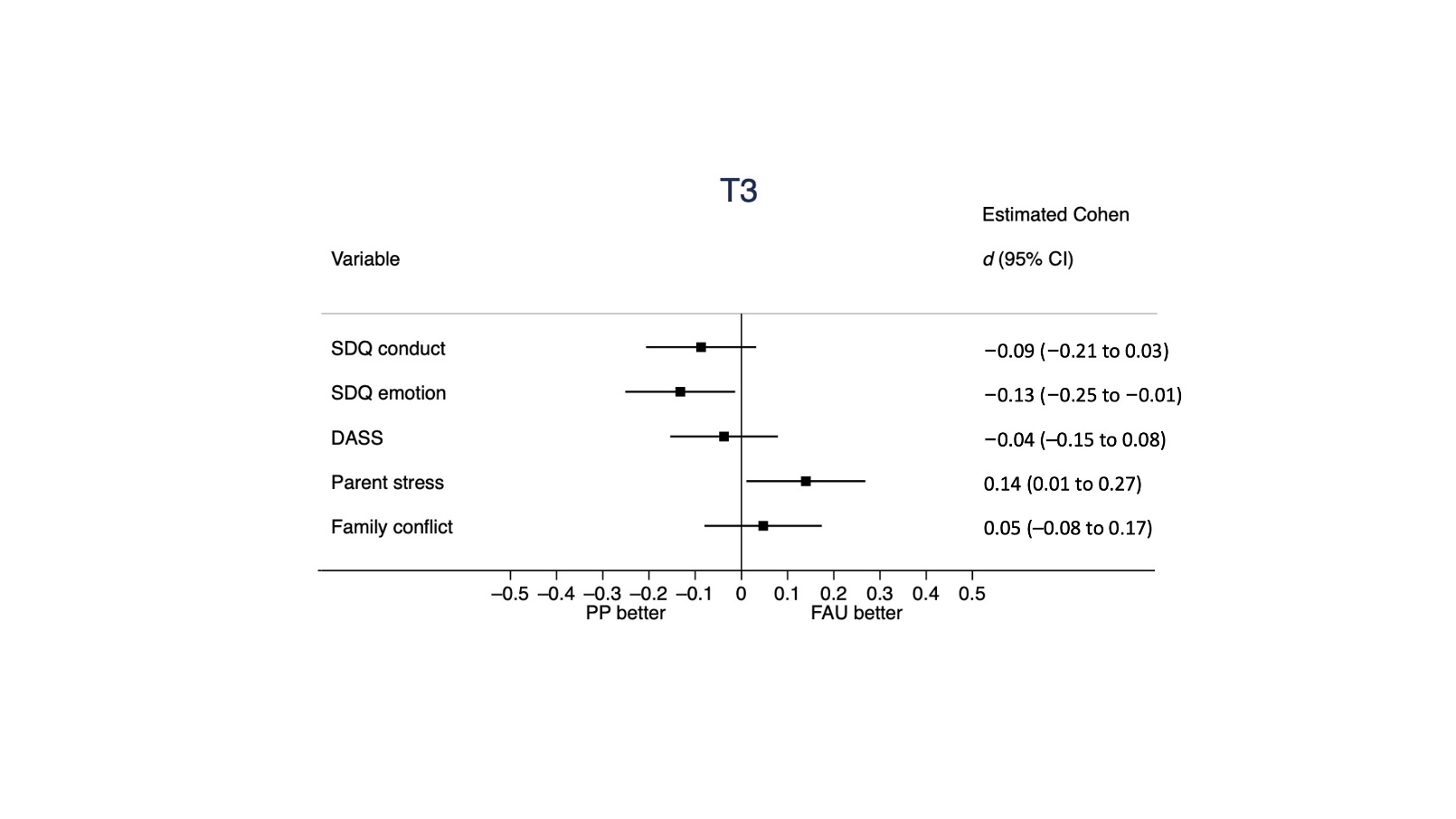
**

**Economic analysis**

Service use, costs and outcomes are summarised in Tables S5-S8, and the results of the primary, sensitivity, and secondary economic analyses are summarised in Table S9. All results are reported excluding influential outliers (those with costs in the 99^th^ percentile), as noted in the main paper, but results including those outliers are also presented to support assessment of the sensitivity of the results to those outliers.

In the primary economic analysis (complete case using QALYs at T2 with outliers removed), the *Parent Positive* group achieved very slightly higher QALYs than the FAU group at a lower cost per participant and thus dominated FAU (Table S9). The probability of *Parent Positive* being cost-effective compared to FAU was 72% and 74% at the NICE willingness to pay thresholds of £20,000 and £30,000 per QALY, respectively. The cost-effectiveness plane in Figure S3 shows the majority of the bootstrapped mean differences in costs and QALYs falling in the southeast quadrant (lower costs and higher effects) and the corresponding cost-effectiveness acceptability curve is shown in Figure S4.

Results were similar in sensitivity analyses using the SDQ conduct subscale in place of QALYs (probability *Parent Positive* cost-effective 64%-66%) and including multiple imputation of missing data (72%-74%), and also for the secondary analysis carried out at T3 (86%-87%) (Table S9). Results were sensitive, however, to the inclusion of influential outliers, with FAU having a higher probability of being cost-effective than *Parent Positive* for all analyses (primary, sensitivity and secondary) including the outliers, as a result of cost differences in favour of FAU (Table S9). The cost-effectiveness plane and cost-effectiveness acceptability curve for the primary economic analysis including outliers are shown in Figures S5 and S6, respectively.

**Table S5: Service use between T1 (baseline) and T2**

|  | ***Parent Positive***  **(*n*=199)** | | **FAU**  **(*n*=264)** | |
| --- | --- | --- | --- | --- |
|  | **Mean (*SD*)** | **% using** | **Mean (*SD*)** | **% using** |
| **Hospital services** |  |  |  |  |
| Inpatient mental health (nights) | 0.02 (0.21) | 0.5 | 0.00 (0.00) | 0.0 |
| Inpatient physical health (nights) | 0.06 (0.54) | 1.5 | 0.02 (0.25) | 0.8 |
| Inpatient accident related (nights) | 0.00 (0.00) | 0.0 | 0.004 (0.06) | 0.4 |
| Outpatient mental health (visits) | 0.02 (0.21) | 0.5 | 0.01 (0.11) | 1.1 |
| Outpatient physical health (visits) | 0.08 (0.32) | 6.5 | 0.11 (0.37) | 9.5 |
| Outpatient accident related (visits) | 0.04 (0.22) | 3.5 | 0.05 (0.30) | 3.8 |
| Accident & Emergency (visits) | 0.07 (0.30) | 5.0 | 0.07 (0.27) | 6.4 |
| Ambulance (attendances) | 0.01 (0.14) | 0.5 | 0.004 (0.06) | 0.4 |
| **Community services** |  |  |  |  |
| GP (visits) | 0.21 (0.66) | 13.1 | 0.12 (0.39) | 9.5 |
| Paediatrician (visits) | 0.08 (0.42) | 5.1 | 0.06 (0.31) | 4.9 |
| Mental health worker (visits) | 0.06 (0.34) | 4.5 | 0.14 (0.88) | 6.1 |
| Social worker (visits) | 0.11 (0.67) | 5.0 | 0.10 (0.65) | 3.4 |
| Practice nurse (visits) | 0.07 (0.36) | 5.0 | 0.02 (0.16) | 1.5 |
| Community nurse (visits) | 0.07 (0.38) | 3.5 | 0.01 (0.11) | 1.1 |
| Therapist (visits) | 0.17 (0.82) | 5.6 | 0.18 (0.75) | 6.8 |
| SALT (visits) | 0.14 (1.44) | 3.5 | 0.13 (0.69) | 5.7 |
| Education psychologist (visits) | 0.07 (0.35) | 5.6 | 0.05 (0.33) | 3.4 |
| Helpline (contacts) | 0.02 (0.22) | 1.0 | 0.01 (0.09) | 0.8 |
| Parenting classes (sessions) | 0.09 (0.52) | 4.5 | 0.04 (0.28) | 2.6 |
| Parent support group (sessions) | 0.07 (0.43) | 3.0 | 0.05 (0.29) | 3.0 |
| **Medications** |  |  |  |  |
| Antidepressants (prescription) |  | 1.0 |  | 0.4 |
| ADHD (prescription) |  | 3.5 |  | 2.3 |
| Sleep disorder (prescription) |  | 2.5 |  | 1.5 |
| Tics/Tourettes (prescription) |  | 4.5 |  | 0.4 |

**Table S6: Service use between T2 and T3**

|  | ***Parent Positive***  **(*n*=186)** | | **FAU**  **(*n*=256)** | |
| --- | --- | --- | --- | --- |
|  | **Mean (*SD*)** | **% using** | **Mean (*SD*)** | **% using** |
| **Hospital Services** |  |  |  |  |
| Inpatient mental health (nights) | 0.01 (0.15) | 1.5 | 0.00 (0.00) | 0.0 |
| Inpatient physical health (nights) | 0.04 (0.39) | 1.0 | 0.01 (0.13) | 1.0 |
| Inpatient accident related (nights) | 0.00 (0.00) | 0.0 | 0.01 (0.02) | 1.0 |
| Outpatient mental health (visits) | 0.05 (0.29) | 4.0 | 0.05 (0.21) | 5.0 |
| Outpatient physical health (visits) | 0.05 (0.39) | 3.0 | 0.02 (0.15) | 2.0 |
| Outpatient accident related (visits) | 0.01 (0.07) | 0.5 | 0.00 (0.00) | 0.0 |
| Accident & Emergency (visits) | 0.04 (0.22) | 3.0 | 0.03 (0.17) | 3.0 |
| Ambulance (attendances) | 0.01 (0.07) | 0.5 | 0.00 (0.00) | 0.0 |
| **Community services** |  |  |  |  |
| GP (visits) | 0.16 (0.58) | 9.0 | 0.09 (0.39) | 7.0 |
| Paediatrician (visits) | 0.08 (0.41) | 5.0 | 0.06 (0.54) | 3.0 |
| Mental health worker (visits) | 0.05 (0.29) | 4.0 | 0.11 (0.82) | 4.0 |
| Social worker (visits) | 0.01 (0.10) | 1.0 | 0.01 (0.06) | 0.5 |
| Practice nurse (visits) | 0.03 (0.27) | 1.5 | 0.05 (0.28) | 4.0 |
| Community nurse (visits) | 0.05 (0.28) | 3.0 | 0.05 (0.52) | 2.5 |
| Therapist (visits) | 0.08 (0.39) | 5.0 | 0.08 (0.44) | 4.5 |
| SALT (visits) | 0.04 (0.31) | 2.0 | 0.09 (0.78) | 4.0 |
| Education psychologist (visits) | 0.11 (1.06) | 2.0 | 0.02 (0.15) | 2.5 |
| Helpline (contacts) | 0.01 (0.07) | 7.5 | 0.01 (0.06) | 8.5 |
| Parenting classes (sessions) | 0.04 (0.33) | 2.0 | 0.02 (0.15) | 1.5 |
| Parent support group (sessions) | 0.04 (0.34) | 1.5 | 0.06 (0.37) | 3.5 |
| **Medications** |  |  |  |  |
| Antidepressants (prescription) |  | 0.5 |  | 2.0 |
| ADHD (prescription) |  | 1.6 |  | 1.6 |
| Sleep disorder (prescription) |  | 0.8 |  | 1.1 |
| Tics/Tourettes (prescription) |  | 3.2 |  | 2.0 |

**Table S7: Disaggregated mean costs (£)**

|  | **T1 to T2** |  | **T2 to T3** |  | **T1 to T3*** |  |
| --- | --- | --- | --- | --- | --- | --- |
|  | ***Parent Positive*** | **FAU** | ***Parent Positive*** | **FAU** | ***Parent Positive*** | **FAU** |
| **Category of cost** | **Mean (*SD*)** | **Mean (*SD*)** | **Mean (*SD*)** | **Mean (*SD*)** | **Mean (*SD*)** | **Mean (*SD*)** |
| **Outliers excluded** | N=196 | N=263 | N=183 | N=255 | N=183 | N=255 |
| Intervention | 21 (0) | 0 (0) | 0 (0) | 0 (0) | 21 (0) | 0 (0) |
| Hospital mental health | 0 (0) | 3 (25) | 9 (43) | 10 (49) | 9 (43) | 14 (57) |
| Hospital other | 36 (134) | 57 (208) | 6 (40) | 15 (86) | 36 (131) | 74 (244) |
| Community services | 85 (261) | 115 (366) | 29 (89) | 47 (174) | 105 (338) | 145 (391) |
| Medications | 2 (12) | 2 (12) | 1 (10) | 1 (10) | 2 (17) | 3 (20) |
| Total Costs | 144 (326) | 176 (445) | 44 (104) | 73 (213) | 168 (379) | 237 (537) |
| **Outliers included** | N=199 | N=264 | N=186 | N=256 | N=186 | N=256 |
| Intervention | 21 (0) | 0 (0) | 0 (0) | 0 (0) | 21 (0) | 0 (0) |
| Hospital mental health | 18 (251) | 3 (25) | 23 (195) | 11 (51) | 27 (206) | 14 (57 |
| Hospital other | 74 (437) | 59 (210) | 51 (489) | 18 (100) | 126 (928) | 76 (246) |
| Community services | 125 (394) | 132 (405) | 60 (268) | 53 (200) | 159 (453) | 162 (471) |
| Medications | 3 (15) | 2 (12) | 1 (10) | 1 (10) | 3 (19) | 3 (20) |
| Total Costs | 241 (927) | 196 (491) | 136 (775) | 83 (269) | 337 (1658) | 255 (613) |

* Participants who did not complete the T2 CA-SUS were asked about the previous two months in the T3 CA-SUS, hence T1 to T3 mean costs are not the sum of T1 to T2 one month mean costs and one month mean costs T2 to T3.

**Table S8: CHU9D scores and QALYs**

|  |  | ***Parent Positive*** |  | **FAU** |  |
| --- | --- | --- | --- | --- | --- |
|  | ***N*** | **Mean (*SD*)** | ***n*** | **Mean (*SD*)** | **Unadjusted difference** |
| **Outliers excluded** | |  |  |  |  |
| T1 CHU9D | 317 | 0.800533 (0.072950) | 325 | 0.794021 (0.076049) | 0.006512 |
| T2 CHU9D | 197 | 0.810558 (0.074116) | 265 | 0.800811 (0.075847) | 0.010175 |
| T3 CHU9D | 183 | 0.816683 (0.068956) | 255 | 0.807290 (0.076349) | 0.009393 |
| T2 QALYS | 197 | 0.000346 (0.001881) | 265 | 0.000212 (0.001622) | 0.000134 |
| T3 QALYS | 183 | 0.000569 (0.002697) | 255 | 0.000315 (0.001888) | 0.000254 |
| **Outliers included** | |  |  |  |  |
| T1 CHU9D | 320 | 0.799940 (0.073380) | 326 | 0.793785 (0.076053) | 0.006155 |
| T2 CHU9D | 200 | 0.809650 (0.074134) | 266 | 0.800383 (0.076025) | 0.009267 |
| T3 CHU9D | 186 | 0.816435 (0.069035) | 256 | 0.806855 (0.076516) | 0.009580 |
| T2 QALYS | 200 | 0.000349 (0.001896) | 266 | 0.000207 (0.001622) | 0.000142 |
| T3 QALYS | 186 | 0.000595 (0.002775) | 256 | 0.000311 (0.001886) | 0.000284 |

**Table S9: Summary of primary, sensitivity and secondary economic analyses**

|  | **Mean difference in costs^1^** | **Mean difference in outcomes^1^** | **ICER^1^** | **Probability cost-effective (%)^1^** |
| --- | --- | --- | --- | --- |
| **Outliers excluded** |  |  |  |  |
| Primary economic analysis: complete case using QALYs at T2 | -£19 | 0.000132 | Dominant | 72.1% to 73.6%^3^ |
| Sensitivity analysis: complete case using SDQ conduct subscale at T2 | -£19 | -0.013570 | Dominant | 64.4% to 66.2%^4^ |
| Sensitivity analysis: multiple imputation^2^ using QALYs at T2 | -£18 | 0.000161 | Dominant | 72.2% to 73.6%^3^ |
| Secondary economic analysis: complete case using QALYs at T3 | -£45 | 0.000188 | Dominant | 86.4% to 86.8%^3^ |
| **Outliers included** |  |  |  |  |
| Primary economic analysis: complete case using QALYs at T2 | £78 | 0.000151 | £516,241 | 19.3% to 19.8%^3^ |
| Sensitivity analysis: complete case using SDQ conduct subscale at T2 | £78 | -0.008709 | £11,143 | 18.2% to 18.4%^4^ |
| Sensitivity analysis: multiple imputation^2^ using QALYs at T2 | £53 | 0.0001455 | £365,636 | 23.5% to 24.5%^3^ |
| Secondary economic analysis: complete case using QALYs at T3 | £165 | 0.0002755 | £598,911 | 10.6% to 11.2%^3^ |

^1^Differences in costs and outcomes and analysis of cost-effectiveness were adjusted for treatment group, baseline measure of the outcome of interest, age, gender, household income (less than £30,000 per year vs. £30,000 per year or more) and number of adults in the household (one vs two vs three or more). ^2^Missing cost and outcome data were imputed using multiple imputation using chained equations (MICE) following White et al., Multiple imputation using chained equations: Issues and guidance for practice. *Statistics in Medicine*, 2011 Feb 20;30(4):377–99. ^3^Based on a willingness-to-pay for a QALY between £20,000 and £30,000; ^4^Based on a willingness-to-pay for a one-point improvement in SDQ conduct score between £0 and £1,000

**Figure S3:** **Scatterplot showing the bootstrapped mean differences in costs and QALYs for *Parent Positive* compared to FAU at T2 excluding outliers**

**Figure S4: Cost-effectiveness acceptability curve showing the probability that *Parent Positive* is cost-effective compared to FAU in terms of QALYs at T2 excluding outliers**

0

20

40

60

80

100

**Probability *Parent Positive* is cost-effective**

0

£10000

£20000

£30000

**Willingness to pay for improvement in QALYs**

**Figure S5: Scatterplot showing the bootstrapped mean differences in costs and QALYs for Parent Positive compared to FAU at T2 including outliers**


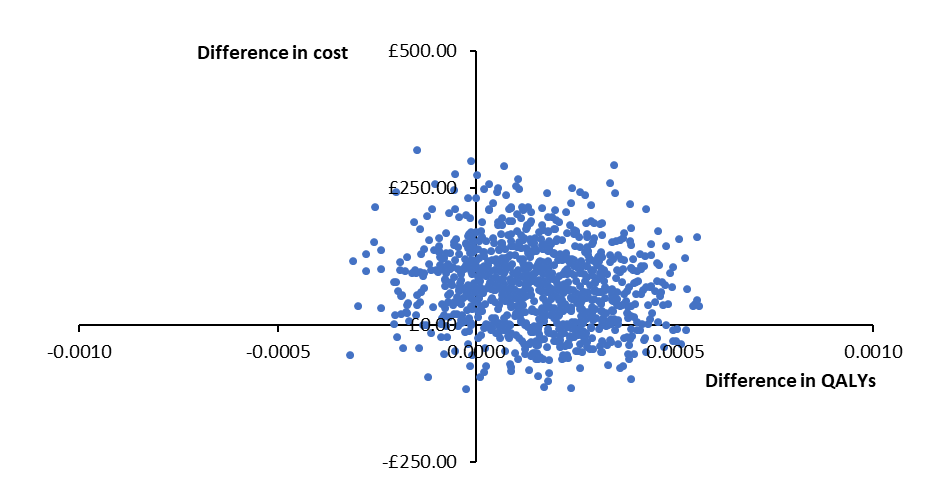


**Figure S6: Cost-effectiveness acceptability curve showing the probability that *Parent Positive* is cost-effective compared to FAU in terms of QALYs at T2 including outliers**

0

20

40

60

80

100

**Probability *Parent Positive* is cost-effective**

0

£10000

£20000

£30000

**Willingness to pay for improvement in QALYs**

**CONSORT-SPI 2018 Checklist**

| **SECTION** | **ITEM #** | **CONSORT-SPI 2010** | **CONSORT-SPI**  **2018** | **REPORTED ON PAGE #** |
| --- | --- | --- | --- | --- |
| **TITLE AND ABSTRACT** | | | | |
|  | 1a | Identification as a randomised trial in the title^§^ |  | 1 |
|  | 1b | Structured summary of trial design, methods, results, and conclusions (for specific guidance see CONSORT for Abstracts)^§^ | Refer to CONSORT extension for social and psychological intervention trial abstracts | 1-2 |
| **INTRODUCTION** | | | | |
| Background and  Objectives | 2a | Scientific background and explanation of rationale ^§^ |  | 3-4 |
|  | 2b | Specific objectives or hypotheses ^§^ | If pre-specified, how the intervention was hypothesied to work | 3-4 |
| **METHODS** | | | | |
| Trial Design | 3a | Describe of trial design (such as parallel, factorial), including allocation ratio ^§^ | If the unit of random assignment is not the individual, please refer to CONSORT for Cluster Randomized Trials | 4 |
|  | 3b | Important changes to methods after trial commencement (such as eligibility criteria), with reasons |  | N/A, 4 |
| Participants | 4a | Eligibility criteria for participants^§^ | When applicable, eligibility criteria for settings and those delivering the interventions | 4-5 |
|  | 4b | Settings and locations where the data were collected |  | 6-7 |
| Interventions | 5 | The interventions for each group with sufficient details to allow replication, including how and when they are actually administered ^§^ |  | 5-6 |
|  | 5a |  | Extent to which interventions were actually delivered by providers and taken up by participants as planned | 13, Online Supplement |
|  | 5b |  | Where other informational materials about delivering the intervention can be accessed | 5-6, Protocol, Online Supplement |
|  | 5c |  | When applicable, how intervention providers were assigned to each group | N/A |
| Outcomes | 6a | Completely defined pre-specified outcomes, including how and when they were assessed^§^ |  | 7-9 |
|  | 6b | Any changes to trial outcomes after the trial commenced, with reasons |  | N/A, 4 |
| Sample Size | 7a | How sample size was determined^§^ |  | 9 |
|  | 7b | When applicable, explanation of any interim analyses and stopping guidelines |  | 12 |
| **RANDOMISATION** | | | | |
| Sequence  generation | 8a | Method used to generate the random allocation sequence |  | 5 |
|  | 8b | Type of randomisation; detail of any restriction (such as blocking and block size)^§^ |  | 5 |
| Allocation concealment mechanism | 9 | Mechanism used to implement the random allocation sequence, describing any steps taken to conceal the sequence until interventions were assigned^§^ |  | 5 |
| Implementation | 10 | Who generated the random allocation sequence, who enrolled participants, and who assigned participants to interventions^§^ |  | 5 |
| Awareness of assignment | 11a | Who was aware of intervention assignment after allocation (for example, participants, providers, those assessing outcomes), and how any masking was done |  | 5 |
|  | 11b | If relevant, description of the similarity of interventions |  | N/A |
| Analytical  methods | 12a | Statistical methods used to compare group outcomes^§^ | How missing data were handled, with details of any imputation method | 9-11 |
|  | 12b | Methods for additional analyses, such as subgroup analyses, adjusted analyses, and process evaluations |  | 9-11 |
| **RESULTS** | | | | |
| Participant flow (a diagram is strongly recommended) | 13a | For each group, the numbers randomly assigned, receiving the intended intervention, and analysed for the outcomes^§^ | Where possible, the number approached, screened, and eligible prior to random assignment, with reasons for non-enrolment | 12, Figure 1 |
|  | 13b | For each group, losses and exclusions after randomisation, together with reasons^§^ |  | Figure 1 |
| Recruitment | 14a | Dates defining the periods of recruitment and follow-up |  | 12, Figure 1 |
|  | 14b | Why the trial ended or was stopped |  | 12 |
| Baseline data | 15 | A table showing baseline characteristics for each group^§^ | Include socioeconomic variables where applicable | Table 1a &1b |
| Numbers analysed | 16 | For each group, number included in each analysis and whether the analysis was by original assigned groups^§^ |  | 13 |
| Outcomes and estimation | 17a | For each outcome, results for each group, and the estimated effect size and its precision (such as 95% confidence interval)^§^ | Indicate availability of trial data | 13-15, Table 3 |
|  | 17b | For binary outcomes, the presentation of both absolute and relative effect sizes is recommended |  | N/A |
| Ancillary analyses | 18 | Results of any other analyses performed, including subgroup analyses, adjusted analyses, and process evaluations, distinguishing pre-specified from exploratory |  | Online Supplement |
| Harms | 19 | All important harms or unintended effects in each group (for specific guidance see CONSORT for Harms) |  | 15 |
| **DISCUSSION** | | | | |
| Limitations | 20 | Summarize the main results (including an overview of concepts, themes, and types of evidence available), link to the review questions and objectives, and consider the relevance to key groups. | Trial limitations, addressing sources of potential bias, imprecision, and, if relevant, multiplicity of analyses | 19-20 |
| Generalisability | 21 | Discuss the limitations of the scoping review process. | Generalisability (external validity, applicability) of the trial findings^§^ | 19-20 |
| Interpretation | 22 | Provide a general interpretation of the results with respect to the review questions and objectives, as well as potential implications and/or next steps. | Interpretation consistent with results, balancing benefits and harms, and considering other relevant evidence | 16-20 |
| **IMPORTANT INFORMATION** | | | | |
| Registration | 23 | Registration number and name of trial registry |  | Abstract |
| Protocol | 24 | Where the full trial protocol can be accessed, if available |  | 4 |
| Declaration of Interests | 25 | Sources of funding and other support; role of funders | Declaration of any other potential interests | Title page |
| Stakeholder investments | 26a |  | Any involvement of the intervention developer in the design, conduct, analysis, or reporting of the trial | Title page |
|  | 26b |  | Other stakeholder involvement in trial design, conduct, or analyses | Title page |
|  | 26c |  | Incentives offered as part of the trial | 6-7 |

This table lists items from the CONSORT 2010 checklist (with some modifications for social and psychological intervention trials) and additional items in the CONSORT-SPI 2018 extension [41]. Empty rows in the ‘CONSORT-SPI 2018’ column indicate that there is no extension to the CONSORT 2010 item

*We strongly recommended that the CONSORT-SPI 2018 Explanation and Elaboration (E&E) document [42] be reviewed when using the CONSORT-SPI 2018 checklist for important clarifications on each item

§An extension item for cluster trials exists for this CONSORT 2010 item
