# Supplementary material for: The Effectiveness and Cost-Effectiveness of a Universal Digital Parenting Intervention Designed and Implemented During the COVID-19 Pandemic: Evidence From a Rapid-Implementation Randomized Controlled Trial Within a Cohort
Source: J Med Internet Res. 2023 Jul 27;25:e44079. doi: 10.2196/44079 (PMC10415938; doi:10.2196/44079)
Supplement: Multimedia Appendix 2 [file jmir_v25i1e44079_app2.pdf]

CONSORT-EHEALTH (V 1.6.1) - Submission/Publication Form

The CONSORT-EHEALTH checklist is intended for authors of randomized trials evaluating web-based and Internet-based applications/interventions, including mobile interventions, electronic games (incl multiplayer games), social media, certain telehealth applications, and other interactive and/or networked electronic applications. Some of the items (e.g. all subitems under item 5 - description of the intervention) may also be applicable for other study designs.

The goal of the CONSORT EHEALTH checklist and guideline is to be
a) a guide for reporting for authors of RCTs,
b) to form a basis for appraisal of an ehealth trial (in terms of validity)

CONSORT-EHEALTH items/subitems are MANDATORY reporting items for studies published in the Journal of Medical Internet Research and other journals / scientific societies endorsing the checklist.

Items numbered 1., 2., 3., 4a., 4b etc are original CONSORT or CONSORT-NPT (non-pharmacologic treatment) items.
Items with Roman numerals (i., ii, iii, iv etc.) are CONSORT-EHEALTH extensions/clarifications.

As the CONSORT-EHEALTH checklist is still considered in a formative stage, we would ask that you also RATE ON A SCALE OF 1-5 how important/useful you feel each item is FOR THE PURPOSE OF THE CHECKLIST and reporting guideline (optional).

Mandatory reporting items are marked with a red *.
In the textboxes, either copy & paste the relevant sections from your manuscript into this form - please include any quotes from your manuscript in QUOTATION MARKS,
or answer directly by providing additional information not in the manuscript, or elaborating on why the item was not relevant for this study.

YOUR ANSWERS WILL BE PUBLISHED AS A SUPPLEMENTARY FILE TO YOUR PUBLICATION IN JMIR AND ARE CONSIDERED PART OF YOUR PUBLICATION (IF ACCEPTED).
Please fill in these questions diligently. Information will not be copyedited, so please use proper spelling and grammar, use correct capitalization, and avoid abbreviations.

DO NOT FORGET TO SAVE AS PDF _AND_ CLICK THE SUBMIT BUTTON SO YOUR ANSWERS ARE IN OUR DATABASE !!!

Citation Suggestion (if you append the pdf as Appendix we suggest to cite this paper in the caption):
Eysenbach G, CONSORT-EHEALTH Group
CONSORT-EHEALTH: Improving and Standardizing Evaluation Reports of Web-based and Mobile Health Interventions
J Med Internet Res 2011;13(4):e126

URL: http://www.jmir.org/2011/4/e126/
doi: 10.2196/jmir.1923
PMID: 22209829

melanie.l.palmer@gmail.com Switch account

Not shared

Draft saved

* Indicates required question

Your name *

First Last

Melanie Palmer

Primary Affiliation (short), City, Country *

University of Toronto, Toronto, Canada

King's College London, London, United Kingdor

Your e-mail address *

abc@gmail.com

melanie.palmer@kcl.ac.uk

Title of your manuscript *

Provide the (draft) title of your manuscript.

The Effectiveness and Cost-Effectiveness of a Universal Digital Parenting Intervention Designed and Implemented During the COVID-19 Pandemic: Evidence From a Rapid-Implementation Randomized Controlled Trial Within a Cohort

Name of your App/Software/Intervention *

If there is a short and a long/alternate name, write the short name first and add the long name in brackets.

Parent Positive app

Evaluated Version (if any)

e.g. "V1", "Release 2017-03-01", "Version 2.0.27913"

1.1.14, last updated 10/09/2021

Language(s) *

What language is the intervention/app in? If multiple languages are available, separate by comma (e.g. "English, French")

English

URL of your Intervention Website or App

e.g. a direct link to the mobile app on app in appstore (itunes, Google Play), or URL of the website. If the intervention is a DVD or hardware, you can also link to an Amazon page.

https://apps.apple.com/gb/app/parent-positive/id1554443712; https://play.google.com/stor

URL of an image/screenshot (optional)

Your answer

Accessibility *

Can an enduser access the intervention presently?

- ○ access is free and open
- ◉ access only for special usergroups, not open
- ○ access is open to everyone, but requires payment/subscription/in-app purchases
- ○ app/intervention no longer accessible
- ○ Other:

Primary Medical Indication/Disease/Condition *

e.g. "Stress", "Diabetes", or define the target group in brackets after the condition, e.g. "Autism (Parents of children with)", "Alzheimers (Informal Caregivers of)"

Children’s conduct and emotional problems

Primary Outcomes measured in trial *

comma-separated list of primary outcomes reported in the trial

Child conduct problems

Secondary/other outcomes

Are there any other outcomes the intervention is expected to affect?

Child emotional problems; Parental psychological distress; Child-related parental worries; Family conflict

Recommended "Dose" *

What do the instructions for users say on how often the app should be used?

- ○ Approximately Daily
- ○ Approximately Weekly
- ○ Approximately Monthly
- ○ Approximately Yearly
- ● "as needed"
- ○ Other:

Approx. Percentage of Users (starters) still using the app as recommended after 3 months *

- ● unknown / not evaluated
- ○ 0-10%
- ○ 11-20%
- ○ 21-30%
- ○ 31-40%
- ○ 41-50%
- ○ 51-60%
- ○ 61-70%
- ○ 71%-80%
- ○ 81-90%
- ○ 91-100%
- ○ Other:

Overall, was the app/intervention effective? *

- ○ yes: all primary outcomes were significantly better in intervention group vs control
- ○ partly: SOME primary outcomes were significantly better in intervention group vs control
- ○ no statistically significant difference between control and intervention
- ○ potentially harmful: control was significantly better than intervention in one or more outcomes
- ○ inconclusive: more research is needed
- ◉ Other: partly: some secondary outcomes were significantly better in interven

Article Preparation Status/Stage *

At which stage in your article preparation are you currently (at the time you fill in this form)

- ○ not submitted yet - in early draft status
- ○ not submitted yet - in late draft status, just before submission
- ○ submitted to a journal but not reviewed yet
- ○ submitted to a journal and after receiving initial reviewer comments
- ◉ submitted to a journal and accepted, but not published yet
- ○ published
- ○ Other:

Journal *

If you already know where you will submit this paper (or if it is already submitted), please provide the journal name (if it is not JMIR, provide the journal name under "other")

- ( ) not submitted yet / unclear where I will submit this
- (•) Journal of Medical Internet Research (JMIR)
- ( ) JMIR mHealth and UHealth
- ( ) JMIR Serious Games
- ( ) JMIR Mental Health
- ( ) JMIR Public Health
- ( ) JMIR Formative Research
- ( ) Other JMIR sister journal
- ( ) Other:

Is this a full powered effectiveness trial or a pilot/feasibility trial? *

- ( ) Pilot/feasibility
- (•) Fully powered

Manuscript tracking number *

If this is a JMIR submission, please provide the manuscript tracking number under "other" (The ms tracking number can be found in the submission acknowledgement email, or when you login as author in JMIR. If the paper is already published in JMIR, then the ms tracking number is the four-digit number at the end of the DOI, to be found at the bottom of each published article in JMIR)

- ( ) no ms number (yet) / not (yet) submitted to / published in JMIR
- (•) Other: 44079

TITLE AND ABSTRACT

1a) TITLE: Identification as a randomized trial in the title

1a) Does your paper address CONSORT item 1a? *

I.e does the title contain the phrase "Randomized Controlled Trial"? (if not, explain the reason under "other")

- (●) yes
- ( ) Other:

1a-i) Identify the mode of delivery in the title

Identify the mode of delivery. Preferably use “web-based” and/or “mobile” and/or “electronic game” in the title. Avoid ambiguous terms like “online”, “virtual”, “interactive”. Use “Internet-based” only if Intervention includes non-web-based Internet components (e.g. email), use “computer-based” or “electronic” only if offline products are used. Use “virtual” only in the context of “virtual reality” (3-D worlds). Use “online” only in the context of “online support groups”. Complement or substitute product names with broader terms for the class of products (such as “mobile” or “smart phone” instead of “iphone”), especially if the application runs on different platforms.

subitem not at all important

- 1 ( )
- 2 ( )
- 3 ( )
- 4 ( )
- 5 ( )

essential

Does your paper address subitem 1a-i? *

Copy and paste relevant sections from manuscript title (include quotes in quotation marks "like this" to indicate direct quotes from your manuscript), or elaborate on this item by providing additional information not in the ms, or briefly explain why the item is not applicable/relevant for your study

"The Effectiveness and Cost-Effectiveness of a Universal Digital Parenting Intervention Designed and Implemented During the COVID-19 Pandemic: Evidence From a Rapid-Implementation Randomized Controlled Trial Within a Cohort"

1a-ii) Non-web-based components or important co-interventions in title

Mention non-web-based components or important co-interventions in title, if any (e.g., “with telephone support”).

subitem not at all important

1 ○

2 ○

3 ○

4 ○

5 ○

essential

Does your paper address subitem 1a-ii?

Copy and paste relevant sections from manuscript title (include quotes in quotation marks "like this" to indicate direct quotes from your manuscript), or elaborate on this item by providing additional information not in the ms, or briefly explain why the item is not applicable/relevant for your study

Your answer

1a-iii) Primary condition or target group in the title

Mention primary condition or target group in the title, if any (e.g., “for children with Type I Diabetes”) Example: A Web-based and Mobile Intervention with Telephone Support for Children with Type I Diabetes: Randomized Controlled Trial

subitem not at all important

1 ○

2 ○

3 ○

4 ○

5 ○

essential

Does your paper address subitem 1a-iii? *

Copy and paste relevant sections from manuscript title (include quotes in quotation marks "like this" to indicate direct quotes from your manuscript), or elaborate on this item by providing additional information not in the ms, or briefly explain why the item is not applicable/relevant for your study

"The Effectiveness and Cost-Effectiveness of a Universal Digital Parenting Intervention Designed and Implemented During the COVID-19 Pandemic: Evidence From a Rapid-Implementation Randomized Controlled Trial Within a Cohort"

1b) ABSTRACT: Structured summary of trial design, methods, results, and conclusions

NPT extension: Description of experimental treatment, comparator, care providers, centers, and blinding status.

1b-i) Key features/functionalities/components of the intervention and comparator in the METHODS section of the ABSTRACT

Mention key features/functionalities/components of the intervention and comparator in the abstract. If possible, also mention theories and principles used for designing the site. Keep in mind the needs of systematic reviewers and indexers by including important synonyms. (Note: Only report in the abstract what the main paper is reporting. If this information is missing from the main body of text, consider adding it)

subitem not at all important

1 ○

2 ○

3 ○

4 ○

5 ○

essential

Does your paper address subitem 1b-i? *

Copy and paste relevant sections from the manuscript abstract (include quotes in quotation marks "like this" to indicate direct quotes from your manuscript), or elaborate on this item by providing additional information not in the ms, or briefly explain why the item is not applicable/relevant for your study

"Objective: We tested whether a smartphone parenting support app, Parent Positive, developed specifically for this purpose reversed these effects in a cost-effective way. Parent Positive includes 3 zones. Parenting Boosters (zone 1) provided content adapted from standard face-to-face parent training programs to tackle 8 specific challenges identified by parents and parenting experts as particularly relevant for parents during the pandemic. The Parenting Exchange (zone 2) was a parent-to-parent and parent-to-expert communication forum. Parenting Resources (zone 3) provided access to existing high-quality web-based resources on a range of additional topics of value to parents (eg, neurodevelopmental problems, diet, and sleep).
Methods: Supporting Parents And Kids Through Lockdown Experiences (SPARKLE), a randomized controlled trial (NCT04786080), was embedded in the UK-wide COVID-19: Supporting Parents, Adolescents and Children during Epidemics (Co-SPACE) longitudinal study on families' mental health during the pandemic. Parents of children aged 4 to 10 years were randomized 1:1 to Parent Positive or Follow-up As Usual (FAU) between May 19, 2021, and July 26, 2021. Parent Positive provided advice on common parenting challenges, evidence-based web-based resources and facilitated parent-to-parent and expert-to-parent support. Child conduct and emotional problems and family well-being were measured before randomization (T1) and 1 (T2) and 2 (T3) months after randomization. Service use, costs, and adverse events were measured, along with app use and satisfaction. The primary outcome was T2 parent-reported child conduct problems, which were analyzed using linear mixed regression models.
Results: A total of 320 participants were randomized to Parent Positive, and 326 participants were randomized to FAU. The primary outcome analysis included 79.3% (512/646) of the participants (dropout: 84/320, 26% on Parent Positive and 50/326, 15% on FAU). There were no statistically significant intervention effects on conduct problems at either T2 (standardized effect=−0.01) or T3 (secondary outcome; standardized effect=−0.09) and no moderation by baseline conduct problems. Significant intervention-related reductions in emotional problems were observed at T2 and T3 (secondary outcomes; standardized effect=−0.13 in both cases). Parent Positive, relative to FAU, was associated with more parental worries at T3 (standardized effect=0.14). Few intervention-attributable adverse events were reported. Parent Positive was cost-effective once 4 outliers with extremely high health care costs were excluded.
Conclusions: Parent Positive reduced child emotional problems and was cost-effective compared with FAU once outliers were removed. Although small when considered against targeted therapeutic interventions, the size of these effects was in line with trials of nontargeted universal mental health interventions. This highlights the public health potential of Parent Positive if implemented at the community level. Nevertheless, caution is required before making such an interpretation, and the findings need to be

1b-ii) Level of human involvement in the METHODS section of the ABSTRACT

Clarify the level of human involvement in the abstract, e.g., use phrases like “fully automated” vs. “therapist/nurse/care provider/physician-assisted” (mention number and expertise of providers involved, if any). (Note: Only report in the abstract what the main paper is reporting. If this information is missing from the main body of text, consider adding it)

subitem not at all important

1 ◯

2 ◯

3 ◯

4 ◯

5 ◯

essential

Does your paper address subitem 1b-ii?

Copy and paste relevant sections from the manuscript abstract (include quotes in quotation marks "like this" to indicate direct quotes from your manuscript), or elaborate on this item by providing additional information not in the ms, or briefly explain why the item is not applicable/relevant for your study

Your answer

1b-iii) Open vs. closed, web-based (self-assessment) vs. face-to-face assessments in the METHODS section of the ABSTRACT

Mention how participants were recruited (online vs. offline), e.g., from an open access website or from a clinic or a closed online user group (closed usergroup trial), and clarify if this was a purely web-based trial, or there were face-to-face components (as part of the intervention or for assessment). Clearly say if outcomes were self-assessed through questionnaires (as common in web-based trials). Note: In traditional offline trials, an open trial (open-label trial) is a type of clinical trial in which both the researchers and participants know which treatment is being administered. To avoid confusion, use “blinded” or “unblinded” to indicated the level of blinding instead of “open”, as “open” in web-based trials usually refers to “open access” (i.e. participants can self-enrol). (Note: Only report in the abstract what the main paper is reporting. If this information is missing from the main body of text, consider adding it)

subitem not at all important

1 ○

2 ○

3 ○

4 ○

5 ○

essential

Does your paper address subitem 1b-iii?

Copy and paste relevant sections from the manuscript abstract (include quotes in quotation marks "like this" to indicate direct quotes from your manuscript), or elaborate on this item by providing additional information not in the ms, or briefly explain why the item is not applicable/relevant for your study

Your answer

1b-iv) RESULTS section in abstract must contain use data

Report number of participants enrolled/assessed in each group, the use/uptake of the intervention (e.g., attrition/adherence metrics, use over time, number of logins etc.), in addition to primary/secondary outcomes. (Note: Only report in the abstract what the main paper is reporting. If this information is missing from the main body of text, consider adding it)

subitem not at all important

1 ○

2 ○

3 ○

4 ○

5 ○

essential

Does your paper address subitem 1b-iv?

Copy and paste relevant sections from the manuscript abstract (include quotes in quotation marks "like this" to indicate direct quotes from your manuscript), or elaborate on this item by providing additional information not in the ms, or briefly explain why the item is not applicable/relevant for your study

Your answer

1b-v) CONCLUSIONS/DISCUSSION in abstract for negative trials

Conclusions/Discussions in abstract for negative trials: Discuss the primary outcome - if the trial is negative (primary outcome not changed), and the intervention was not used, discuss whether negative results are attributable to lack of uptake and discuss reasons. (Note: Only report in the abstract what the main paper is reporting. If this information is missing from the main body of text, consider adding it)

subitem not at all important

1 ○

2 ○

3 ○

4 ○

5 ○

essential

Does your paper address subitem 1b-v?

Copy and paste relevant sections from the manuscript abstract (include quotes in quotation marks "like this" to indicate direct quotes from your manuscript), or elaborate on this item by providing additional information not in the ms, or briefly explain why the item is not applicable/relevant for your study

Your answer

INTRODUCTION

2a) In INTRODUCTION: Scientific background and explanation of rationale

2a-i) Problem and the type of system/solution

Describe the problem and the type of system/solution that is object of the study: intended as stand-alone intervention vs. incorporated in broader health care program? Intended for a particular patient population? Goals of the intervention, e.g., being more cost-effective to other interventions, replace or complement other solutions? (Note: Details about the intervention are provided in “Methods” under 5)

subitem not at all important

1 ○

2 ○

3 ○

4 ○

5 ○

essential

Does your paper address subitem 2a-i? *

Copy and paste relevant sections from the manuscript (include quotes in quotation marks "like this" to indicate direct quotes from your manuscript), or elaborate on this item by providing additional information not in the ms, or briefly explain why the item is not applicable/relevant for your study

"UK COVID-19 mitigation strategies (eg, extended joint confinement, social isolation, and homeschooling) presented families with unprecedented challenges [1]. Children’s behavioral and emotional problems increased during the pandemic [2], with individuals with preexisting vulnerabilities at particular risk [3]. COVID-19: Supporting Parents, Adolescents and Children during Epidemics (Co-SPACE), a UK-wide general population study, confirmed this pattern during both the first and second UK lockdowns [4,5]. Findings from Co-SPACE and other studies show that increases in parental stress and child-related help seeking were also reported [6,7].
Providing parents with advice and support can reduce children’s conduct and emotional problems [9] and parent-related stress [10]. However, access to face-to-face parenting support interventions was severely limited during the lockdowns. To circumvent this obstacle and reverse pandemic-related increases in children’s conduct and emotional problems, we developed Parent Positive—a smartphone app providing evidence-based information and support to the general population."

2a-ii) Scientific background, rationale: What is known about the (type of) system

Scientific background, rationale: What is known about the (type of) system that is the object of the study (be sure to discuss the use of similar systems for other conditions/diagnoses, if appropiate), motivation for the study, i.e. what are the reasons for and what is the context for this specific study, from which stakeholder viewpoint is the study performed, potential impact of findings [2]. Briefly justify the choice of the comparator.

subitem not at all important

1 ○

2 ○

3 ○

4 ○

5 ○

essential

Does your paper address subitem 2a-ii? *

Copy and paste relevant sections from the manuscript (include quotes in quotation marks "like this" to indicate direct quotes from your manuscript), or elaborate on this item by providing additional information not in the ms, or briefly explain why the item is not applicable/relevant for your study

"UK COVID-19 mitigation strategies (eg, extended joint confinement, social isolation, and homeschooling) presented families with unprecedented challenges [1]. Children's behavioral and emotional problems increased during the pandemic [2], with individuals with preexisting vulnerabilities at particular risk [3]. COVID-19: Supporting Parents, Adolescents and Children during Epidemics (Co-SPACE), a UK-wide general population study, confirmed this pattern during both the first and second UK lockdowns [4,5]. Findings from Co-SPACE and other studies show that increases in parental stress and child-related help seeking were also reported [6,7].
Providing parents with advice and support can reduce children's conduct and emotional problems [9] and parent-related stress [10]. However, access to face-to-face parenting support interventions was severely limited during the lockdowns. To circumvent this obstacle and reverse pandemic-related increases in children's conduct and emotional problems, we developed Parent Positive—a smartphone app providing evidence-based information and support to the general population."

2b) In INTRODUCTION: Specific objectives or hypotheses

Does your paper address CONSORT subitem 2b? *

Copy and paste relevant sections from the manuscript (include quotes in quotation marks "like this" to indicate direct quotes from your manuscript), or elaborate on this item by providing additional information not in the ms, or briefly explain why the item is not applicable/relevant for your study

"In Supporting Parents And Kids Through Lockdown Experiences (SPARKLE), using a randomized controlled trial (RCT) comparison of Parent Positive versus Follow-up As Usual (FAU) implemented within the Co-SPACE cohort, we set out to test the preregistered hypotheses [15] that (1) at 1 month after randomization, Parent Positive would reduce parent-reported child conduct (primary outcome) and emotional problems as well as parents' psychological distress and child-related worries and family conflict more generally, effects that would persist for 2 months after randomization; (2) reductions in problems would be greater for children with more preexisting conduct problems and higher levels of app use; and (3) Parent Positive would be cost-effective compared with FAU."

METHODS

3a) Description of trial design (such as parallel, factorial) including allocation ratio

Does your paper address CONSORT subitem 3a? *

Copy and paste relevant sections from the manuscript (include quotes in quotation marks "like this" to indicate direct quotes from your manuscript), or elaborate on this item by providing additional information not in the ms, or briefly explain why the item is not applicable/relevant for your study

"SPARKLE was a rapid-deployment 2-arm superiority parallel group RCT of Parent Positive versus FAU embedded in the Co-SPACE study (ie, a trial within a cohort). The published protocol is available here [15]."
"Participants were allocated to the study arm on a 1:1 ratio by simple randomization automatically after baseline through the Randomizer function within Qualtrics (Qualtrics International Inc). The app was downloadable from app stores, with access controlled via a study ID. No sampling blocking or stratification was undertaken. It was not possible to “blind” participants. Senior members (including SB and KG) of the research team remained blind until after the first draft of the analysis report was complete. Staff involved in data collection (MP, OR, and MK), the trial statistician (NBH), and the health economist (JS) were unblinded throughout."

3b) Important changes to methods after trial commencement (such as eligibility criteria), with reasons

Does your paper address CONSORT subitem 3b? *

Copy and paste relevant sections from the manuscript (include quotes in quotation marks "like this" to indicate direct quotes from your manuscript), or elaborate on this item by providing additional information not in the ms, or briefly explain why the item is not applicable/relevant for your study

No changes were made after trial commencement.

3b-i) Bug fixes, Downtimes, Content Changes

Bug fixes, Downtimes, Content Changes: ehealth systems are often dynamic systems. A description of changes to methods therefore also includes important changes made on the intervention or comparator during the trial (e.g., major bug fixes or changes in the functionality or content) (5-iii) and other “unexpected events” that may have influenced study design such as staff changes, system failures/downtimes, etc. [2].

subitem not at all important

1 ○

2 ○

3 ○

4 ○

5 ○

essential

Does your paper address subitem 3b-i?

Copy and paste relevant sections from the manuscript (include quotes in quotation marks "like this" to indicate direct quotes from your manuscript), or elaborate on this item by providing additional information not in the ms, or briefly explain why the item is not applicable/relevant for your study

Your answer

4a) Eligibility criteria for participants

Does your paper address CONSORT subitem 4a? *

Copy and paste relevant sections from the manuscript (include quotes in quotation marks "like this" to indicate direct quotes from your manuscript), or elaborate on this item by providing additional information not in the ms, or briefly explain why the item is not applicable/relevant for your study

"Participants were parents or carers of children aged 4 to 10 years. The inclusion criterion for Co-SPACE was parents aged ≥18 years residing in the United Kingdom with a child aged 4 to 16 years. For SPARKLE, only families with children aged 4 to 10 years and access to a compatible smartphone were included. Recruitment took place on the web via email newsletters; parent networks, support organizations, and charities; schools; children’s services departments; and media announcements. Parents of children aged 4 to 10 years who were already in Co-SPACE when SPARKLE started were invited to take part, whereas others could join Co-SPACE and SPARKLE after the trial started. Parents provided separate written informed consent for Co-SPACE and

4a-i) Computer / Internet literacy

Computer / Internet literacy is often an implicit “de facto” eligibility criterion - this should be explicitly clarified.

subitem not at all important

1 ○

2 ○

3 ○

4 ○

5 ○

essential

Does your paper address subitem 4a-i?

Copy and paste relevant sections from the manuscript (include quotes in quotation marks "like this" to indicate direct quotes from your manuscript), or elaborate on this item by providing additional information not in the ms, or briefly explain why the item is not applicable/relevant for your study

Your answer

4a-ii) Open vs. closed, web-based vs. face-to-face assessments:

Open vs. closed, web-based vs. face-to-face assessments: Mention how participants were recruited (online vs. offline), e.g., from an open access website or from a clinic, and clarify if this was a purely web-based trial, or there were face-to-face components (as part of the intervention or for assessment), i.e., to what degree got the study team to know the participant. In online-only trials, clarify if participants were quasi-anonymous and whether having multiple identities was possible or whether technical or logistical measures (e.g., cookies, email confirmation, phone calls) were used to detect/prevent these.

subitem not at all important

1 ○

2 ○

3 ○

4 ○

5 ○

essential

Does your paper address subitem 4a-ii? *

Copy and paste relevant sections from the manuscript (include quotes in quotation marks "like this" to indicate direct quotes from your manuscript), or elaborate on this item by providing additional information not in the ms, or briefly explain why the item is not applicable/relevant for your study

"Measures were administered at baseline (T1) and 1 (T2) and 2 (T3) months after randomization through Qualtrics as part of Co-SPACE data collection. Parent Positive use data were collected passively through Amazon Web Services. Raw data from the SPARKLE trial will be deposited in the UK Data Service repository [43]. Each participant provided written consent on the web and received a 2 × £5 (The conversion rate is £1 [US $1.26]) web-based shopping voucher to thank them for their time completing each follow-up questionnaire. Each participant was assigned a unique study ID that was used to link the deidentified questionnaire and app use data. A data privacy notice was available on the study website."

4a-iii) Information giving during recruitment

Information given during recruitment. Specify how participants were briefed for recruitment and in the informed consent procedures (e.g., publish the informed consent documentation as appendix, see also item X26), as this information may have an effect on user self-selection, user expectation and may also bias results.

subitem not at all important

1 ○

2 ○

3 ○

4 ○

5 ○

essential

Does your paper address subitem 4a-iii?

Copy and paste relevant sections from the manuscript (include quotes in quotation marks "like this" to indicate direct quotes from your manuscript), or elaborate on this item by providing additional information not in the ms, or briefly explain why the item is not applicable/relevant for your study

Your answer

4b) Settings and locations where the data were collected

Does your paper address CONSORT subitem 4b? *

Copy and paste relevant sections from the manuscript (include quotes in quotation marks "like this" to indicate direct quotes from your manuscript), or elaborate on this item by providing additional information not in the ms, or briefly explain why the item is not applicable/relevant for your study

"Measures were administered at baseline (T1) and 1 (T2) and 2 (T3) months after randomization through Qualtrics as part of Co-SPACE data collection. Parent Positive use data were collected passively through Amazon Web Services. Raw data from the SPARKLE trial will be deposited in the UK Data Service repository [43]. Each participant provided written consent on the web and received a 2 × £5 (The conversion rate is £1 [US $1.26]) web-based shopping voucher to thank them for their time completing each follow-up questionnaire. Each participant was assigned a unique study ID that was used to link the deidentified questionnaire and app use data. A data privacy notice was available on the study website."

4b-i) Report if outcomes were (self-)assessed through online questionnaires

Clearly report if outcomes were (self-)assessed through online questionnaires (as common in web-based trials) or otherwise.

subitem not at all important

1 ○

2 ○

3 ○

4 ○

5 ○

essential

Does your paper address subitem 4b-i? *

Copy and paste relevant sections from the manuscript (include quotes in quotation marks "like this" to indicate direct quotes from your manuscript), or elaborate on this item by providing additional information not in the ms, or briefly explain why the item is not applicable/relevant for your study

"Measures were administered at baseline (T1) and 1 (T2) and 2 (T3) months after randomization through Qualtrics as part of Co-SPACE data collection. Parent Positive use data were collected passively through Amazon Web Services. Raw data from the SPARKLE trial will be deposited in the UK Data Service repository [43]. Each participant provided written consent on the web and received a 2 × £5 (The conversion rate is £1 [US $1.26]) web-based shopping voucher to thank them for their time completing each follow-up questionnaire. Each participant was assigned a unique study ID that was used to link the deidentified questionnaire and app use data. A data privacy notice was available on the study website."

4b-ii) Report how institutional affiliations are displayed

Report how institutional affiliations are displayed to potential participants [on ehealth media], as affiliations with prestigious hospitals or universities may affect volunteer rates, use, and reactions with regards to an intervention.(Not a required item – describe only if this may bias results)

subitem not at all important

1 ○

2 ○

3 ○

4 ○

5 ○

essential

Does your paper address subitem 4b-ii?

Copy and paste relevant sections from the manuscript (include quotes in quotation marks "like this" to indicate direct quotes from your manuscript), or elaborate on this item by providing additional information not in the ms, or briefly explain why the item is not applicable/relevant for your study

Your answer

5) The interventions for each group with sufficient details to allow replication, including how and when they were actually administered

5-i) Mention names, credential, affiliations of the developers, sponsors, and owners

Mention names, credential, affiliations of the developers, sponsors, and owners [6] (if authors/evaluators are owners or developer of the software, this needs to be declared in a “Conflict of interest” section or mentioned elsewhere in the manuscript).

subitem not at all important

1 ○

2 ○

3 ○

4 ○

5 ○

essential

Does your paper address subitem 5-i?

Copy and paste relevant sections from the manuscript (include quotes in quotation marks "like this" to indicate direct quotes from your manuscript), or elaborate on this item by providing additional information not in the ms, or briefly explain why the item is not applicable/relevant for your study

Your answer

5-ii) Describe the history/development process

Describe the history/development process of the application and previous formative evaluations (e.g., focus groups, usability testing), as these will have an impact on adoption/use rates and help with interpreting results.

subitem not at all important

1 ○

2 ○

3 ○

4 ○

5 ○

essential

Does your paper address subitem 5-ii?

Copy and paste relevant sections from the manuscript (include quotes in quotation marks "like this" to indicate direct quotes from your manuscript), or elaborate on this item by providing additional information not in the ms, or briefly explain why the item is not applicable/relevant for your study

Your answer

5-iii) Revisions and updating

Revisions and updating. Clearly mention the date and/or version number of the application/intervention (and comparator, if applicable) evaluated, or describe whether the intervention underwent major changes during the evaluation process, or whether the development and/or content was “frozen” during the trial. Describe dynamic components such as news feeds or changing content which may have an impact on the replicability of the intervention (for unexpected events see item 3b).

subitem not at all important

1 ○

2 ○

3 ○

4 ○

5 ○

essential

Does your paper address subitem 5-iii?

Copy and paste relevant sections from the manuscript (include quotes in quotation marks "like this" to indicate direct quotes from your manuscript), or elaborate on this item by providing additional information not in the ms, or briefly explain why the item is not applicable/relevant for your study

Your answer

5-iv) Quality assurance methods

Provide information on quality assurance methods to ensure accuracy and quality of information provided [1], if applicable.

subitem not at all important

1 ○

2 ○

3 ○

4 ○

5 ○

essential

Does your paper address subitem 5-iv?

Copy and paste relevant sections from the manuscript (include quotes in quotation marks "like this" to indicate direct quotes from your manuscript), or elaborate on this item by providing additional information not in the ms, or briefly explain why the item is not applicable/relevant for your study

Your answer

5-v) Ensure replicability by publishing the source code, and/or providing screenshots/screen-capture video, and/or providing flowcharts of the algorithms used

Ensure replicability by publishing the source code, and/or providing screenshots/screen-capture video, and/or providing flowcharts of the algorithms used. Replicability (i.e., other researchers should in principle be able to replicate the study) is a hallmark of scientific reporting.

subitem not at all important

1 ○

2 ○

3 ○

4 ○

5 ○

essential

Does your paper address subitem 5-v?

Copy and paste relevant sections from the manuscript (include quotes in quotation marks "like this" to indicate direct quotes from your manuscript), or elaborate on this item by providing additional information not in the ms, or briefly explain why the item is not applicable/relevant for your study

Your answer

5-vi) Digital preservation

Digital preservation: Provide the URL of the application, but as the intervention is likely to change or disappear over the course of the years; also make sure the intervention is archived (Internet Archive, webcitation.org, and/or publishing the source code or screenshots/videos alongside the article). As pages behind login screens cannot be archived, consider creating demo pages which are accessible without login.

subitem not at all important

1 ○

2 ○

3 ○

4 ○

5 ○

essential

Does your paper address subitem 5-vi?

Copy and paste relevant sections from the manuscript (include quotes in quotation marks "like this" to indicate direct quotes from your manuscript), or elaborate on this item by providing additional information not in the ms, or briefly explain why the item is not applicable/relevant for your study

Your answer

5-vii) Access

Access: Describe how participants accessed the application, in what setting/context, if they had to pay (or were paid) or not, whether they had to be a member of specific group. If known, describe how participants obtained “access to the platform and Internet” [1]. To ensure access for editors/reviewers/readers, consider to provide a “backdoor” login account or demo mode for reviewers/readers to explore the application (also important for archiving purposes, see vi).

subitem not at all important

1 ○

2 ○

3 ○

4 ○

5 ○

essential

Does your paper address subitem 5-vii? *

Copy and paste relevant sections from the manuscript (include quotes in quotation marks "like this" to indicate direct quotes from your manuscript), or elaborate on this item by providing additional information not in the ms, or briefly explain why the item is not applicable/relevant for your study

"Participants were allocated to the study arm on a 1:1 ratio by simple randomization automatically after baseline through the Randomizer function within Qualtrics (Qualtrics International Inc). The app was downloadable from app stores, with access controlled via a study ID. No sampling blocking or stratification was undertaken."

5-viii) Mode of delivery, features/functionalities/components of the intervention and comparator, and the theoretical framework

Describe mode of delivery, features/functionalities/components of the intervention and comparator, and the theoretical framework [6] used to design them (instructional strategy [1], behaviour change techniques, persuasive features, etc., see e.g., [7, 8] for terminology). This includes an in-depth description of the content (including where it is coming from and who developed it) [1]," whether [and how] it is tailored to individual circumstances and allows users to track their progress and receive feedback" [6]. This also includes a description of communication delivery channels and – if computer-mediated communication is a component – whether communication was synchronous or asynchronous [6]. It also includes information on presentation strategies [1], including page design principles, average amount of text on pages, presence of hyperlinks to other resources, etc. [1].

subitem not at all important

1 ○

2 ○

3 ○

4 ○

5 ○

essential

Does your paper address subitem 5-viii? *

Copy and paste relevant sections from the manuscript (include quotes in quotation marks "like this" to indicate direct quotes from your manuscript), or elaborate on this item by providing additional information not in the ms, or briefly explain why the item is not applicable/relevant for your study

"The Parent Positive app delivers universal parenting information and peer support based on >20 years of evidence from parent training RCTs [11]. As described in the Introduction section, it was designed specifically to help parents during the COVID-19 pandemic. It has 3 zones. The Parenting Boosters provided advice on 8 common parenting challenges organized around key themes. The boosters were entitled (1) “Staying positive and motivated,” (2) “Making sure everyone knows what is expected of them,” (3) “Building your child’s self-confidence and trust,” (4) “Getting your child to follow instructions,” (5) “Promoting better behaviour,” (6) “Limiting conflict,” (7) “Keeping calm when your kids act up,” and (8) “Careful use of sanctions.” These are complemented by a range of digital material aligned with their themes derived from the EPEC parenting program [12,13], and from the STEPS app [14]. The Parenting Exchange provided a platform for parent-to-parent communication facilitated by 6 experienced, trained [13] EPEC parent group leaders who moderated content and responded where appropriate to parent posts. The moderators were paid for their role and received monthly 1-hour group supervision. Supervision consolidated parent moderators’ existing skills and knowledge and adapted these for use within their moderator role in the Parenting Exchange. The Parenting Exchange also provided opportunities for parents to submit questions to academic and clinical child development and parenting experts, which were then addressed in 9 Ask the Expert videos. These included 4 sessions on parenting and behavior management and 1 session each on anxiety, education and learning, neurodevelopmental problems, sleep, and obsessive-compulsive disorder. The third zone, Parenting Resources, provided links to carefully selected high-quality web-

5-ix) Describe use parameters

Describe use parameters (e.g., intended “doses” and optimal timing for use). Clarify what instructions or recommendations were given to the user, e.g., regarding timing, frequency, heaviness of use, if any, or was the intervention used ad libitum.

subitem not at all important

1 ○

2 ○

3 ○

4 ○

5 ○

essential

Does your paper address subitem 5-ix?

Copy and paste relevant sections from the manuscript (include quotes in quotation marks "like this" to indicate direct quotes from your manuscript), or elaborate on this item by providing additional information not in the ms, or briefly explain why the item is not applicable/relevant for your study

Your answer

5-x) Clarify the level of human involvement

Clarify the level of human involvement (care providers or health professionals, also technical assistance) in the e-intervention or as co-intervention (detail number and expertise of professionals involved, if any, as well as “type of assistance offered, the timing and frequency of the support, how it is initiated, and the medium by which the assistance is delivered”. It may be necessary to distinguish between the level of human involvement required for the trial, and the level of human involvement required for a routine application outside of a RCT setting (discuss under item 21 – generalizability).

subitem not at all important

1

2

3

4

5

essential

Does your paper address subitem 5-x?

Copy and paste relevant sections from the manuscript (include quotes in quotation marks "like this" to indicate direct quotes from your manuscript), or elaborate on this item by providing additional information not in the ms, or briefly explain why the item is not applicable/relevant for your study

Your answer

5-xi) Report any prompts/reminders used

Report any prompts/reminders used: Clarify if there were prompts (letters, emails, phone calls, SMS) to use the application, what triggered them, frequency etc. It may be necessary to distinguish between the level of prompts/reminders required for the trial, and the level of prompts/reminders for a routine application outside of a RCT setting (discuss under item 21 – generalizability).

subitem not at all important

1 ◯

2 ◯

3 ◯

4 ◯

5 ◯

essential

Does your paper address subitem 5-xi? *

Copy and paste relevant sections from the manuscript (include quotes in quotation marks "like this" to indicate direct quotes from your manuscript), or elaborate on this item by providing additional information not in the ms, or briefly explain why the item is not applicable/relevant for your study

"The Parenting Exchange provided a platform for parent-to-parent communication facilitated by 6 experienced, trained [13] EPEC parent group leaders who moderated content and responded where appropriate to parent posts. The moderators were paid for their role and received monthly 1-hour group supervision. Supervision consolidated parent moderators' existing skills and knowledge and adapted these for use within their moderator role in the Parenting Exchange. The Parenting Exchange also provided opportunities for parents to submit questions to academic and clinical child development and parenting experts, which were then addressed in 9 Ask the Expert videos. These included 4 sessions on parenting and behavior management and 1 session each on anxiety, education and learning, neurodevelopmental problems, sleep, and obsessive-compulsive disorder."

Reminders about the Ask the Expert sessions were sent to the parents directly through emails and app-notifications were received if someone posted a comment on the Exchange.

5-xii) Describe any co-interventions (incl. training/support)

Describe any co-interventions (incl. training/support): Clearly state any interventions that are provided in addition to the targeted eHealth intervention, as ehealth intervention may not be designed as stand-alone intervention. This includes training sessions and support [1]. It may be necessary to distinguish between the level of training required for the trial, and the level of training for a routine application outside of a RCT setting (discuss under item 21 – generalizability.

subitem not at all important

1 ◯

2 ◯

3 ◯

4 ◯

5 ◯

essential

Does your paper address subitem 5-xii? *

Copy and paste relevant sections from the manuscript (include quotes in quotation marks "like this" to indicate direct quotes from your manuscript), or elaborate on this item by providing additional information not in the ms, or briefly explain why the item is not applicable/relevant for your study

"The Parenting Exchange provided a platform for parent-to-parent communication facilitated by 6 experienced, trained [13] EPEC parent group leaders who moderated content and responded where appropriate to parent posts. The moderators were paid for their role and received monthly 1-hour group supervision. Supervision consolidated parent moderators' existing skills and knowledge and adapted these for use within their moderator role in the Parenting Exchange. The Parenting Exchange also provided opportunities for parents to submit questions to academic and clinical child development and parenting experts, which were then addressed in 9 Ask the Expert videos. These included 4 sessions on parenting and behavior management and 1 session each on anxiety, education and learning, neurodevelopmental problems, sleep, and obsessive-compulsive disorder."

6a) Completely defined pre-specified primary and secondary outcome measures, including how and when they were assessed

Does your paper address CONSORT subitem 6a? *

Copy and paste relevant sections from the manuscript (include quotes in quotation marks "like this" to indicate direct quotes from your manuscript), or elaborate on this item by providing additional information not in the ms, or briefly explain why the item is not applicable/relevant for your study

"Clinical Outcomes
The primary outcome was parent-reported child conduct problems using the conduct subscale of the Strengths and Difficulties Questionnaire (SDQ) [16] at 1 month after randomization (T2). This is a validated 5-item subscale measuring children's oppositionality, defiance, and disruptive behavior rated on 3-point Likert scales (not true, somewhat true, and certainly true). An overall subscale score, with higher scores reflecting more problematic behavior, was derived.
Parent-reported SDQ conduct problems score at 2 months after randomization (T3) was a secondary outcome. Other validated secondary outcomes were T2 and T3 measures of (1) parent-reported child emotional problems measured on the SDQ 5-item emotional symptoms subscale and (2) parental psychological distress measured on the Depression, Anxiety, and Stress Scale (DASS)–21 [17] and multiplied to form a DASS-42 score [15]. In addition, 2 scales used in Co-SPACE measured (1) child-related parent worries about their behavior, well-being, screen time use, education, and the future (previously, Cronbach α has been >.54, and test-retest reliability was r=0.73) and (2) family conflict related to arguments between parents, parents and children, and siblings as secondary outcomes at both time points [18]. In both cases, a single scale score was created—higher scores indicate more stress and worries and greater family conflict.
Health Economic Measures
Retrospective child-related use of health and social care services, including those provided in schools, was measured at T2 and T3 using a modified version of the Child and Adolescent Service Use Schedule (CA-SUS) [19]. Unit costs for the financial year 2020-2021 were applied to calculate the total cost of resources used by each participant over the periods from T1 to T2 and T1 or T2 (dependent on T2 completion of data collection) to T3 (Table S1 in Multimedia Appendix 1 [38-42]). Parent Positive costs included app development and maintenance costs plus time costs associated with the Parenting Exchange operations (full details are provided in Multimedia Appendix 1).
Economic outcomes were quality-adjusted life years (QALYs) generated from the 25-item parent-reported SDQ using a crosswalk algorithm to map scores onto the Child Health Utility–9 Dimensions (CHU9D), a pediatric health–related quality-of-life measure [20]. The CHU9D [21] consists of 9 dimensions (sad, worried, pain, annoyed, tired, homework or schoolwork, daily routine, activities, and sleep) rated using 5 levels [20]. QALYs were calculated using the area under the curve approach, which assumed that utility score changes follow a linear path [22]. One QALY is equivalent to 1 year in perfect health. When a child's quality of life is less than perfect, a QALY can be calculated by multiplying the survival time in the impaired state by the corresponding CHU9D utility value.
Intervention Use and Satisfaction
The main Parent Positive use measure was total time spent accessing information within each booster by T2 and T3. Other use metrics included the number of times Parent Positive was accessed and the number of posts and responses to posts on the Parenting Exchange. Satisfaction with Parent Positive was measured using a questionnaire developed for SPARKLE based on the average of 3 items measuring the usefulness of each zone rated on a Likert scale from 0 to 6 (not at all to very useful). Higher scores indicated greater usefulness.
Other Measures
Family characteristics and demographic information were collected at baseline. Data on lockdown-related circumstances (eg, being in lockdown) were collected. Adverse events (AEs) were measured using a questionnaire developed for SPARKLE that asked parents to report negative events related to their child's and their own physical and mental health problems and their relationships or daily activities. The information was coded to

6a-i) Online questionnaires: describe if they were validated for online use and apply CHERRIES items to describe how the questionnaires were designed/deployed

If outcomes were obtained through online questionnaires, describe if they were validated for online use and apply CHERRIES items to describe how the questionnaires were designed/deployed [9].

subitem not at all important

1 ○

2 ○

3 ○

4 ○

5 ○

essential

Does your paper address subitem 6a-i?

Copy and paste relevant sections from manuscript text

Your answer

6a-ii) Describe whether and how “use” (including intensity of use/dosage) was defined/measured/monitored

Describe whether and how “use” (including intensity of use/dosage) was defined/measured/monitored (logins, logfile analysis, etc.). Use/adoption metrics are important process outcomes that should be reported in any ehealth trial.

subitem not at all important

1 ○

2 ○

3 ○

4 ○

5 ○

essential

Does your paper address subitem 6a-ii?

Copy and paste relevant sections from manuscript text

Your answer

6a-iii) Describe whether, how, and when qualitative feedback from participants was obtained

Describe whether, how, and when qualitative feedback from participants was obtained (e.g., through emails, feedback forms, interviews, focus groups).

subitem not at all important

1 ○

2 ○

3 ○

4 ○

5 ○

essential

Does your paper address subitem 6a-iii?

Copy and paste relevant sections from manuscript text

Your answer

6b) Any changes to trial outcomes after the trial commenced, with reasons

Does your paper address CONSORT subitem 6b? *

Copy and paste relevant sections from the manuscript (include quotes in quotation marks "like this" to indicate direct quotes from your manuscript), or elaborate on this item by providing additional information not in the ms, or briefly explain why the item is not applicable/relevant for your study

There were not changes to the outcomes after commencement of the trial.

7a) How sample size was determined

NPT: When applicable, details of whether and how the clustering by care provides or centers was addressed

7a-i) Describe whether and how expected attrition was taken into account when calculating the sample size

Describe whether and how expected attrition was taken into account when calculating the sample size.

subitem not at all important

1 ○

2 ○

3 ○

4 ○

5 ○

essential

Does your paper address subitem 7a-i?

Copy and paste relevant sections from manuscript title (include quotes in quotation marks "like this" to indicate direct quotes from your manuscript), or elaborate on this item by providing additional information not in the ms, or briefly explain why the item is not applicable/relevant for your study

Your answer

7b) When applicable, explanation of any interim analyses and stopping guidelines

Does your paper address CONSORT subitem 7b? *

Copy and paste relevant sections from the manuscript (include quotes in quotation marks "like this" to indicate direct quotes from your manuscript), or elaborate on this item by providing additional information not in the ms, or briefly explain why the item is not applicable/relevant for your study

"The independent TSC, consisting of clinicians, statisticians, health economists, policy makers, and Patient and Public Involvement representatives, met every 4 months. No interim analyses were undertaken. The role of the Data Monitoring and Ethics Committee was taken over by the TSC."

8a) Method used to generate the random allocation sequence

NPT: When applicable, how care providers were allocated to each trial group

Does your paper address CONSORT subitem 8a? *

Copy and paste relevant sections from the manuscript (include quotes in quotation marks "like this" to indicate direct quotes from your manuscript), or elaborate on this item by providing additional information not in the ms, or briefly explain why the item is not applicable/relevant for your study

"Participants were allocated to the study arm on a 1:1 ratio by simple randomization automatically after baseline through the Randomizer function within Qualtrics (Qualtrics International Inc). The app was downloadable from app stores, with access controlled via a study ID. No sampling blocking or stratification was undertaken. It was not possible to “blind” participants. Senior members (including SB and KG) of the research team remained blind until after the first draft of the analysis report was complete. Staff involved in data collection (MP, OR, and MK), the trial statistician (NBH), and the health economist (JS) were unblinded throughout."

8b) Type of randomisation; details of any restriction (such as blocking and block size)

Does your paper address CONSORT subitem 8b? *

Copy and paste relevant sections from the manuscript (include quotes in quotation marks "like this" to indicate direct quotes from your manuscript), or elaborate on this item by providing additional information not in the ms, or briefly explain why the item is not applicable/relevant for your study

"Participants were allocated to the study arm on a 1:1 ratio by simple randomization automatically after baseline through the Randomizer function within Qualtrics (Qualtrics International Inc). The app was downloadable from app stores, with access controlled via a study ID. No sampling blocking or stratification was undertaken. It was not possible to "blind" participants. Senior members (including SB and KG) of the research team remained blind until after the first draft of the analysis report was complete. Staff involved in data collection (MP, OR, and MK), the trial statistician (NBH), and the health economist (JS) were unblinded throughout."

9) Mechanism used to implement the random allocation sequence (such as sequentially numbered containers), describing any steps taken to conceal the sequence until interventions were assigned

Does your paper address CONSORT subitem 9? *

Copy and paste relevant sections from the manuscript (include quotes in quotation marks "like this" to indicate direct quotes from your manuscript), or elaborate on this item by providing additional information not in the ms, or briefly explain why the item is not applicable/relevant for your study

"Participants were allocated to the study arm on a 1:1 ratio by simple randomization automatically after baseline through the Randomizer function within Qualtrics (Qualtrics International Inc). The app was downloadable from app stores, with access controlled via a study ID. No sampling blocking or stratification was undertaken. It was not possible to "blind" participants. Senior members (including SB and KG) of the research team remained blind until after the first draft of the analysis report was complete. Staff involved in data collection (MP, OR, and MK), the trial statistician (NBH), and the health economist (JS) were unblinded throughout."

10) Who generated the random allocation sequence, who enrolled participants, and who assigned participants to interventions

Does your paper address CONSORT subitem 10? *

Copy and paste relevant sections from the manuscript (include quotes in quotation marks "like this" to indicate direct quotes from your manuscript), or elaborate on this item by providing additional information not in the ms, or briefly explain why the item is not applicable/relevant for your study

"Participants were allocated to the study arm on a 1:1 ratio by simple randomization automatically after baseline through the Randomizer function within Qualtrics (Qualtrics International Inc). The app was downloadable from app stores, with access controlled via a study ID. No sampling blocking or stratification was undertaken. It was not possible to “blind” participants. Senior members (including SB and KG) of the research team remained blind until after the first draft of the analysis report was complete. Staff involved in data collection (MP, OR, and MK), the trial statistician (NBH), and the health economist (JS) were unblinded throughout."

11a) If done, who was blinded after assignment to interventions (for example, participants, care providers, those assessing outcomes) and how

NPT: Whether or not administering co-interventions were blinded to group assignment

11a-i) Specify who was blinded, and who wasn’t

Specify who was blinded, and who wasn’t. Usually, in web-based trials it is not possible to blind the participants [1, 3] (this should be clearly acknowledged), but it may be possible to blind outcome assessors, those doing data analysis or those administering co-interventions (if any).

subitem not at all important

1 ○

2 ○

3 ○

4 ○

5 ○

essential

Does your paper address subitem 11a-i? *

Copy and paste relevant sections from the manuscript (include quotes in quotation marks "like this" to indicate direct quotes from your manuscript), or elaborate on this item by providing additional information not in the ms, or briefly explain why the item is not applicable/relevant for your study

"Participants were allocated to the study arm on a 1:1 ratio by simple randomization automatically after baseline through the Randomizer function within Qualtrics (Qualtrics International Inc). The app was downloadable from app stores, with access controlled via a study ID. No sampling blocking or stratification was undertaken. It was not possible to “blind” participants. Senior members (including SB and KG) of the research team remained blind until after the first draft of the analysis report was complete. Staff involved in data collection (MP, OR, and MK), the trial statistician (NBH), and the health economist (JS) were unblinded throughout."

11a-ii) Discuss e.g., whether participants knew which intervention was the “intervention of interest” and which one was the “comparator”

Informed consent procedures (4a-ii) can create biases and certain expectations - discuss e.g., whether participants knew which intervention was the “intervention of interest” and which one was the “comparator”.

subitem not at all important

1 ○

2 ○

3 ○

4 ○

5 ○

essential

Does your paper address subitem 11a-ii?

Copy and paste relevant sections from the manuscript (include quotes in quotation marks "like this" to indicate direct quotes from your manuscript), or elaborate on this item by providing additional information not in the ms, or briefly explain why the item is not applicable/relevant for your study

Your answer

11b) If relevant, description of the similarity of interventions

(this item is usually not relevant for ehealth trials as it refers to similarity of a placebo or sham intervention to a active medication/intervention)

Does your paper address CONSORT subitem 11b? *

Copy and paste relevant sections from the manuscript (include quotes in quotation marks "like this" to indicate direct quotes from your manuscript), or elaborate on this item by providing additional information not in the ms, or briefly explain why the item is not applicable/relevant for your study

Not applicable for this trial.

12a) Statistical methods used to compare groups for primary and secondary outcomes

NPT: When applicable, details of whether and how the clustering by care providers or centers was addressed

Does your paper address CONSORT subitem 12a? *

Copy and paste relevant sections from the manuscript (include quotes in quotation marks "like this" to indicate direct quotes from your manuscript), or elaborate on this item by providing additional information not in the ms, or briefly explain why the item is not applicable/relevant for your study

"Analysis
The SPARKLE Statistical Analysis Plan and Health Economics Analysis Plan can be accessed on the web [44].
Outcome Analysis
All analyses were carried out using Stata (version 17.0; StataCorp). Baseline and postrandomization app use variables were described by intervention arm and overall, with categorical variables described using frequencies and proportions and continuous variables described using mean and SD or median and IQR as appropriate. App usage between T1 to T2 and T1 to T3 was reported, instead of T1 to T2 and T2 to T3 as specified in the SAP. Differences between arms were assessed using mixed-effects linear mixed models (LMMs) analysis of covariance models, with T2 and T3 measures as dependent variables and a random intercept at the participant level. All models included age, gender (both prespecified), T1 outcomes, intervention arm, time, intervention arm by time interaction, and any additional baseline variables found to be predictive of missingness as fixed effects. Marginal mean Parent Positive versus FAU differences at T2 and T3 were extracted from the models, with associated 1-sided 95% CIs and P values and a 1-sided type-1 error rate of 5% in favor of Parent Positive. See Multimedia Appendix 1 for 2-sided P values (as specified in the Statistical Analysis Plan).
Missing outcome data were summarized; those with at least 1 postrandomization value were included in the LMM models under the intention-to-treat principle. Baseline variables that predicted (at $P<.05$) a binary missingness of any of the primary or secondary outcome variables within a multivariable logistic regression model (with covariates listed for the aforementioned primary analysis) were also included as covariates to make the missing-at-random assumption more plausible and, thus, increase the validity of our intention-to-treat estimate under this assumption [24]. Mean imputation was used for missing data in baseline covariates [25]. Complier average causal effect estimates for those using the app were also conducted (Multimedia Appendix 1). The moderating effect of T1 conduct problems on conduct problem intervention effects at T2 and T3 was assessed by adding a time-by-treatment-by-baseline conduct interaction term to the LMM analysis of covariance model for this outcome. Assessing moderation by postrandomization app use by principal stratification models was not possible as we did not have sufficiently strong predictors of app use [26].
Medical (physical and psychological) and nonmedical familial AEs (eg, reduction in school attendance) and serious AEs were summarized.
Economic Analysis
Between-arm differences in costs and QALYs were analyzed using generalized linear models with bootstrapped 95% CIs adjusted for prespecified T1 covariates and CHU9D scores. Adjustment for differences in T1 costs was not possible as service use data were not collected in Co-SPACE; the CA-SUS was administered at T2 and T3 only. The primary economic analysis was a cost-utility analysis at T2. Costs were calculated from the National Health Service and personal social services perspective. Effects were measured in terms of QALYs. Incremental cost-effectiveness ratios were calculated, and nonparametric bootstrapping was used to propagate sampling uncertainty surrounding the mean incremental cost-effectiveness ratios by generating 1000 estimates of incremental costs and QALYs [27]. Cost-effectiveness acceptability curves demonstrated the probability that Parent Positive was cost-effective compared with FAU using the National Institute for Health and Care Excellence cost-effectiveness threshold of £20,000 to £30,000 per QALY [28]. Following standard practice, outliers with costs in the 99th percentile [29] were excluded from the analysis on the grounds that they may distort cost-effectiveness findings given that it was not possible to adjust for baseline

distort cost effectiveness findings given that it was not possible to adjust for baseline differences in costs. Sensitivity analyses explored the effect of missing cost and QALY data (using multiple imputation with chained equations) [30] and the effect of replacing

12a-i) Imputation techniques to deal with attrition / missing values

Imputation techniques to deal with attrition / missing values: Not all participants will use the intervention/comparator as intended and attrition is typically high in ehealth trials. Specify how participants who did not use the application or dropped out from the trial were treated in the statistical analysis (a complete case analysis is strongly discouraged, and simple imputation techniques such as LOCF may also be problematic [4]).

subitem not at all important

1 ○

2 ○

3 ○

4 ○

5 ○

essential

Does your paper address subitem 12a-i? *

Copy and paste relevant sections from the manuscript (include quotes in quotation marks "like this" to indicate direct quotes from your manuscript), or elaborate on this item by providing additional information not in the ms, or briefly explain why the item is not applicable/relevant for your study

"Analysis
The SPARKLE Statistical Analysis Plan and Health Economics Analysis Plan can be accessed on the web [44].
Outcome Analysis
All analyses were carried out using Stata (version 17.0; StataCorp). Baseline and postrandomization app use variables were described by intervention arm and overall, with categorical variables described using frequencies and proportions and continuous variables described using mean and SD or median and IQR as appropriate. App usage between T1 to T2 and T1 to T3 was reported, instead of T1 to T2 and T2 to T3 as specified in the SAP. Differences between arms were assessed using mixed-effects linear mixed models (LMMs) analysis of covariance models, with T2 and T3 measures as dependent variables and a random intercept at the participant level. All models included age, gender (both prespecified), T1 outcomes, intervention arm, time, intervention arm by time interaction, and any additional baseline variables found to be predictive of missingness as fixed effects. Marginal mean Parent Positive versus FAU differences at T2 and T3 were extracted from the models, with associated 1-sided 95% CIs and P values and a 1-sided type-1 error rate of 5% in favor of Parent Positive. See Multimedia Appendix 1 for 2-sided P values (as specified in the Statistical Analysis Plan).
Missing outcome data were summarized; those with at least 1 postrandomization value were included in the LMM models under the intention-to-treat principle. Baseline variables that predicted (at P<.05) a binary missingness of any of the primary or secondary outcome variables within a multivariable logistic regression model (with covariates listed for the aforementioned primary analysis) were also included as covariates to make the missing-at-random assumption more plausible and, thus, increase the validity of our intention-to-treat estimate under this assumption [24]. Mean imputation was used for missing data in baseline covariates [25]. Complier average causal effect estimates for those using the app were also conducted (Multimedia Appendix 1). The moderating effect of T1 conduct problems on conduct problem intervention effects at T2 and T3 was assessed by adding a time-by-treatment-by-baseline conduct interaction term to the LMM analysis of covariance model for this outcome. Assessing moderation by postrandomization app use by principal stratification models was not possible as we did not have sufficiently strong predictors of app use [26].
Medical (physical and psychological) and nonmedical familial AEs (eg, reduction in school attendance) and serious AEs were summarized.
Economic Analysis
Between-arm differences in costs and QALYs were analyzed using generalized linear models with bootstrapped 95% CIs adjusted for prespecified T1 covariates and CHU9D scores. Adjustment for differences in T1 costs was not possible as service use data were not collected in Co-SPACE; the CA-SUS was administered at T2 and T3 only. The primary economic analysis was a cost-utility analysis at T2. Costs were calculated from the National Health Service and personal social services perspective. Effects were measured in terms of QALYs. Incremental cost-effectiveness ratios were calculated, and nonparametric bootstrapping was used to propagate sampling uncertainty surrounding the mean incremental cost-effectiveness ratios by generating 1000 estimates of incremental costs and QALYs [27]. Cost-effectiveness acceptability curves demonstrated the probability that Parent Positive was cost-effective compared with FAU using the National Institute for Health and Care Excellence cost-effectiveness threshold of £20,000 to £30,000 per QALY [28]. Following standard practice, outliers with costs in the 99th percentile [29] were excluded from the analysis on the grounds that they may distort cost-effectiveness findings given that it was not possible to adjust for baseline

[illegible] differences in costs. Sensitivity analyses explored the effect of missing cost and QALY data (using multiple imputation with chained equations) [30] and the effect of replacing QALYs with the primary clinical outcome. A prespecified secondary economic analysis

12b) Methods for additional analyses, such as subgroup analyses and adjusted analyses

Does your paper address CONSORT subitem 12b? *

Copy and paste relevant sections from the manuscript (include quotes in quotation marks "like this" to indicate direct quotes from your manuscript), or elaborate on this item by providing additional information not in the ms, or briefly explain why the item is not applicable/relevant for your study

"Analysis
The SPARKLE Statistical Analysis Plan and Health Economics Analysis Plan can be accessed on the web [44].
Outcome Analysis
All analyses were carried out using Stata (version 17.0; StataCorp). Baseline and postrandomization app use variables were described by intervention arm and overall, with categorical variables described using frequencies and proportions and continuous variables described using mean and SD or median and IQR as appropriate. App usage between T1 to T2 and T1 to T3 was reported, instead of T1 to T2 and T2 to T3 as specified in the SAP. Differences between arms were assessed using mixed-effects linear mixed models (LMMs) analysis of covariance models, with T2 and T3 measures as dependent variables and a random intercept at the participant level. All models included age, gender (both prespecified), T1 outcomes, intervention arm, time, intervention arm by time interaction, and any additional baseline variables found to be predictive of missingness as fixed effects. Marginal mean Parent Positive versus FAU differences at T2 and T3 were extracted from the models, with associated 1-sided 95% CIs and P values and a 1-sided type-1 error rate of 5% in favor of Parent Positive. See Multimedia Appendix 1 for 2-sided P values (as specified in the Statistical Analysis Plan).
Missing outcome data were summarized; those with at least 1 postrandomization value were included in the LMM models under the intention-to-treat principle. Baseline variables that predicted (at P<.05) a binary missingness of any of the primary or secondary outcome variables within a multivariable logistic regression model (with covariates listed for the aforementioned primary analysis) were also included as covariates to make the missing-at-random assumption more plausible and, thus, increase the validity of our intention-to-treat estimate under this assumption [24]. Mean imputation was used for missing data in baseline covariates [25]. Complier average causal effect estimates for those using the app were also conducted (Multimedia Appendix 1). The moderating effect of T1 conduct problems on conduct problem intervention effects at T2 and T3 was assessed by adding a time-by-treatment-by-baseline conduct interaction term to the LMM analysis of covariance model for this outcome. Assessing moderation by postrandomization app use by principal stratification models was not possible as we did not have sufficiently strong predictors of app use [26].
Medical (physical and psychological) and nonmedical familial AEs (eg, reduction in school attendance) and serious AEs were summarized.
Economic Analysis
Between-arm differences in costs and QALYs were analyzed using generalized linear models with bootstrapped 95% CIs adjusted for prespecified T1 covariates and CHU9D scores. Adjustment for differences in T1 costs was not possible as service use data were not collected in Co-SPACE; the CA-SUS was administered at T2 and T3 only. The primary economic analysis was a cost-utility analysis at T2. Costs were calculated from the National Health Service and personal social services perspective. Effects were measured in terms of QALYs. Incremental cost-effectiveness ratios were calculated, and nonparametric bootstrapping was used to propagate sampling uncertainty surrounding the mean incremental cost-effectiveness ratios by generating 1000 estimates of incremental costs and QALYs [27]. Cost-effectiveness acceptability curves demonstrated the probability that Parent Positive was cost-effective compared with FAU using the National Institute for Health and Care Excellence cost-effectiveness threshold of £20,000 to £30,000 per QALY [28]. Following standard practice, outliers with costs in the 99th percentile [29] were excluded from the analysis on the grounds that they may distort cost-effectiveness findings given that it was not possible to adjust for baseline

[illegible]
differences in costs. Sensitivity analyses explored the effect of missing cost and QALY data (using multiple imputation with chained equations) [30] and the effect of replacing

X26) REB/IRB Approval and Ethical Considerations [recommended as subheading under "Methods"] (not a CONSORT item)

X26-i) Comment on ethics committee approval

subitem not at all important

1 ○

2 ○

3 ○

4 ○

5 ○

essential

Does your paper address subitem X26-i?

Copy and paste relevant sections from the manuscript (include quotes in quotation marks "like this" to indicate direct quotes from your manuscript), or elaborate on this item by providing additional information not in the ms, or briefly explain why the item is not applicable/relevant for your study

Your answer

x26-ii) Outline informed consent procedures

Outline informed consent procedures e.g., if consent was obtained offline or online (how? Checkbox, etc.?), and what information was provided (see 4a-ii). See [6] for some items to be included in informed consent documents.

subitem not at all important

1 ○

2 ○

3 ○

4 ○

5 ○

essential

Does your paper address subitem X26-ii?

Copy and paste relevant sections from the manuscript (include quotes in quotation marks "like this" to indicate direct quotes from your manuscript), or elaborate on this item by providing additional information not in the ms, or briefly explain why the item is not applicable/relevant for your study

Your answer

X26-iii) Safety and security procedures

Safety and security procedures, incl. privacy considerations, and any steps taken to reduce the likelihood or detection of harm (e.g., education and training, availability of a hotline)

subitem not at all important

1 ○

2 ○

3 ○

4 ○

5 ○

essential

Does your paper address subitem X26-iii?

Copy and paste relevant sections from the manuscript (include quotes in quotation marks "like this" to indicate direct quotes from your manuscript), or elaborate on this item by providing additional information not in the ms, or briefly explain why the item is not applicable/relevant for your study

Your answer

RESULTS

13a) For each group, the numbers of participants who were randomly assigned, received intended treatment, and were analysed for the primary outcome

NPT: The number of care providers or centers performing the intervention in each group and the number of patients treated by each care provider in each center

Does your paper address CONSORT subitem 13a? *

Copy and paste relevant sections from the manuscript (include quotes in quotation marks "like this" to indicate direct quotes from your manuscript), or elaborate on this item by providing additional information not in the ms, or briefly explain why the item is not applicable/relevant for your study

"Between May 19, 2021, and July 26, 2021, a total of 646 parents were recruited, of whom 320 (49.5%) were assigned to Parent Positive and 326 (50.5%) were assigned to FAU. This was 5% more than planned because of the inherent imprecision of the closing of web-based recruitment. Retention at T2 (466/646, 72.1%) and T3 (442/646, 68.4%) was as expected (see Figure 1 for the CONSORT [Consolidated Standards of Reporting Trials] flow). A number of participants (Parent Positive: 30/320, 9.4%; FAU: 13/326, 4%) were unintentionally contacted in addition between T1 and T2 data collection because of a temporary Qualtrics syntax error; these data were discarded."

13b) For each group, losses and exclusions after randomisation, together with reasons

Does your paper address CONSORT subitem 13b? (NOTE: Preferably, this is shown in a CONSORT flow diagram) *

Copy and paste relevant sections from the manuscript (include quotes in quotation marks "like this" to indicate direct quotes from your manuscript), or elaborate on this item by providing additional information not in the ms, or briefly explain why the item is not applicable/relevant for your study

This is reported in the CONSORT flow diagram.

13b-i) Attrition diagram

Strongly recommended: An attrition diagram (e.g., proportion of participants still logging in or using the intervention/comparator in each group plotted over time, similar to a survival curve) or other figures or tables demonstrating usage/dose/engagement.

subitem not at all important

1 ○

2 ○

3 ○

4 ○

5 ○

essential

Does your paper address subitem 13b-i?

Copy and paste relevant sections from the manuscript or cite the figure number if applicable (include quotes in quotation marks "like this" to indicate direct quotes from your manuscript), or elaborate on this item by providing additional information not in the ms, or briefly explain why the item is not applicable/relevant for your study

Your answer

14a) Dates defining the periods of recruitment and follow-up

Does your paper address CONSORT subitem 14a? *

Copy and paste relevant sections from the manuscript (include quotes in quotation marks "like this" to indicate direct quotes from your manuscript), or elaborate on this item by providing additional information not in the ms, or briefly explain why the item is not applicable/relevant for your study

"Between May 19, 2021, and July 26, 2021, a total of 646 parents were recruited, of whom 320 (49.5%) were assigned to Parent Positive and 326 (50.5%) were assigned to FAU. This was 5% more than planned because of the inherent imprecision of the closing of web-based recruitment. Retention at T2 (466/646, 72.1%) and T3 (442/646, 68.4%) was as expected (see Figure 1 for the CONSORT [Consolidated Standards of Reporting Trials] flow). A number of participants (Parent Positive: 30/320, 9.4%; FAU: 13/326, 4%) were unintentionally contacted in addition between T1 and T2 data collection because of a temporary Qualtrics syntax error; these data were discarded."

14a-i) Indicate if critical “secular events” fell into the study period

Indicate if critical “secular events” fell into the study period, e.g., significant changes in Internet resources available or “changes in computer hardware or Internet delivery resources”

subitem not at all important

1 ◯

2 ◯

3 ◯

4 ◯

5 ◯

essential

Does your paper address subitem 14a-i?

Copy and paste relevant sections from the manuscript (include quotes in quotation marks "like this" to indicate direct quotes from your manuscript), or elaborate on this item by providing additional information not in the ms, or briefly explain why the item is not applicable/relevant for your study

Your answer

14b) Why the trial ended or was stopped (early)

Does your paper address CONSORT subitem 14b? *

Copy and paste relevant sections from the manuscript (include quotes in quotation marks "like this" to indicate direct quotes from your manuscript), or elaborate on this item by providing additional information not in the ms, or briefly explain why the item is not applicable/relevant for your study

Not applicable for this trial.

15) A table showing baseline demographic and clinical characteristics for each group

NPT: When applicable, a description of care providers (case volume, qualification, expertise, etc.) and centers (volume) in each group

Does your paper address CONSORT subitem 15? *

Copy and paste relevant sections from the manuscript (include quotes in quotation marks "like this" to indicate direct quotes from your manuscript), or elaborate on this item by providing additional information not in the ms, or briefly explain why the item is not applicable/relevant for your study

See Tables 1 and 2 in the manuscript.

15-i) Report demographics associated with digital divide issues

In ehealth trials it is particularly important to report demographics associated with digital divide issues, such as age, education, gender, social-economic status, computer/Internet/ehealth literacy of the participants, if known.

subitem not at all important

1 ○

2 ○

3 ○

4 ○

5 ○

essential

Does your paper address subitem 15-i? *

Copy and paste relevant sections from the manuscript (include quotes in quotation marks "like this" to indicate direct quotes from your manuscript), or elaborate on this item by providing additional information not in the ms, or briefly explain why the item is not applicable/relevant for your study

"Baseline characteristics are shown in Tables 1 and 2. The mean child age was 7.45 (SD 1.67) years, with similar proportions of male and female participants; 86.4% (558/646) of parents identified their child as of White ethnicity, 78.8% (509/646) lived in a 2-adult household, and 81.9% (529/646) lived in 1- or 2-child households. A total of 63.9% (413/646) of parents were employed. In total, 4.8% (31/646) had no access to outdoor spaces. A total of 8% (52/646) of children were not attending school in person. The largest between-arm imbalances were observed in work status and living in a household with <4 people."

16) For each group, number of participants (denominator) included in each analysis and whether the analysis was by original assigned groups

16-i) Report multiple “denominators” and provide definitions

Report multiple “denominators” and provide definitions: Report N’s (and effect sizes) “across a range of study participation [and use] thresholds” [1], e.g., N exposed, N consented, N used more than x times, N used more than y weeks, N participants “used” the intervention/comparator at specific pre-defined time points of interest (in absolute and relative numbers per group). Always clearly define “use” of the intervention.

subitem not at all important

1 ◯

2 ◯

3 ◯

4 ◯

5 ◯

essential

Does your paper address subitem 16-i? *

Copy and paste relevant sections from the manuscript (include quotes in quotation marks "like this" to indicate direct quotes from your manuscript), or elaborate on this item by providing additional information not in the ms, or briefly explain why the item is not applicable/relevant for your study

"By T2 and T3, a total of 81.9% (262/320) and 82.8% (265/320) of participants in the Parent Positive arm had accessed the app (see Tables 3 and 4), respectively, with a higher proportion accessing the first few boosters as compared with the last few (eg, 153/262, 58.4% accessing booster 1 vs 45/262, 17.2% accessing booster 5 at T2; Tables S2 and S3 in Multimedia Appendix 1)."
"The primary analysis model included 79.3% (512/646) of participants with at least 1 T2 SDQ conduct score (236/320, 73.8% in Parent Positive and 276/326, 84.7% in FAU)."

16-ii) Primary analysis should be intent-to-treat

Primary analysis should be intent-to-treat, secondary analyses could include comparing only "users", with the appropriate caveats that this is no longer a randomized sample (see 18-i).

subitem not at all important

1 ○

2 ○

3 ○

4 ○

5 ○

essential

Does your paper address subitem 16-ii?

Copy and paste relevant sections from the manuscript (include quotes in quotation marks "like this" to indicate direct quotes from your manuscript), or elaborate on this item by providing additional information not in the ms, or briefly explain why the item is not applicable/relevant for your study

Your answer

17a) For each primary and secondary outcome, results for each group, and the estimated effect size and its precision (such as 95% confidence interval)

Does your paper address CONSORT subitem 17a? *

Copy and paste relevant sections from the manuscript (include quotes in quotation marks "like this" to indicate direct quotes from your manuscript), or elaborate on this item by providing additional information not in the ms, or briefly explain why the item is not applicable/relevant for your study

"Figure 2 shows unadjusted mean profile plots of the outcomes. Table 5 shows summary statistics and between-arm differences (other SDQ subscales are summarized in Table S3 in Multimedia Appendix 1), with standardized group differences displayed in Table 5 and Figure 3. Baseline values for all 5 outcome measures were higher in the Parent Positive arm. Conduct problems (SDQ) were no lower in Parent Positive than in FAU at either T2 (primary outcome; Cohen d=−0.01, 1-sided 95% CI −∞ to 0.20) or T3 (Cohen d=−0.09, 1-sided 95% CI −∞ to 0.03). In contrast, mean emotional symptoms (SDQ) were statistically significantly lower in Parent Positive than in FAU at both T2 (Cohen d=−0.13, 1-sided 95% CI −∞to -0.10) and T3 (Cohen d=−0.13, 1-sided 95% CI −∞ to -0.09). There was no evidence of less parent psychological distress (DASS), parental child-related worries, or family conflict in Parent Positive versus FAU at either time point. Although our preregistered analysis focused on 1-sided tests, the 2-sided P values (Table S4 and Figures S1 and S2 in Multimedia Appendix 1) showed that T3 parental worries were significantly higher in the Parent Positive arm."

17a-i) Presentation of process outcomes such as metrics of use and intensity of use

In addition to primary/secondary (clinical) outcomes, the presentation of process outcomes such as metrics of use and intensity of use (dose, exposure) and their operational definitions is critical. This does not only refer to metrics of attrition (13-b) (often a binary variable), but also to more continuous exposure metrics such as “average session length”. These must be accompanied by a technical description how a metric like a “session” is defined (e.g., timeout after idle time) [1] (report under item 6a).

subitem not at all important

1 ○

2 ○

3 ○

4 ○

5 ○

essential

Does your paper address subitem 17a-i?

Copy and paste relevant sections from the manuscript (include quotes in quotation marks "like this" to indicate direct quotes from your manuscript), or elaborate on this item by providing additional information not in the ms, or briefly explain why the item is not applicable/relevant for your study

Your answer

17b) For binary outcomes, presentation of both absolute and relative effect sizes is recommended

Does your paper address CONSORT subitem 17b? *

Copy and paste relevant sections from the manuscript (include quotes in quotation marks "like this" to indicate direct quotes from your manuscript), or elaborate on this item by providing additional information not in the ms, or briefly explain why the item is not applicable/relevant for your study

Not applicable for this trial.

18) Results of any other analyses performed, including subgroup analyses and adjusted analyses, distinguishing pre-specified from exploratory

Does your paper address CONSORT subitem 18? *

Copy and paste relevant sections from the manuscript (include quotes in quotation marks "like this" to indicate direct quotes from your manuscript), or elaborate on this item by providing additional information not in the ms, or briefly explain why the item is not applicable/relevant for your study

"Service Use, Costs, and Economic Outcomes
Service use and cost data are presented in Tables S5-S7 in Multimedia Appendix 1. Mean service use was low in both arms for all services. The cost of Parent Positive use within the trial was estimated to be £21 per family (Table S1 in Multimedia Appendix 1). Observed total health and social care costs per participant were higher in the Parent Positive arm because of higher mean costs of hospital services for mental health, physical illnesses, and injuries (mean £241, SD £927 in Parent Positive versus mean £196, SD £491 in FAU at T2; mean £136, SD £775 in Parent Positive versus mean £83, SD £269 in FAU at T3; Table S7 in Multimedia Appendix 1). This difference was primarily due to 4 outliers with extremely high costs (99th percentile): 3 (75%) in the Parent Positive arm (with total T2 costs of £3141.25, £3474.25, and £11,381.11) and 1 (25%) in the FAU arm (with total T2 costs of £2836). These outliers were excluded from the economic analysis (see Multimedia Appendix 1 for the analysis, including outliers).
Excluding influential outliers, complete case mean costs were lower in the Parent Positive arm than in the FAU arm (mean £144, SD £326 vs mean £176, SD £445 at T2 and mean £44, SD £104 vs mean £73, SD £445 at T3, respectively; Table S7 in Multimedia Appendix 1).
Complete case CHU9D health state scores increased only very slightly over time in both arms, and between-arm differences in QALYs were negligible (mean 0.0003 SD 0.0019 in Parent Positive vs mean 0.0002 SD 0.0016 in FAU at T2; mean 0.0006 SD 0.0028 in Parent Positive vs mean 0.0003 SD 0.0019 in FAU at T3; Table S8 in Multimedia Appendix 1).
Economic Analysis
The results of the primary, sensitivity, and secondary economic analyses are summarized in Table S9 in Multimedia Appendix 1. Excluding outliers, the Parent Positive arm achieved slightly higher QALYs than the FAU arm (adjusted mean difference=0.000132, SE 0.000193, 95% CI −0.000219 to 0.000536; P=.41) at a lower cost per participant (adjusted mean difference=−£18.89, SE £34.47, 95% CI −£88.35 to £46.79; P=.55; see Figure S3 in Multimedia Appendix 1). The probability of Parent Positive being cost-effective compared with FAU was 72% and 74% at the National Institute for Health and Care Excellence willingness-to-pay thresholds of £20,000 and £30,000 per QALY, respectively (Figure S4 in Multimedia Appendix 1). Results were similar in the sensitivity analyses using the SDQ conduct subscale and including multiple imputation of missing data, as well as for the secondary analysis carried out at T3 (Table S9 in Multimedia Appendix 1). However, the results were sensitive to outliers, with FAU having a higher probability of being cost-effective than Parent Positive for all economic analyses (primary, sensitivity, and secondary) when outliers were included as a result of cost differences in favor of FAU (Table S9 in Multimedia Appendix 1)."

18-i) Subgroup analysis of comparing only users

A subgroup analysis of comparing only users is not uncommon in ehealth trials, but if done, it must be stressed that this is a self-selected sample and no longer an unbiased sample from a randomized trial (see 16-iii).

subitem not at all important

1 ○

2 ○

3 ○

4 ○

5 ○

essential

Does your paper address subitem 18-i?

Copy and paste relevant sections from the manuscript (include quotes in quotation marks "like this" to indicate direct quotes from your manuscript), or elaborate on this item by providing additional information not in the ms, or briefly explain why the item is not applicable/relevant for your study

Your answer

19) All important harms or unintended effects in each group

(for specific guidance see CONSORT for harms)

Does your paper address CONSORT subitem 19? *

Copy and paste relevant sections from the manuscript (include quotes in quotation marks "like this" to indicate direct quotes from your manuscript), or elaborate on this item by providing additional information not in the ms, or briefly explain why the item is not applicable/relevant for your study

"Reported AEs
There were 927 AEs across 53.1% (343/646) of the participants, with 16 (1.7%) serious AEs, none of which were deemed related to trial participation (see Table 6)."

19-i) Include privacy breaches, technical problems

Include privacy breaches, technical problems. This does not only include physical “harm” to participants, but also incidents such as perceived or real privacy breaches [1], technical problems, and other unexpected/unintended incidents. “Unintended effects” also includes unintended positive effects [2].

subitem not at all important

1 ○

2 ○

3 ○

4 ○

5 ○

essential

Does your paper address subitem 19-i?

Copy and paste relevant sections from the manuscript (include quotes in quotation marks "like this" to indicate direct quotes from your manuscript), or elaborate on this item by providing additional information not in the ms, or briefly explain why the item is not applicable/relevant for your study

Your answer

19-ii) Include qualitative feedback from participants or observations from staff/researchers

Include qualitative feedback from participants or observations from staff/researchers, if available, on strengths and shortcomings of the application, especially if they point to unintended/unexpected effects or uses. This includes (if available) reasons for why people did or did not use the application as intended by the developers.

subitem not at all important

1 ○

2 ○

3 ○

4 ○

5 ○

essential

Does your paper address subitem 19-ii?

Copy and paste relevant sections from the manuscript (include quotes in quotation marks "like this" to indicate direct quotes from your manuscript), or elaborate on this item by providing additional information not in the ms, or briefly explain why the item is not applicable/relevant for your study

Your answer

DISCUSSION

22) Interpretation consistent with results, balancing benefits and harms, and considering other relevant evidence

NPT: In addition, take into account the choice of the comparator, lack of or partial blinding, and unequal expertise of care providers or centers in each group

22-i) Restate study questions and summarize the answers suggested by the data, starting with primary outcomes and process outcomes (use)

Restate study questions and summarize the answers suggested by the data, starting with primary outcomes and process outcomes (use).

subitem not at all important

1 ◯

2 ◯

3 ◯

4 ◯

5 ◯

essential

Does your paper address subitem 22-i? *

Copy and paste relevant sections from the manuscript (include quotes in quotation marks "like this" to indicate direct quotes from your manuscript), or elaborate on this item by providing additional information not in the ms, or briefly explain why the item is not applicable/relevant for your study

"Parent Positive was developed as a universal public health intervention to reverse the increase in children's behavioral and emotional problems that occurred in the United Kingdom during the pandemic. In SPARKLE, we tested its effects on children's conduct and emotional problems as well as on parents' psychological distress, child-related worries, and family conflict. We found mixed results.
On the basis of the very high and rapid rate of recruitment into the trial (646 participants in just over 2 months), high levels of retention, and relatively high numbers accessing the app compared with those in trials of similar mental health apps [31], there seemed to be a strong demand for the intervention. This was especially striking when one considers that this was a general population sample unselected on levels of conduct and emotional problems—although the wider Co-SPACE cohort reported fewer conduct problems than those who took part in SPARKLE [5]. Intervention-related AEs were very rare in the study, and the high number of AEs reported in both arms was likely to be due to how these were ascertained in SPARKLE as all parent-reported familial medical and nonmedical AEs were recorded.
Parent Positive did not produce significant reductions in parent-reported children conduct problems after either 1 or 2 months of access. Conduct problems were selected as our primary outcome as one of the main purposes of the app was to give parents advice on common parenting challenges and managing difficult behaviors. Although not statistically significant, the difference in conduct problems between the 2 arms in favor of Parent Positive increased substantially by T3. This was in the context of restrictions easing, schools being open again, and reductions in conduct problems at the general population level being reported [5]. It would be interesting in future trials to extend the intervention period to ≥3 months on the grounds that it may take time for skills to embed and begin to show benefits [32]. Given Parent Positive's emphasis on creating positive environments and experiences for parents and children, we also predicted reductions in children's emotional problems. More specifically, we expected Parent Positive to reduce child worry and raise mood by encouraging the use of praise and other positive strategies, and there is evidence that parent training improves child internalizing problems [33]. Consistent with these predictions, Parent Positive significantly reduced parent-reported child emotional problems at T2 and T3. The complier average causal effect analysis demonstrated that these effects were stronger when the analysis was restricted to those meeting our criteria for sufficient app use (Multimedia Appendix 1).
Although we were unable to model the specific effects of individual boosters on outcomes statistically, an inspection of their use provides some interesting insights. For instance, engagement in the boosters most likely to be associated with reductions in conduct problems (eg, "Promoting better behaviour" and "Using sanctions carefully") was limited and may account for some lack of effect on conduct problems. In contrast, the boosters focusing on parental and more general family-related processes—"Keeping positive and motivated," "Keeping calm when your child acts up," and "Making sure everyone knows what's expected of them"—were the most often accessed. It is possible that these boosters improved the general emotional atmosphere in the family and so were responsible for the significant drop in emotional problems in the Parent Positive arm.
It is important to note that we anticipated that Parent Positive would reduce parenting-related stress both directly because of the app's focus on parental well-being and self-care and indirectly because of any beneficial effects Parent Positive had on children's well-being and the improvements in parenting confidence that we expected this to bring. In contrast, there was a relative preponderance of parental child-related worries in the Parent Positive arm compared with the FAU arm by T3. It is unclear what the source of this effect might be and why it was absent at T2. For example, it may be an unintended

[illegible] consequence of some aspects of the Parent Positive processes or content. Possibilities include challenges related to changing parenting practices and the worry associated with greater awareness of limitations in one’s parenting skills in light of increased parenting knowledge and interactions with other parents and experts. It is also possible that the pattern of app use was different in the first and second months of the trial, explaining why this effect emerged between T2 and T3. However, inspection of Tables S2 and S5 in Multimedia Appendix 1 does not suggest a significant change in app use between these periods.

However, we cannot dismiss the possibility that this is a chance finding or that a different pattern with longer follow-up may emerge—the fact that it was absent at T2 may support this view. It could also be due to the selective retention of parents in the Parent Positive arm with more child-related worries leading the parent to provide more outcome data at T2, although this is not wholly consistent with the intervention-related reductions in parents’ ratings of children’s emotional problems.

What are the practical implications of our findings? The reduction in emotional problems in those allocated to Parent Positive of 0.13 of an SD (at both T2 and T3) would be considered a small effect in a trial of treatment effectiveness in a clinical population, and in such a context, might be dismissed as being of little practical importance. However, we can apply a different standard to community-based mental health interventions such as Parent Positive, where small effects observed at the individual level can make a substantial contribution to the mental health of a whole community [34]; small effects aggregated across many individuals can combine to make a substantial effect overall. In this way, based on these findings, Parent Positive, if implemented across the general UK population, has the potential to make a substantial contribution to reducing emotional problems. However, caution is warranted when drawing such a conclusion based on what is a relatively small sample for a public health trial. Nevertheless, the magnitude of improvement is consistent with findings of 0.07 to 0.16 SDs from a meta-analysis of more intensive but universally targeted interventions that aim to reduce young people’s emotional and behavioral problems [35]. Our finding is particularly striking as many previous interventions studied have been reliant on face-to-face delivery by a trained professional [35]. Given that such interventions are likely to be more logistically challenging and costly to implement than Parent Positive, this highlights the potential health economic benefits of digital parenting approaches and their substantial public health value given their accessibility and universal availability. If offered to families when their children are young and emotional problems have not yet developed or are less entrenched, there is a possibility that Parent Positive could have positive effects on developmental trajectories at a large scale.

Economic analysis was heavily influenced by the extremely high health care–related costs of 4 individuals, the 3 highest of whom were randomized by chance to Parent Positive. This was problematic as service use data were not routinely collected in Co-SPACE and so we were unable to control for baseline service use. We addressed this by removing participants with outlier costs in the 99th percentile. With this, the likelihood of Parent Positive being cost-effective compared with FAU increased from between 19% and 20% to between 73% and 74%. On balance, we would argue that Parent Positive is likely to be cost-effective when implemented across the general population, but this remains to be tested in larger studies where the effect of outliers may be less important or baseline service use data are obtained. In addition, the CHU9D algorithm used to generate QALYs from SDQ scores was based on a clinical child sample. It is unknown whether the algorithm is sufficient for picking up on changes in QALYs in nonclinical child general population samples.

More generally with regard to methodology, the SPARKLE RCT highlights the feasibility and potential value of rapid deployment and evaluation of digital interventions as trials

within general population cohorts with a regular collection of outcome data. This was especially important in light of the pandemic-related restrictions. We built the app, recruited 646 participants, and completed a 2-month data collection period all in 11

22-ii) Highlight unanswered new questions, suggest future research

Highlight unanswered new questions, suggest future research.

subitem not at all important

1

2

3

4

5

essential

Does your paper address subitem 22-ii?

Copy and paste relevant sections from the manuscript (include quotes in quotation marks "like this" to indicate direct quotes from your manuscript), or elaborate on this item by providing additional information not in the ms, or briefly explain why the item is not applicable/relevant for your study

Your answer

20) Trial limitations, addressing sources of potential bias, imprecision, and, if relevant, multiplicity of analyses

20-i) Typical limitations in ehealth trials

Typical limitations in ehealth trials: Participants in ehealth trials are rarely blinded. Ehealth trials often look at a multiplicity of outcomes, increasing risk for a Type I error. Discuss biases due to non-use of the intervention/usability issues, biases through informed consent procedures, unexpected events.

subitem not at all important

1 ○

2 ○

3 ○

4 ○

5 ○

essential

Does your paper address subitem 20-i? *

Copy and paste relevant sections from the manuscript (include quotes in quotation marks "like this" to indicate direct quotes from your manuscript), or elaborate on this item by providing additional information not in the ms, or briefly explain why the item is not applicable/relevant for your study

"The RCT was conducted during spring 2021 and summer 2021, when pandemic-related restrictions were beginning to ease (ie, children were not homeschooled en masse, and restrictions on social contacts had relaxed). In this sense, we are unable to generalize our findings to more restrictive lockdown periods or to nonrestricted contexts, and parents of children in Co-SPACE reported a drop in conduct problems when restrictions eased [5]. However, during the RCT, there was continued concern and uncertainty about the course of the pandemic and its continued impact. Second, we had differential dropout between arms, with higher dropout in the Parent Positive arm. This is possibly due to the families in the FAU arm waiting for posttrial access to Parent Positive and, therefore, being more engaged in the data collection process. Third, the Co-SPACE cohort and, therefore, the SPARKLE subsample are underrepresentative of ethnic minority groups and low-income families. The data in Table 1 indicate that the sample had a somewhat higher proportion of White British participants (558/646, 86.4% of children and 584/646, 90.4% of parents) than the UK population as a whole (82%) and were less likely to be living on very low incomes (59/646, 9.1% on <£16,000 per year [SPARKLE] vs 22% [United Kingdom]) [36]. Finally, the overall number of Parent Positive users was too low to establish the real value of the Parenting Exchange. The 90-9-1 principle of engagement within online communities explains that 90% of users observe but do not engage in web-based exchanges, 9% provide some contribution, and 1% of users generate most of the content [37]. Therefore, in the SPARKLE trial, where the app had <300 users, the level of engagement with the Parenting Exchange was unsurprisingly low. Nevertheless, we should not discount the potential value of Parenting Exchange for parents who might be seeking social connections and shared understanding within a safe and supportive online community."

21) Generalisability (external validity, applicability) of the trial findings

NPT: External validity of the trial findings according to the intervention, comparators, patients, and care providers or centers involved in the trial

21-i) Generalizability to other populations

Generalizability to other populations: In particular, discuss generalizability to a general Internet population, outside of a RCT setting, and general patient population, including applicability of the study results for other organizations

subitem not at all important

1 ○

2 ○

3 ○

4 ○

5 ○

essential

Does your paper address subitem 21-i?

Copy and paste relevant sections from the manuscript (include quotes in quotation marks "like this" to indicate direct quotes from your manuscript), or elaborate on this item by providing additional information not in the ms, or briefly explain why the item is not applicable/relevant for your study

Your answer

21-ii) Discuss if there were elements in the RCT that would be different in a routine application setting

Discuss if there were elements in the RCT that would be different in a routine application setting (e.g., prompts/reminders, more human involvement, training sessions or other co-interventions) and what impact the omission of these elements could have on use, adoption, or outcomes if the intervention is applied outside of a RCT setting.

subitem not at all important

1 ○

2 ○

3 ○

4 ○

5 ○

essential

Does your paper address subitem 21-ii?

Copy and paste relevant sections from the manuscript (include quotes in quotation marks "like this" to indicate direct quotes from your manuscript), or elaborate on this item by providing additional information not in the ms, or briefly explain why the item is not applicable/relevant for your study

Your answer

OTHER INFORMATION

23) Registration number and name of trial registry

Does your paper address CONSORT subitem 23? *

Copy and paste relevant sections from the manuscript (include quotes in quotation marks "like this" to indicate direct quotes from your manuscript), or elaborate on this item by providing additional information not in the ms, or briefly explain why the item is not applicable/relevant for your study

"Trial Registration: ClinicalTrials.gov NCT04786080;
https://clinicaltrials.gov/ct2/show/NCT04786080"

24) Where the full trial protocol can be accessed, if available

Does your paper address CONSORT subitem 24? *

Cite a Multimedia Appendix, other reference, or copy and paste relevant sections from the manuscript (include quotes in quotation marks "like this" to indicate direct quotes from your manuscript), or elaborate on this item by providing additional information not in the ms, or briefly explain why the item is not applicable/relevant for your study

"The published protocol is available here [15]."

25) Sources of funding and other support (such as supply of drugs), role of funders

Does your paper address CONSORT subitem 25? *

Copy and paste relevant sections from the manuscript (include quotes in quotation marks "like this" to indicate direct quotes from your manuscript), or elaborate on this item by providing additional information not in the ms, or briefly explain why the item is not applicable/relevant for your study

"The SPARKLE trial was funded by the Economic and Social Research Council: Grant ES/V016393/1 from the, UK. Co-SPACE was funded by grants from UKRI (2004CQ002/BS3) and the Westminster Foundation. The animations used in the Parenting Boosters were produced through funding by The Maudsley Charity and the South London and Maudsley NHS Foundation Trust. KG and ESBs contributions represent, respectively, independent research part funded by the National Institute for Health and Care Research (NIHR) Applied Maudsley Biomedical Research Centre at South London and Maudsley NHS Foundation Trust and the NIHR Applied Research Collaboration South London (NIHR ARC South London) at King’s College Hospital NHS Foundation Trust. CC receives funding from the NIHR Applied Research Collaboration Oxford and Thames Valley at Oxford Health NHS Foundation Trust. PW receives funding through a NIHR Development and Skills Enhancement award. The views expressed are those of the author(s) and not necessarily those of the NHS, the NIHR or the Department of Health and Social Care."

X27) Conflicts of Interest (not a CONSORT item)

X27-i) State the relation of the study team towards the system being evaluated

In addition to the usual declaration of interests (financial or otherwise), also state the relation of the study team towards the system being evaluated, i.e., state if the authors/evaluators are distinct from or identical with the developers/sponsors of the intervention.

subitem not at all important

1 ○

2 ○

3 ○

4 ○

5 ○

essential

Does your paper address subitem X27-i?

Copy and paste relevant sections from the manuscript (include quotes in quotation marks "like this" to indicate direct quotes from your manuscript), or elaborate on this item by providing additional information not in the ms, or briefly explain why the item is not applicable/relevant for your study

Your answer

About the CONSORT EHEALTH checklist

As a result of using this checklist, did you make changes in your manuscript? *

- ○ yes, major changes
- ○ yes, minor changes
- ● no

What were the most important changes you made as a result of using this checklist?

Your answer

How much time did you spend on going through the checklist INCLUDING making changes in your manuscript *

No changes were made, but it took an hour to copy and paste the text into this form.

As a result of using this checklist, do you think your manuscript has improved? *

- ◯ yes
- ◉ no
- ◯ Other:

Would you like to become involved in the CONSORT EHEALTH group?

This would involve for example becoming involved in participating in a workshop and writing an "Explanation and Elaboration" document

- ◯ yes
- ◉ no
- ◯ Other:

Clear selection

Any other comments or questions on CONSORT EHEALTH

Your answer

STOP - Save this form as PDF before you click submit

To generate a record that you filled in this form, we recommend to generate a PDF of this page (on a Mac, simply select "print" and then select "print as PDF") before you submit it.

When you submit your (revised) paper to JMIR, please upload the PDF as supplementary file.

Don't worry if some text in the textboxes is cut off, as we still have the complete information in our database. Thank you!

Final step: Click submit !

Click submit so we have your answers in our database!

Submit Clear form

Never submit passwords through Google Forms.

This content is neither created nor endorsed by Google. Report Abuse - Terms of Service - Privacy Policy

Google Forms
